# Transcriptome-wide stability analysis uncovers LARP4-mediated *NFκB1* mRNA stabilization during T cell activation

**DOI:** 10.1093/nar/gkaa643

**Published:** 2020-07-31

**Authors:** Yi Tian, Zhouhao Zeng, Xiang Li, Yiyin Wang, Runsen Chen, Sandy Mattijssen, Sergei Gaidamakov, Yuzhang Wu, Richard J Maraia, Weiqun Peng, Jun Zhu

**Affiliations:** Department of Physics, George Washington University, Washington, DC 20052, USA; Systems Biology Center, National Heart Lung and Blood Institute, National Institutes of Health, Bethesda, MD 20892, USA; Institute of Immunology, PLA, Third Military Medical University, Chongqing 400038, PR China; Department of Physics, George Washington University, Washington, DC 20052, USA; Department of Physics, George Washington University, Washington, DC 20052, USA; Systems Biology Center, National Heart Lung and Blood Institute, National Institutes of Health, Bethesda, MD 20892, USA; Systems Biology Center, National Heart Lung and Blood Institute, National Institutes of Health, Bethesda, MD 20892, USA; Department of Cardiothoracic Surgery, Children's Hospital of Nanjing Medical University, Nanjing 210008, China; Eunice Kennedy Shriver National Institute of Child Health and Human Development, National Institutes of Health, Bethesda, MD, USA; Eunice Kennedy Shriver National Institute of Child Health and Human Development, National Institutes of Health, Bethesda, MD, USA; Institute of Immunology, PLA, Third Military Medical University, Chongqing 400038, PR China; Eunice Kennedy Shriver National Institute of Child Health and Human Development, National Institutes of Health, Bethesda, MD, USA; Department of Physics, George Washington University, Washington, DC 20052, USA; Systems Biology Center, National Heart Lung and Blood Institute, National Institutes of Health, Bethesda, MD 20892, USA

## Abstract

T cell activation is a well-established model for studying cellular responses to exogenous stimulation. Motivated by our previous finding that intron retention (IR) could lead to transcript instability, in this study, we performed BruChase-Seq to experimentally monitor the expression dynamics of nascent transcripts in resting and activated CD4^+^ T cells. Computational modeling was then applied to quantify the stability of spliced and intron-retained transcripts on a genome-wide scale. Beyond substantiating that intron-retained transcripts were considerably less stable than spliced transcripts, we found a global stabilization of spliced mRNAs upon T cell activation, although the stability of intron-retained transcripts remained relatively constant. In addition, we identified that La-related protein 4 (LARP4), an RNA-binding protein (RBP) known to enhance mRNA stability, was involved in T cell activation-dependent mRNA stabilization. Knocking out *Larp4* in mice destabilized *Nfκb1* mRNAs and reduced secretion of interleukin-2 (IL2) and interferon-gamma (IFNγ), two factors critical for T cell proliferation and function. We propose that coordination between splicing regulation and mRNA stability may provide a novel paradigm to control spatiotemporal gene expression during T cell activation.

## INTRODUCTION

The activation of CD4^+^ T cells is vital for the immune response (1,2). When obtaining proper signals, such as CD3 and CD28, resting T cells can transition from a relatively static state to an active proliferating state, leading to the production of cytokines. One of them is interleukin 2 (IL2), which promotes T cell proliferation (2). Both transcriptional and posttranscriptional regulations are critical for promoting the immune response that is capable of eliminating an infection while restricted enough to prevent inflammatory injury (3–8). In general, the rates of transcription and mRNA degradation determine the abundance of each mRNA, enabling global changes in gene expression and underpinning dynamic cellular responses. Transcriptional regulation during T cell activation has been well characterized. By contrast, mRNA stability during T cell activation, which has only recently emerged as an important mechanism to control inflammatory gene expression, has been far less well characterized (8–12).

Intron retention (IR) is one of the dominant forms of alternative splicing in eukaryotes (13–17). Our previous study demonstrated that IR is prevalent in resting CD4^+^ T cells and dramatically decreases upon cell activation. We provided initial evidence that IR could lead to transcript instability, serving as a significant mechanism for posttranslational gene regulation (18). Similar phenomena have also been observed in other systems (17,19,20). To date, there is no genome-wide study to directly measure the stability of intron-retained transcripts, calling for a systematic approach to compare IR and spliced transcripts on a global scale.

Three approaches have been used to evaluate RNA stability in T cells, including transcriptional inhibition (6), nuclear run-on assay (4) and pulsed labeling with nucleotide analogs, which are incorporated into nascent transcripts without disturbing normal cell metabolism (21). Analysis of the dynamic relationship between labeled and unlabeled transcripts was adopted to assess mRNA stability as well as the rate of nascent RNA synthesis (21–28).

In this study, we utilized BruChase-Seq to investigate the dynamics of mRNA degradation upon CD4^+^ T cell activation. Using bipartite RNA stability modeling, we confirmed that spliced transcripts were more stable than intron-retained transcripts. Surprisingly, we found that the overall stability of spliced mRNAs was increased upon T cell activation, while the stability of intron-retained transcripts was independent of cell activation. We provided evidence that the decrease in steady-state IR level in activated CD4^+^ T cells was partially due to the increased splicing efficiency and further stabilization of the spliced transcripts. Further integration of RNA-seq, ChIP-seq and BruChase-seq data allowed us to identify a subset of genes predominately regulated at the RNA stability level. One prominent example was *nuclear factor kappa-light-chain-enhancer of activated B cells 1 (NFκB1)*, a crucial transcriptional regulator required for proper CD4^+^ T cell differentiation and function. Last, we identified La-related protein 4 (LARP4), as a key RNA binding protein (RBP) underlying mRNA stabilization upon T cell activation. Using a *Larp4* knockout mouse model, we established that LARP4 stabilized *Nfκb1* mRNA and promoted expression of *Il2 and interferon gamma (Ifnγ*).

## MATERIALS AND METHODS

### CD4^+^ T cell isolation and activation

Human resting CD4^+^ T cells were isolated from the peripheral blood monocytes (PBMCs) of anonymous healthy donors with the Dynabeads Untouched Human CD4^+^ T Cells Kit (Invitrogen), followed by activation using the Dynabeads Human T-Activator CD3/CD28 for T Cell Expansion and Activation Kit (Invitrogen) by incubation at 37°C for 18 h (18,29). The cell morphology was checked under the microscope. Human blood samples were obtained from the US National Institutes of Health (NIH) Blood Bank and all the studies were performed under the research donor protocol sponsored by the NIH Clinical Center.

The *Larp4* gene knockout (KO) mice (12–16 months) were maintained in the Maraia Laboratory (30). The resting CD4^+^ T cells from WT and *Larp4* KO mice were isolated from the mouse spleen using the Dynabeads Untouched Mouse CD4^+^ T Cells Kit (Invitrogen), followed by activation using anti-CD3/CD28 antibodies for 18 h at 37°C. All mouse studies were performed at the NIH under protocol ASP 10–005 and approved by the IACUCs of NICHD.

### Bru-seq and BruChase-Seq

Bromouridine (BrU, Aldrich, cat# 850187) was added to the culture media of 10 million resting or activated CD4^+^ T cells to a final concentration of 2 mM. After incubation at 37°C for 1 h, the cells were washed three times with PBS and either collected directly (nascent RNA, Bru-Seq) or chased in the conditioned cell-culture media containing 20 mM uridine for 0.5 h or 2 h at 37°C (0.5 h or 2 h RNA, BruChase-Seq) (24,27). Total RNA was prepared using TRIzol Reagent (Invitrogen), and cytoplasmic RNA was isolated as described in (31). BrU labeled RNA was isolated from the total RNA or cytoplasmic RNA by anti-BrdU antibodies (BD Biosciences, cat# 555627) or mouse IgG (BD Biosciences, cat# 555746) conjugated to Dynabeads Goat anti-Mouse IgG (Invitrogen, cat# 110.33) (24,27). The isolated BrU-labeled RNA was used for constructing strand-specific RNA-Seq library with the Illumina TruSeq Kit (Illumina) according to the manufacturer's instructions. Raw sequencing data were acquired with an Illumina HiSeq-3000 at the DNA Sequencing and Genomic Core, NHLBI, NIH.

### Mapping of sequencing reads

The quality of the Bru-seq and BruChase-Seq libraries was assessed by FastQC v0.10.1 (http://www.bioinformatics.babraham.ac.uk/projects/fastqc/). Raw sequencing reads were mapped to the respective genome using TopHat (v2.1.0) (32). UCSC genes from the iGenome hg19 assemblies (http://support.illumina.com/sequencing/sequencing_software/igenome.html) were used for gene annotation for human data. Only uniquely mapped reads were used for downstream analyses.

### Calculation of the IRI

For each gene, all annotated transcript isoforms were consolidated, and the intronic regions shared by all isoforms were designated as constitutive intronic regions (CIRs). Constitutive exonic regions (CERs) were defined in a similar manner. In addition, the exon–intron junctions between CIRs and CERs were defined as constitutive junctions (CJs). The IRI of a CIR was defined as the ratio of its read density to the read density of its adjacent CERs; the IRI of a gene was defined as the ratio of the overall read density of CIRs in that gene to the overall read density of CERs.

In all other texts, by introns (or intronic regions), exons (or exonic regions) and junctions, we meant CIRs, CERs and CJs for convenience, respectively.

### Genome-wide IRI

Genome-wide IRI characterizes the global IR level in an RNA-Seq library. The value was determined as follows: the genome-wide read density in intronic regions was defined as the ratio of the total number of intronic reads to the total length of the intronic region. Similarly, genome-wide read density in exonic regions was defined as the ratio of the total number of exonic reads to the total length of the exonic region. Genome-wide IRI was defined as the ratio of the genome-wide read density of intronic regions to the genome-wide read density of exonic regions.

### Normalization of BruChase-Seq data

BruChase-Seq data derived from the total RNA or cytoplasmic RNA were normalized by a set of 9 (nine) house-keeping genes (24,33), which were experimentally validated for having long half-lives (much longer than the time scale of our pulse-chase experiment) in both resting and activated T cells. Briefly, a BrU-labeled RNA (External RNA Controls Consortium (ERCC)-00002) was *in vitro* transcribed (TranscriptAid T7 High Yield Transcription Kit, Thermo Scientific). For each BruChase-seq condition, the BrU-labeled control RNA was spiked into cell pellet lysed in trizol before total RNA or cytoplasmic RNA extraction at equal amount. After immunoprecipitation and reverse-transcription, qPCR was used to survey 12 housekeeping genes (*B2M, GAPDH, HPRT1, ACTB, SDHA, HSP90AB1, YWHAB, YWHAQ, YWHAZ, PTMA, UBC* and *TBP*) normalized to BrU-labeled ERCC-00002. Of these transcripts, nine (*B2M, GAPDH, HPRT1, ACTB, SDHA, HSP90AB1, YWHAB, YWHAQ* and *PTMA*, Supplementary Figure S1) were found to be stable and served as endogenous normalization controls. Primers for all the validated genes are listed in Supplementary Table S1. The read counts of nine housekeeping loci derived from total RNA or cytoplasmic RNA BruChase-seq were then used to determine the normalization factors among samples:

$$\begin{eqnarray*} &&Normalization\ factor \nonumber \\ &&= \frac{{1,000,000}}{{\ Total\ CER\ read\ counts\ of\ housekeeping\ genes}} \end{eqnarray*}$$

For each gene, the reads per kilobase per million (RPKM) of each time point was further normalized by multiplying with the corresponding normalization factor.

### Model estimating transcript degradation rates

Two modeling methods were used for the calculation of degradation rates in resting and activated CD4^+^ T cells by comparing BrU-labeled RNA between 0.5 and 2 h time points as following:

1. One-component model
  We used a first-degree model that directly connects RNA expression with the transcription and degradation rates to fit the BruChase-Seq data. Let α be the transcription rate (RPKM/min), β the degradation rate (min^−1^), and X the expression level of a gene × (RPKM). In the first-degree model, the dynamics of *X* were determined by:
  $$\begin{equation*}\frac{{dX}}{{dt}} = \alpha \left( t \right) - \beta \left( t \right)X\end{equation*}$$
  In BruChase-Seq, BrU was washed away after Bru labeling so that we assumed no additional labeled transcripts were produced (*α* = 0). Moreover, we assumed that *β* was constant.
  Given the above assumptions:
  $$\begin{equation*}X \left( t \right) = \hat{X} \left( {{t_0}} \right){e^{ - \beta \left( {t - {t_0}} \right)}}\end{equation*}$$
  We then used the experimentally measured *X*(*t_i_*) to estimate *β* using weighted least-square fitting for each gene:
  $$\begin{equation*}\ln X \left( t \right) = \ln \hat{X}\left( {{t_0}} \right) - \beta \left( {t - {t_0}} \right)\end{equation*}$$
  $$\begin{eqnarray*} &&\beta :{\rm{minimize}}\mathop \sum \limits_{{t_i}} X\left( {{t_i}} \right) \nonumber \\ &&\times\, \left[ {\ln X\left( {{t_i}} \right) - \ln \hat{X}\left( {{t_0}} \right) + \beta \left( {{t_i} - {t_0}} \right)} \right]^2 \end{eqnarray*}$$
  The conventional one-component model assumed that all nascent transcripts of a gene had the same degradation rate. The degradation rates (overall *β*) could be estimated using the above equation.
2. Mixture model estimating the degradation rates of spliced and intron-retained transcripts
  Considering the effect of IR on transcriptional degradation, we assumed that the transcripts were a mixture of two types of mRNAs: spliced and intron-retained. They have potentially distinct degradation rates: *β*_S_ for spliced mRNAs and *β*_IR_ for intron-retained mRNAs. Letting *X*_1_ be the expression level of the spliced mRNAs of a gene *x* and letting *X*_2_ be the expression level of the intron-retained mRNAs of the gene *x*, we had:
  $$\begin{equation*} \frac{{d{X_1}}}{{dt}} = - {\beta _S}{X_1}\end{equation*}$$
  $$\begin{equation*} \frac{{d{X_2}}}{{dt}} = - {\beta _{IR}}{X_2}\end{equation*}$$
  $$\begin{equation*}{X_1} \left( t \right) = {\hat{X}_1}\left( {{t_0}} \right){e^{ - {\beta _{\rm{S}}}\left( {t - {t_0}} \right)}} \end{equation*}$$
  $$\begin{equation*}{X_2} \left( t \right) = {\hat{X}_2} \left( {{t_0}} \right){e^{ - {\beta _{{\rm{IR}}}}\left( {t - {t_0}} \right)}}\end{equation*}$$
  They were associated with experimentally measured expression levels and IRI levels as follows:
  $$\begin{equation*}X \left( t \right) = {X_1} \left( t \right) + {X_2}\left( t \right)\end{equation*}$$
  $$\begin{equation*}IRI \left( t \right) = \frac{{{X_2}\left( t \right)}}{{{X_1}\left( t \right) + {X_2}\left( t \right)}} \end{equation*}$$
  We could use weighted least-square fitting to estimate *β*_S_ and *β*_IR_ for each gene with IR:
  $$\begin{eqnarray*} &&{\beta _{\rm{S}}},{\beta _{{\rm{IR}}}}:{\rm{minimize}}\mathop \sum \limits_{{t_i}} {X_1}\left( {{t_i}} \right)\nonumber \\ &&\times\, \left[ {\ln {X_1}\left( {{t_i}} \right) - \ln {{\hat{X}}_1}\left( {{t_0}} \right) + {\beta _{\rm{S}}}\left( {{t_i} - {t_0}} \right)} \right]^2 \nonumber \\ && +\, {X_2}\left( {{t_i}} \right)\left[ {\ln {X_2}\left( {{t_i}} \right) - \ln {{\hat{X}}_2}\left( {{t_0}} \right) + {\beta _{{\rm{IR}}}}\left( {{t_i} - {t_0}} \right)} \right]^2 \end{eqnarray*}$$
  In Figure 2B, the expressed genes met the requirements: (i) IRI ≤ 1 at any given time point in resting T cells; (ii) degradation rates can be determined by total RNA BruChase-seq in resting T cells. There were 7999 expressed genes derived from the two-component model or 8395 expressed genes derived from the conventional one-component model.

### Exon–exon and exon–intron junction reads

Junction reads that straddle two exons were called exon-exon junction reads (*X*_EE_) (spliced reads), and those that straddle an exon and an intron were called exon–intron junction reads (*X*_EI_). We required an exon-intron junction read to cover the junction point and overlap both sides for at least 8 bp. We required an exon-exon junction read to jump over the junction point and overlap respective exons for at least 8 bp.

### Estimating β_S_ and β_IR_ using junction reads

Exon–exon junction reads (*X*_EE_) and exon–intron junction reads (*X*_EI_) could be used to approximate the spliced and intron-retained transcripts, respectively:

$$\begin{eqnarray*} &&{\beta _{\rm{S}}},{\beta _{{\rm{IR}}}} : {\rm{minimize}}\mathop \sum \limits_{{t_i}} {X_{{\rm{EE}}}}\left( {{t_i}} \right) \nonumber \\ &&\times\,\left[ {\ln {X_{{\rm{EE}}}}\left( {{t_i}} \right) - \ln {{\hat{X}}_{EE}}\left( {{t_0}} \right) + {\beta _{\rm{S}}}\left( {t - {t_0}} \right)} \right]^2 \nonumber \\ && +\, X_{{\rm{EI}}}\left( {{t_i}} \right)\left[ {\ln {X_{{\rm{EI}}}}\left( {{t_i}} \right) - \ln {{\hat{X}}_{EI}}\left( {{t_0}} \right) + {\beta _{{\rm{IR}}}}\left( {t - {t_0}} \right)} \right]^2 \end{eqnarray*}$$

### Calculation of the percentage of spliced transcripts (PST)

The PST was used to approximate the splicing efficiency of individual genes and defined as the gene IRI level at 0.5 h in BruChase-Seq data derived from total RNA. Genes with differential splicing efficiency between different conditions were selected using a PST difference ≥5% as a cutoff.

### Evaluation of the transcription rate

The transcription rate of a gene was estimated by its expression (RPKM) in the Bru-Seq data derived from total RNA. Genes with differential transcription rates were identified by requiring >1.5-fold changes in the aforementioned RPKM values.

### Definition of upregulated genes upon T cell activation

Bulk RNA-seq data sets of human resting and activated CD4^+^ T cells from our previous studies were used to define the upregulated genes (SRP058500) (18). Differentially expressed genes were selected by requiring >1.5 expression fold change and RPKM >1 in at least one of the conditions.

### Chromatin immunoprecipitation sequencing (ChIP-Seq) analysis

RNA Pol II (Pol II) ChIP-Seq data from our previous studies (the NCBI Sequence Read Archive with SRA number SRP058500) were mapped to the genome using Bowtie 2 (34). SICER was used to determine ChIP-enriched regions with the parameter settings: window size = 200 bp, gap size = 400 bp, and false discovery rate (FDR) = 0.01 (35). Only reads located in ChIP-enriched regions were used for downstream analysis. The Pol II level of a gene was defined as the RPKM of Pol II reads in the gene body region. Genes with differential Pol II occupancy were identified by requiring a >1.5-fold change in the Pol II level.

### Signal transduction pathway analysis

Signal transduction pathway analysis was carried out using the GeneRanker package (Genomatrix) with default parameters. Results were further filtered by the NetPath signal transduction pathways (36).

### Identification of RBPs with significant binding in the 3′ UTR of β_S_-decreased genes

We prepared two sets of genes as follows. We ranked all 3944 genes upregulated in T cell activation according to the relative change in stability [i.e. (*β*_s, active_ – *β*_s, resting_)/*β*_s, resting_], and chose 1000 genes with the most increase in stability upon activation and 1000 genes with the least increase (or decrease) in stability upon activation. The eCLIP data of RBPs in the K562 cell line were downloaded from the ENCODE project (https://www.encodeproject.org/). For each RBP, we estimated its binding score of each gene in the two gene sets by adding the binding scores of all eCLIP peaks located in the 3′UTR region. A Wilcoxon rank-sum test was performed to evaluate the significance of the difference between binding ranking in the two sets of genes. The RBPs were ranked according to the significance of the difference.

### Reverse transcription and quantitative PCR (RT-qPCR)

RT-qPCR was used to validate the stability change in candidate transcripts upon T cell activation. To detect spliced transcripts, a pair of exonic primers were used by spanning at least one intron (>1000 bp), and an exonic primer and an intronic primer were used to detect intron-retained transcripts. Primers were designed by Primer3 (version 0.4.0) and synthesized by IDT. Other detailed information was described previously (18). Primers for all the validated genes are listed in Supplementary Tables S1 and S2.

### ChIP-qPCR

ChIP coupled with qPCR assays were performed as described previously (37). A monoclonal anti-Pol II (ab76123, Abcam) antibody was used for immunoprecipitation. Primers for the *NFκB1, CRKL, IL2* and *STAT1* genes are listed in Supplementary Tables S3 and S4.

### Cross-linking immunoprecipitation-qPCR (CLIP-qPCR)

CLIP-qPCR assays were performed as described previously (38). A polyclonal anti-human LARP4 (A303-900A, Bethyl) antibody was used for immunoprecipitation. Primers for the *NFκB1* and *CRKL* genes are listed in Supplementary Table S5.

### Cytokine production by resting and activated mouse CD4^+^ T cells

The culture supernatants of resting and activated mouse CD4^+^ T cells derived from the WT and *Larp4* KO mice were examined for cytokine production using the LEGENDplex™ Mouse Th Cytokine Panel (13-plex, BioLegend) according to the manufacturer's instructions and analyzed using FACSAria III.

### The profile of LARP4 binding flanking the end of the 3′ UTR

The profile of eCLIP-Seq LARP4 peaks flanking the end of 3′ UTRs in K562 cells was generated as follows: The called peaks of eCLIP targeting LARP4 protein were retrieved from ENCODE (ENCSR888YTT). There were two isogenic replicates (ENCFF259HTD, ENCFF986MGC). Peaks shared by the two replicates (18368 in total) were retained for downstream analysis. The normalized density of LARP4 binding events, expressed as the number of binding events per kilobase per million binding events, were plotted with respect to the distance away from the polyA cleavage site.

### Statistical analysis

Statistical analysis was performed with Prism 6.0 (GraphPad). Paired (for RT-qPCR, ChIP-qPCR and CLIP-qPCR experiments) two-tailed *t* test with 95% confidence interval was used to calculate *P* value. The number of individual experiments is listed in the legend. Graphs show individual samples and center values indicate mean. Significance was defined as *P* < 0.05.

## RESULTS

### BruChase-Seq measured the genome-wide mRNA degradation rate of resting and activated CD4^+^ T cells

**Figure 1.**
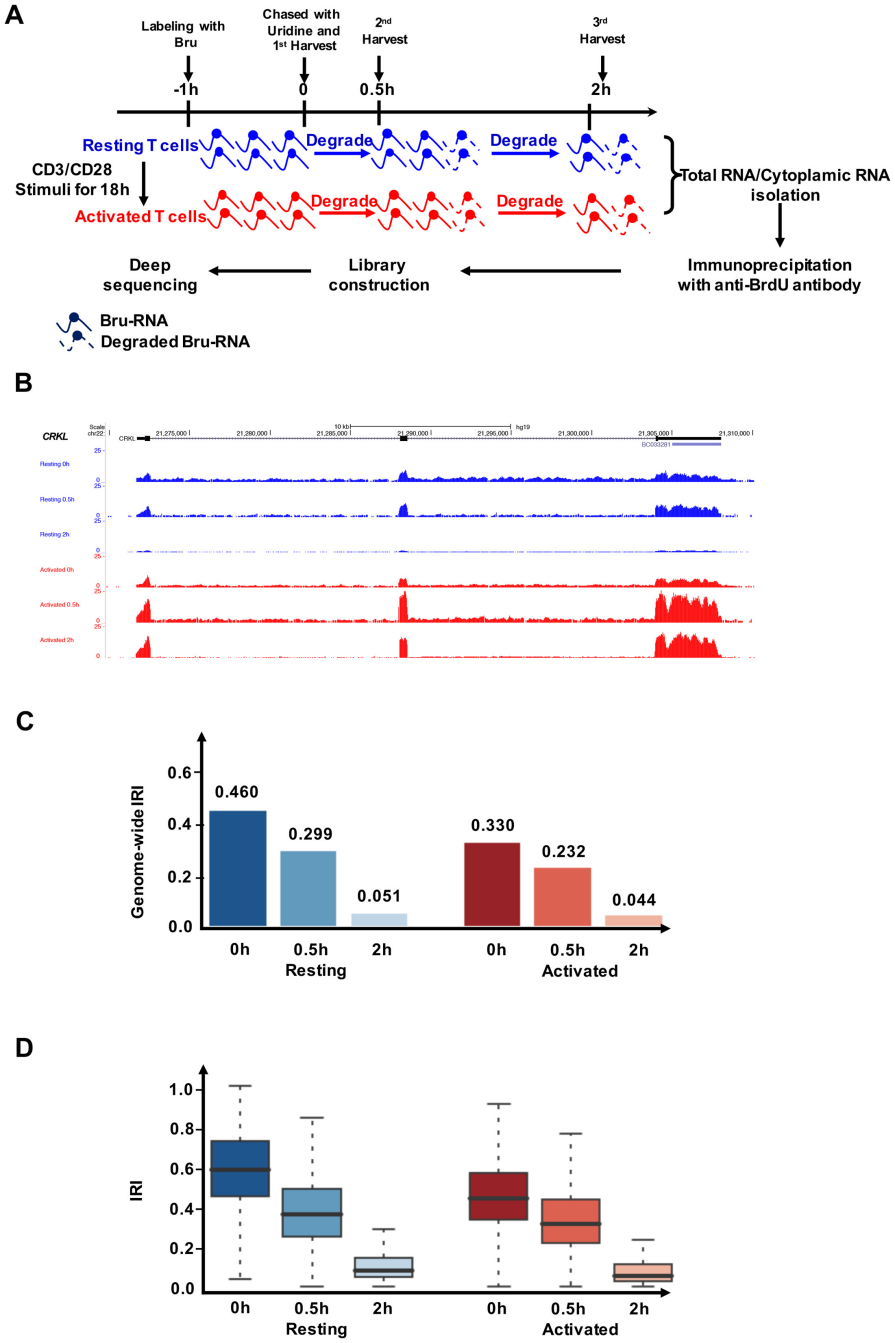
Bru-seq and BruChase-Seq analysis of CD4^+^ T cell transcriptomes. (**A**) Schematic overview of the Bru-seq and BruChase-Seq procedure. Both resting and activated CD4^+^ T cells were exposed to Bromouridine (BrU), and nascent RNAs were labeled with BrU for 1 h. Cells were either collected directly or chased in uridine-containing medium for 0.5 and 2 h, respectively. Bru-labeled RNAs were then immunoprecipitated from total RNA and subjected to deep sequencing analysis. (**B**) Normalized read-profiles of BrU-labeled transcripts for the *CRKL* gene at different chase-time points in resting and activated human CD4^+^ T cells. For intron 1, the IRI values were 0.208, 0.067, 0.034, 0.139, 0.066, 0.005 (top to bottom). For intron 2, the IRI values were 0.417, 0.081, 0.115, 0.234, 0.106, 0.024 (top to bottom). (C and D) Genome-wide intron retention index (IRI, see Method for details) (**C**) and distribution of gene-level IRI values (**D**) at different chase-time points for the resting and activated states. In (**D**), the gene-level IRI values in the resting T cells were significantly higher than those in activated T cells at each time point (0h, *P* < 1E–300; 0.5 h, *P* = 5.0 E–54; 2h, *P* = 2.1 E–125.one-sided Mann–Whitney *U* test).

To investigate mRNA stability during CD4^+^ T cell activation, we employed BruChase-Seq, a high-throughput sequencing method for monitoring the RNA degradation rate by pulse-chase labeling of nascent transcripts (24,27). Resting and activated human primary CD4^+^ T cells were labeled with bromouridine (BrU) for 1 h. Cells were either collected directly or harvested for 0.5 or 2 h after switching back to uridine-containing culture medium. Labeled transcripts were immunoprecipitated from total RNA and subsequently analyzed by RNA-Seq (Figure 1A, see Materials and Methods for details). Quality assessment of RNA enrichment by RT-qPCR suggested the contribution of nonspecific RNA pull-down in our data was negligible (Supplementary Figure S2). Since BrU was incorporated during active transcription, both exons and introns of nascent transcripts were labeled with the modified nucleotide. However, the average read density was significantly higher for exons than for introns, even at the zero-hour chase-time point. This observation was consistent with the notion of co-transcriptional splicing (Figure 1B), as introns are quickly degraded by exonucleases when excised from pre-messenger RNAs (39,40). We previously showed that IR was a prevalent phenomenon in resting CD4^+^ T cells, and a reduction in IR was a hallmark of CD4^+^ T cell activation (18). BruChase-Seq recapitulated the phenomena in that the IR level was significantly reduced in the activated T cells compared to that of resting T cells at the 0.5-h chase-time point (Figure 1C and D). Because pre-mRNA splicing is a quick process (40), we assumed that at the 0.5 h chase-time point the splicing of most pulse-labeled transcripts was completed. Therefore, sequencing reads observed at intronic regions at 0.5 and 2 h might reflect *bona fide* IR (Figure 1B).

### Intron-retained transcripts were less stable than spliced transcripts

**Figure 2.**
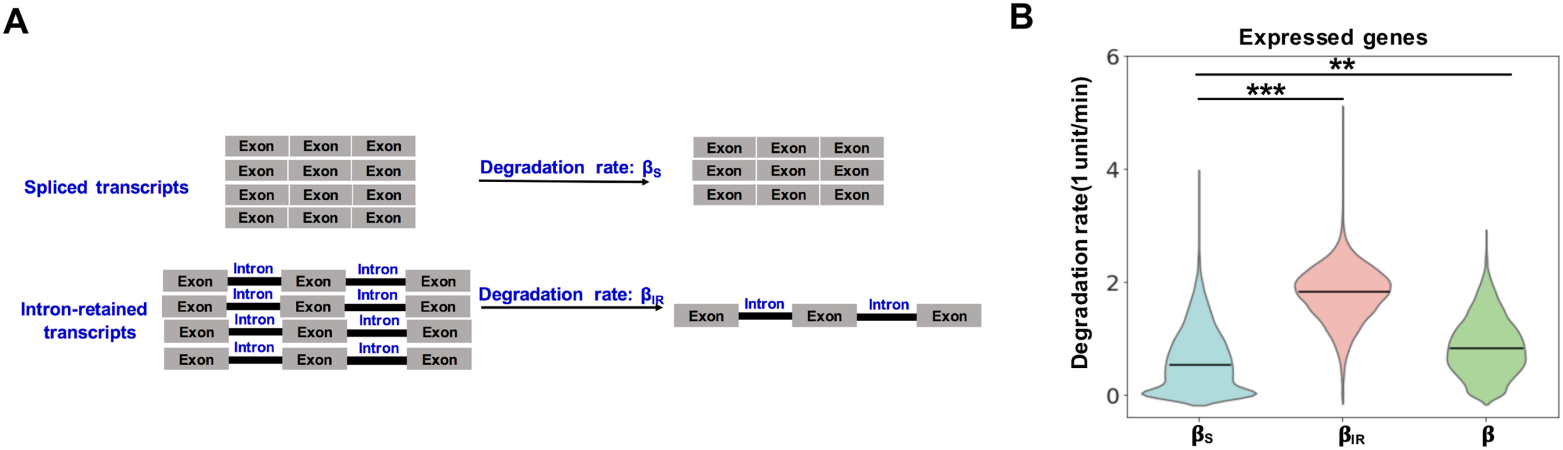
Intron-retained transcripts were significantly less stable than spliced transcripts. (**A**) Schematic diagram of the mixture model. The model considered intron-retained and spliced transcripts separately, each with its respective degradation rate (*β*_IR_ and *β*_S_). (**B**) Box plot showing the distribution of β_S_ and β_IR_ (fitted by the two-component model), *β* (fitted by the conventional one-component model) of expressed genes derived from BruChase-seq data of total RNA in resting CD4^+^T cells. In comparison, *β*_S_ was significantly different from *β*_IR_ and *β*, respectively. ** stands for *P* = 8.59 E–59 and *** stands for *P* < 1E–300 (one-sided Mann–Whitney *U* test).

We aimed to computationally determine the stability of intron-retained and spliced transcripts by comparing the BrU-labeled RNA from total RNA between 0.5 and 2 h time points. A set of nine housekeeping genes were used to normalize variations in RNA purification and BrU immunoprecipitation among different samples. These nine genes were chosen because their transcripts were stable in both resting and activated CD4^+^ T cells (see Materials and Methods for details on gene selection and normalization, Supplementary Figure S1A). Conventional modeling methods treated all transcripts of a given locus as a single RNA pool without considering the contribution of IR transcripts (4,6,21). We reasoned that discerning spliced and intron-retained transcripts would allow for a more accurate estimation of the degradation rate of spliced transcripts, especially for those transcribed loci with high IR. We therefore devised a first-degree mixture model that directly connected the mRNA expression and IR level at each chase time point, using two separate degradation rates (*β*_S_ for spliced transcripts and *β*_IR_ for IR transcripts), to fit the BruChase-Seq data (Figure 2A; see Materials and Methods for details). The modeling results were highly reproducible among three independent BruChase-Seq data sets of resting CD4^+^T cells, which were generated from unrelated blood donors (Supplementary Figure S3).

Overall, our data showed that spliced transcripts were significantly more stable than intron-retained transcripts (*P* < 1E-300, one-sided Mann–Whitney *U* test; Figure 2B). Interestingly, we noticed that our modeling results significantly differed from the degradation rates fitted by a conventional one-component model, β (Figure 2B, *P* = 8.59E–56, one-sided Mann–Whitney *U* test), consistent with the notion that intron-retained transcripts skew the estimation of the true degradation rate of spliced transcripts.

We next examined genes with extreme *β*_S_ values in resting and activated CD4^+^ T cells. We found 229 genes with high turnover rate (β_S_-high) in both resting and activated states. Similarly, 196 genes were identified with long mRNA half-lives in both states (*β*_S_-low; see Materials and Methods for details). Comparison of these two gene sets showed that the *β*_S_-low group was enriched in house-keeping genes (e.g. *ACTB* and *GAPDH*) whereas the β_S_-high group was enriched in transcriptional factors (*P* < 6.5E–32, Fisher's exact test, Supplementary Figure S4). Interestingly, the short-lived gene group included several Krüppel-like transcription factors (*KLF3, KLF6, KLF9, KLF11*) and zinc finger transcriptional factors involved in lymphocyte homeostasis (41) and viral infection (KEGG pathway, https://www.genome.jp/kegg/). Another example was *PRDM1* (the transcription factor *positive regulatory domain 1*), a divergent regulator of cell density and effector function for T cells (42).

### Corroboration of the two-component decay model by junction reads and cytoplasmic BruChase-Seq

**Figure 3.**
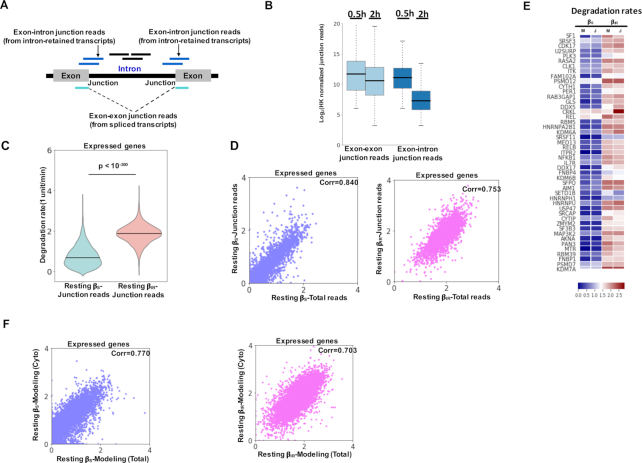
Validation of the mixture model using orthogonal approaches in resting CD4^+^ T cells. (**A**) Schematic diagram for using exon–exon and exon–intron junction reads to estimate β_S_ and β_IR_, respectively. (**B**) Distributions of normalized exon-exon and exon–intron junction reads of individual genes at the 0.5 and 2 h chase-time points based on the BruChase-seq data of total RNA of resting CD4^+^ T cells. (**C**) Violin plots showing the distribution of *β*_S_ and *β*_IR_ of expressed genes derived from the mixture model using junction reads in the resting state (*P* < 1E–300, one-sided Mann–Whitney *U* test). (**D**) Scatter plots showing the correlation between degradation rates of expressed genes derived from the mixture model using all reads (x-axis) and junction reads (y-axis) of total RNA BruChase-seq. *β*_S_: blue and *β*_IR_: pink. (**E**) Heat map for the β_S_ and β_IR_ values of 50 representative genes derived from the mixture model based on all reads (M) and the mixture model based on junction reads (J). The color denotes the row-wise *z*-score. (**F**) Scatter plots showing the correlation between degradation rates of expressed genes derived from BruChase-Seq using total (x-axis) and cytoplasmic (y-axis) RNA. *β*_S_: blue; *β*_IR_: pink.

We next explored multiple orthogonal approaches to ascertain the above modeling results. First, the degradation rates of spliced and intron-retained transcripts were computed based on exon-exon and exon-intron junction reads at the different chase times (Figure 3A, see Materials and Methods for detail) in the resting state. The normalized junction reads exhibited a clear difference in stability, with a higher decay rate for intron-containing transcripts than for spliced transcripts (*P* < 1E–300, one-sided Mann–Whitney *U* test, Figure 3B and C). On a global scale, the decay rates for individual genes estimated by two distinct methods (all reads versus junction reads) agreed well (*r* = 0.840 for *β*_S_, and *r* = 0.753 for *β*_IR_, Figure 3D and E).

We also performed BruChase-Seq analysis on cytoplasmic RNA prepared from pulse-labeled resting and activated CD4^+^ cells. RT-qPCR analysis of U6 RNA showed that nuclear contamination of cytoplasmic RNA fraction was negligible (Supplementary Figure S5). Notably, the decay rates derived from total and cytoplasmic RNA assessed by BruChase-Seq analysis were highly correlated: *r* = 0.770 for *β*_S_; *r* = 0.703 for *β*_IR_ (Figure 3F). In addition, consistent conclusions were obtained using cytoplasmic BruChase-seq data including the differential degradation rates between the spliced and intron-retained transcripts (Supplementary Figure S6A) and orthogonal corroboration of the two-component decay model by junction reads (Supplementary Figure S6B–E). By eliminating the contribution from nuclear RNA processing, cytoplasmic BruChase-seq is expected to provide a cleaner estimation of the degradation rate of spliced transcripts (*β*_S_). Because the reminder of the manuscript was focused on *β*_S_, we decided to use cytoplasmic BruChase-Seq data for the ensuing degradation analysis with total RNA BruChase-Seq data as additional supports.

### Global transcript stabilization upon T cell activation

**Figure 4.**
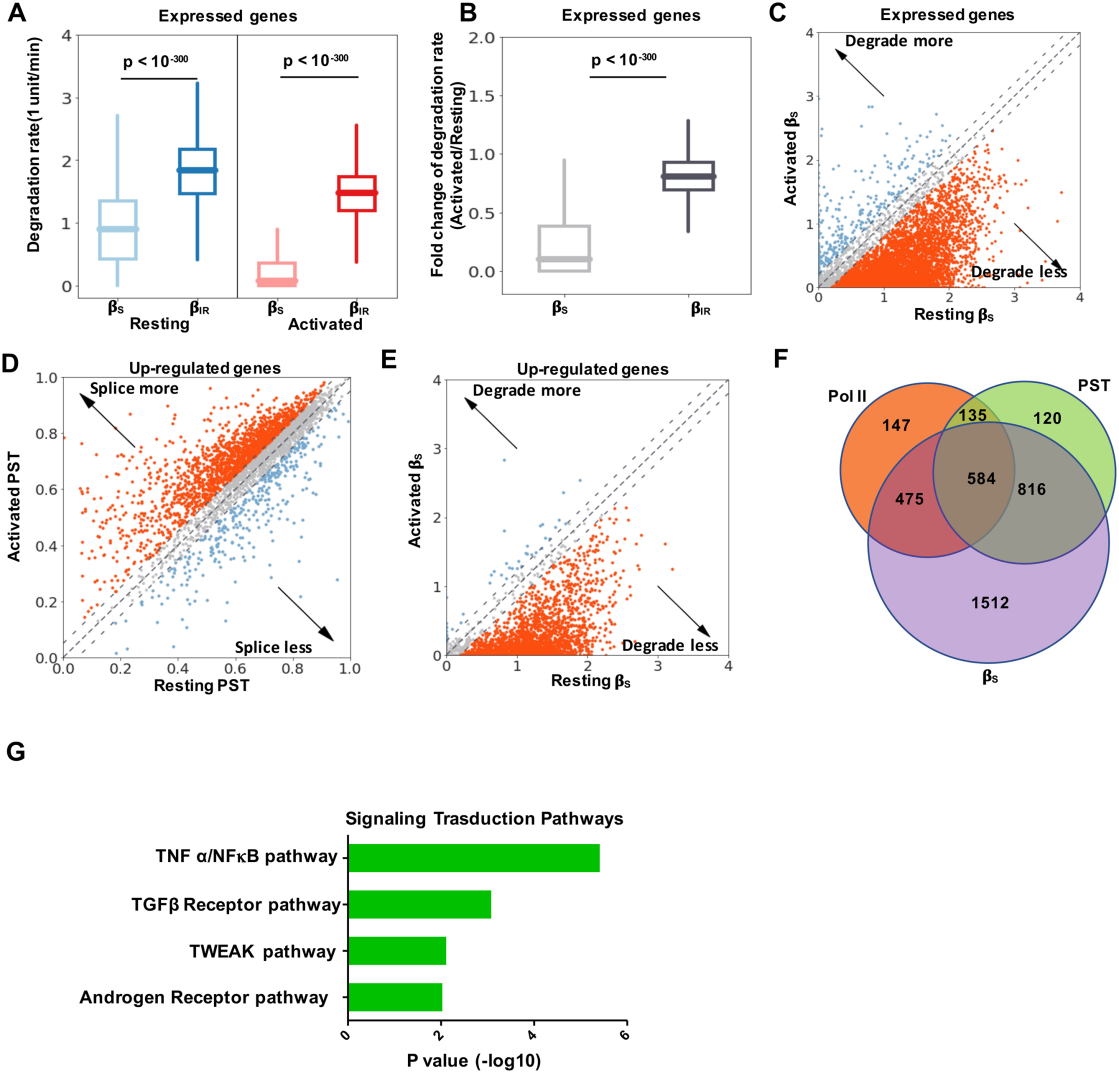
Coordinated regulation of gene expression upon T cell activation. (**A**) Box plot showing the distribution of the *β*_S_ and *β*_IR_ values of expressed genes derived from cytoplasmic BruChase-seq data in the resting and activated CD4^+^ T cells (*P* < 1E–300, one-sided Mann–Whitney *U* test). (**B**) Box plot showing the fold change in the *β*_S_ and *β*_IR_ values derived from cytoplasmic BruChase-seq data between the resting and activated states among expressed genes (*P* < 1E–300, one-sided Mann–Whitney *U* test). (**C**) Scatter plot showing the *β*_S_ values of expressed genes derived from cytoplasmic BruChase-seq data in the resting and activated states. Dashed lines denote the β_S_ difference = ± 0.2 (*P* < 1E–300, one-sided Mann–Whitney *U* test). (**D**) Scatter plot for the Percentage Spliced Transcript (PST) of the upregulated genes derived from 0.5 h BruChase-seq data of total RNA in the resting and activated states. Dashed lines denote the PST difference = ± 0.05 (*P* = 7.73E–50, one-sided Mann–Whitney *U* test). Up-regulated genes were determined from bulk RNA-seq data sets (see Materials and Methods for details) (18). (**E**) Scatter plot showing the β_S_ values of the upregulated genes derived from cytoplasmic BruChase-seq data in resting and activated states. Dashed lines denote the β_S_ difference = ± 0.2 (*P* < 1E–300, one-sided Mann–Whitney *U* test). (**F**) Venn diagram showing the overlap among three different mechanisms contributing to gene upregulation: transcriptional activation (RNA Pol II), increased splicing efficiency (PST), and increased mRNA stability (*β*_S_). (**G**) Signal transduction pathway analysis of the 1512 upregulated genes whose expression was predominately regulated via mRNA stability.

We next set out to identify transcripts with altered stability in resting and activated CD4^+^ T cells with cytoplasmic BruChase-seq data. By examining the expression of total transcripts over chase-time in resting and activated cells, we found that the overall degradation rate was significantly decreased upon activation (Supplementary Figure S7A). We then employed the mixture model to determine if spliced or intron-retained transcripts were the main contributor of increased transcript stability. Under both conditions, intron-retained transcripts were rapidly degraded with comparable degradation rates (*P* < 1E–300, one-sided Mann–Whitney *U* test; Figure 4A and B). However, fully spliced transcripts were more stable in activated CD4^+^ T cells than resting cells (Figure 4A and B). Using absolute difference of *β*_S_ ≥ 0.2 as a cutoff, we found that substantially more transcripts were stabilized rather than destabilized (*P* < 1E–300, one-sided Mann–Whitney *U* test; Figure 4C). The degradation rates derived above were also ascertained by orthogonal approaches using junction reads (Supplementary Figure S7B and C) and total RNAs (Supplementary Figure S7D).

We then focused on the 3944 genes upregulated upon T cell activation, which contribute to cell proliferation and cytokine secretion. Multiple mechanisms might be involved in elevated gene expression, including transcriptional activation, reduced IR and increased mRNA stability (18). Indeed, transcriptional activity, as measured by RNA Pol II chromatin immunoprecipitation-sequencing (ChIP-Seq), showed skewed distributions and tended to be increased in activated CD4^+^ T cells (*P* = 3.27E–15, One-sided Mann–Whitney *U* test; Supplementary Figure S8A). The observation was further confirmed by measuring the nascent transcripts using Bru-Seq data derived from total RNA (Supplementary Figure S8B and C). Splicing efficiency, which was an indicator of IR and approximated by the percentage of spliced transcripts (PST) derived from 0.5h-BruChase-seq data of total RNA, exhibited a skewed distribution towards the activated state (*P* = 7.73E–50, one-sided Mann–Whitney *U* test; Figure 4D). Finally, we also found that substantially more transcripts were stabilized rather than destabilized (*P* < 1E–300, one-sided Mann–Whitney *U* test; Figure 4E).

We then grouped the 3944 activation-induced genes into three major categories (see Materials and Methods for details): 1341 with increased Pol II occupancy, 1655 with increased splicing efficiency (PST) and 3387 with increased stability (βs). Considerable overlaps were observed among them (Figure 4F), suggesting potential coordination of underlying regulatory mechanisms. A complete list of all of the genes in these categories was provided in Supplementary Table S6. One sub-category was particularly interesting, comprising 1512 genes whose expression was predominately regulated at the RNA stability level (Figure 4F). GO analysis showed that the TNFα/NFκB pathway was significantly enriched in this group (Figure 4G). One prominent example was *NFκB1* mRNA, encoding a crucial transcriptional regulator. It works either as homodimer or heterodimer with RelA, which regulates the expression of a plethora of immunomodulatory factors, including cytokines, chemokines, adhesion molecules, antimicrobial factors, cell cycle regulators and cell survival factors (43).

### T cell activation stabilized *NFκB1* and *CRKL* transcripts

**Figure 5.**
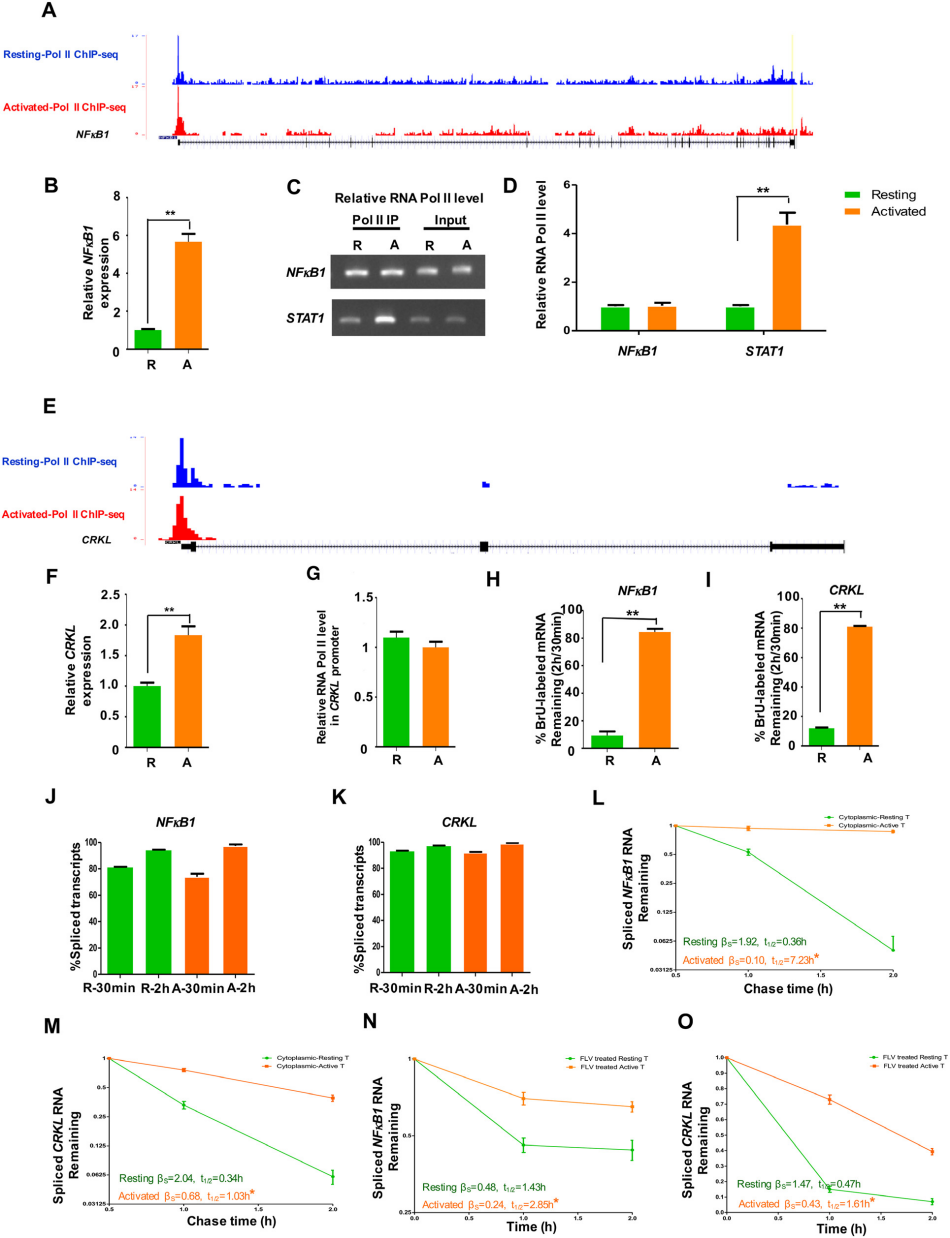
Activation-mediated stabilization of the *NFκB1* and *CRKL* mRNAs in human CD4^+^ T cell. (**A**) RNA Pol II (Pol II) ChIP-Seq tracks at the *NFκB1* locus. RNA Pol II ChIP-Seq data sets were downloaded from (18). (**B**) RT-qPCR analysis of the steady-state mRNA level of the *NFκB1* gene in the resting and activated CD4^+^ T cells. *GAPDH* was used as the internal control. The primer pairs were specific for spliced transcripts. (**C**, **D**) ChIP-PCR (C) and ChIP-qPCR (D) analysis of RNA Pol II occupancy at the promoter region of the *NFκB1 or STAT3* gene between the resting and activated CD4^+^ T cells, respectively. (**E**) RNA Pol II ChIP-Seq tracks at the *CRKL* locus. (**F**) RT-qPCR analysis of the steady-state *CRKL* mRNA level in the resting and activated CD4^+^ T cells. *GAPDH* was used as the internal control. (**G**) ChIP-qPCR analysis of RNA Pol II occupancy at the promoter region of the *CRKL* gene. (**H**, **I**) The stability of nascent mRNA of the *NFκB1* (H) or *CRKL* (I) gene before and after T cell activation. Using total RNA as the input material, the stability was assessed by the percentage of BrU-labeled spliced transcripts at 2 h to that at 0.5 h with RT-qPCR assay. *GAPDH* was used the internal controls. (**J**, **K**) The PST for the nascent mRNA of the *NFκB1* (J) or *CRKL* (K) gene before and after T cell activation. Using BrU-labeled RNA from total RNA preparation, the PST was determined by the fraction of the spliced transcripts in each transcribed locus of interest with RT-qPCR assay. (**L**, **M**) The stability of BrU-labeled cytoplasmic *NFκB1* (L) or *CRKL* (M) RNA was determined by RT-qPCR using a 3-point time course. Data from the resting and activated T cells are shown in green and orange, respectively. The mRNA half-life was calculated using a one-phase decay model. *GAPDH* was the internal controls. The significance of the difference was calculated using a paired t-test. (**N-O**) The stability of *NFκB1* (N) or *CRKL* (O) RNA was determined by RT-qPCR using a label-free approach with a 3-point time course (0, 1, 2 h after FLV treatment). *GAPDH* was the internal controls.R and A represent resting and activated CD4^+^ T cells, respectively. All error bars represent ± SEM from three biological replicates (*P < 0.05; **P < 0.01).

It is well established that NFκB signaling is tightly controlled by posttranslational modification, subcellular compartmentalization and interactions with other cofactors (44). However, stabilization of *NFκB1* transcripts upon T cell activation has not been investigated to the best of our knowledge. Our previous RNA-Seq study showed that the *NFκB1* mRNA level increased upon T cell activation. On the other hand, RNA Pol II ChIP-Seq (Figure 5A) (18) and Bru-seq result (Supplementary Figure S9A) showed that the transcriptional level of *NFκB1* gene remained relatively constant. The findings were confirmed by reverse transcription followed by quantitative PCR (RT-qPCR) (Figure 5B) and ChIP-qPCR (Figure 5C and D) assays, agreeing with the notion that the *NFκB1* mRNA level was largely regulated by transcript stability. Another example of stability-regulated genes was the *CRK-like proto-oncogene adaptor protein* (*CRKL*) (Figure 5E–G and Supplementary Figure S9B). It is a component of the CRKL–C3G complex which is required for a TCR-coupled signaling pathway and promotes integrin-dependent T-cell adhesion and migration (45). The stability of *NFκB1* and *CRKL* transcripts was further validated by metabolic labeling and RT-qPCR analysis before and after activation (Figure 5H and I). It was worth noting there was no significant change in the splicing efficiency of these two genes upon cell activation (Figure 5J and K), consistent with the genome-wide results.

Two approaches were employed to validate the degradation rate of candidate genes of interests, including pulse labelled cytoplasmic RNA (Figure 5L and M) and transcription inhibition with the CDK9 inhibitor Flavopiridol (FLV) (Figure 5N and O). Overall, both methods showed a consistent trend of increased stability in activated T cells for *NFκB1* and *CRKL*, as well as several additional activation-stabilized genes (Supplementary Figure S10). Of note, the exact half-lives inferred by these two methods differed significantly, likely due to the different methods employed. Taken together, the above results demonstrated that the regulation of *NFκB1* and *CRKL* gene expression was predominantly regulated at the RNA stability level.

### LARP4 was identified as a putative regulator involved in mRNA stabilization

**Figure 6.**
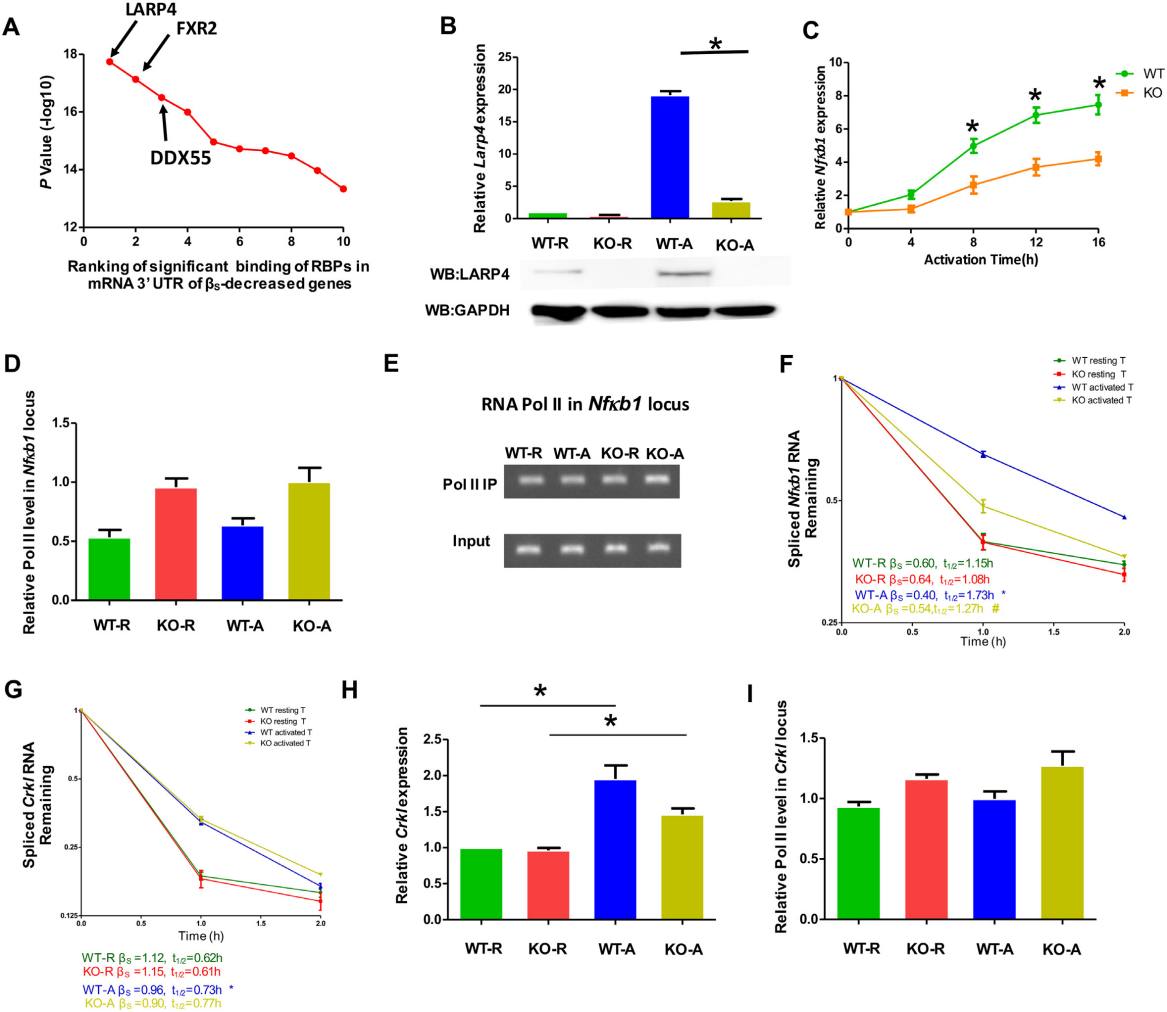
LARP4 was required for *Nfκb1* mRNA stabilization upon T cell activation. (**A**) Identification of candidate RBPs potentially involved in mRNA stability (see Method for details). The top 10 candidate RBPs along with their significance are shown. (**B**) RT-qPCR analysis of the relative steady-state mRNA (upper panel) and protein (lower panel) level of *Larp4* in resting and activated CD4^+^ T cells derived from WT and *Larp4* knockout (KO) mice. *Gapdh* mRNA and GAPDH protein was the internal controls of RT-qPCR and Western blotting analysis, respectively. (**C**) Mouse CD4^+^ T cells were activated with anti-CD3/CD28 antibodies at different time points as indicated. The mRNA expression level of *Nfκb1* was determined by RT-qPCR using *Gapdh* as the normalization control. Data from the WT and LARP4-KO mice are shown in green and orange, respectively. (**D**, **E**) ChIP-qPCR and ChIP-PCR analysis of RNA Pol II occupancy at the promoter region of the *Nfκb1* gene in resting and activated CD4^+^ T cells derived from WT and *Larp4* KO mice, respectively. (**F**, **G**) The degradation rate of *Nfκb1* (F) or *Crkl* (G) mRNAs in resting and activated CD4^+^ T cells was monitored by RT-qPCR following FLV treatment. The mRNA half-life was calculated using a one-phase decay model, and the significance of the difference was calculated using a paired *t*-test. * stands for *P* < 0.05 (WT resting CD4^+^ T cells versus WT activated CD4^+^ T cells) and # stands for *P* < 0.05 (WT activated CD4^+^ T cells versus KO activated CD4^+^ T cells). *Gapdh* served as the internal control. (**H**) The relative expression level of steady-state *Crkl* mRNA in resting and activated CD4^+^ T cells derived from WT and *Larp4* KO mice. *Gapdh* was used as the internal control. (**I**) ChIP-qPCR analysis of Pol II occupancy at the promoter region of the mouse *Crkl* gene in resting and activated CD4^+^ T cells derived from WT and *Larp4* KO mice. R and A represent resting and activated CD4^+^ T cells, respectively. All error bars represent ±SEM from three biological replicates (*P < 0.05).

We next sought to understand the basis of T cell activation-mediated mRNA stabilization. It is conceivable that RNA-binding proteins (RBPs) might contribute to mRNA stabilization/destabilization upon T cell activation (46). To this end, we took advantage of the enhanced cross-linking immunoprecipitation (eCLIP) data from the ENCODE project (https://www.encodeproject.org/) (47,48) and ranked RBPs according to their differential binding to the 3′ UTRs of upregulated genes with differential stability changes (see Materials and Methods for details). LARP4 was identified as the top candidate with a stabilizing effect (Figure 6A). Intriguingly, previous studies have established that LARP4 can promote the stabilization of a diverse set of mRNAs (30,49,50).

### LARP4 was induced upon CD4^+^ T cell activation and stabilized key functional mRNAs

To characterize the role of LARP4 in mRNA stability, we analyzed mice carrying a homozygous knockout allele of *Larp4* (KO) (30). For CD4^+^ T cells derived from wild-type (WT) mice, we found that the LARP4 mRNA and protein levels increased dramatically upon cell activation (Figure 6B). The *Nfκb1* mRNA level in WT CD4^+^ T cells also gradually increased during T cell activation, although the levels were significantly diminished in *Larp4 KO* CD4^+^ T cells at each time point (Figure 6C), consistent with LARP4 as a *Nfκb1* mRNA stabilization factor. We also validated the effect of LARP4 on the expression of *NFκB1* mRNA in human CD4^+^ T cells and found that silencing *LARP4* reduced *NFκB1* mRNA induction (Supplementary Figure S11). RNA Pol II ChIP-qPCR revealed that the Pol II occupancy at the *Nfκb1* locus was higher in the T cells derived from *Larp4* KO mice than from WT mice (Figure 6D and E), providing further evidence that transcriptional activity could not account for the difference in mRNA levels. In terms of *Nfκb1* mRNA stability, the half-life of *Nfκb1* mRNAs in resting WT and KO cells was comparable, while it was significantly more stable in activated WT cells than in activated KO cells (Figure 6F, *t*_1/2_ = 1.27 h versus 1.73 h). Taken together, these results supported the notion of LARP4-dependent *Nfκb1* mRNA stabilization during T cell activation.

We next tested transcript specificity in LARP4-mediated mRNA stabilization. *Crkl* mRNA also showed increased stability in activated mouse T cells compared to resting mouse T cells; however, by comparing WT and KO cells, the stability of *Crkl* mRNA was not dependent on LARP4 in both resting and activated T cells (Figure 6G). In addition, the Pol II occupancies and steady-state mRNA levels were similar in WT and *Larp4* KO cells (Figure 6H and I). Together, these data demonstrated the LARP4-independent stabilization of *Crkl* mRNA, indicating potential involvement of other RBPs.

### *Larp4* KO compromised the expression of NFκB-targeted genes

**Figure 7.**
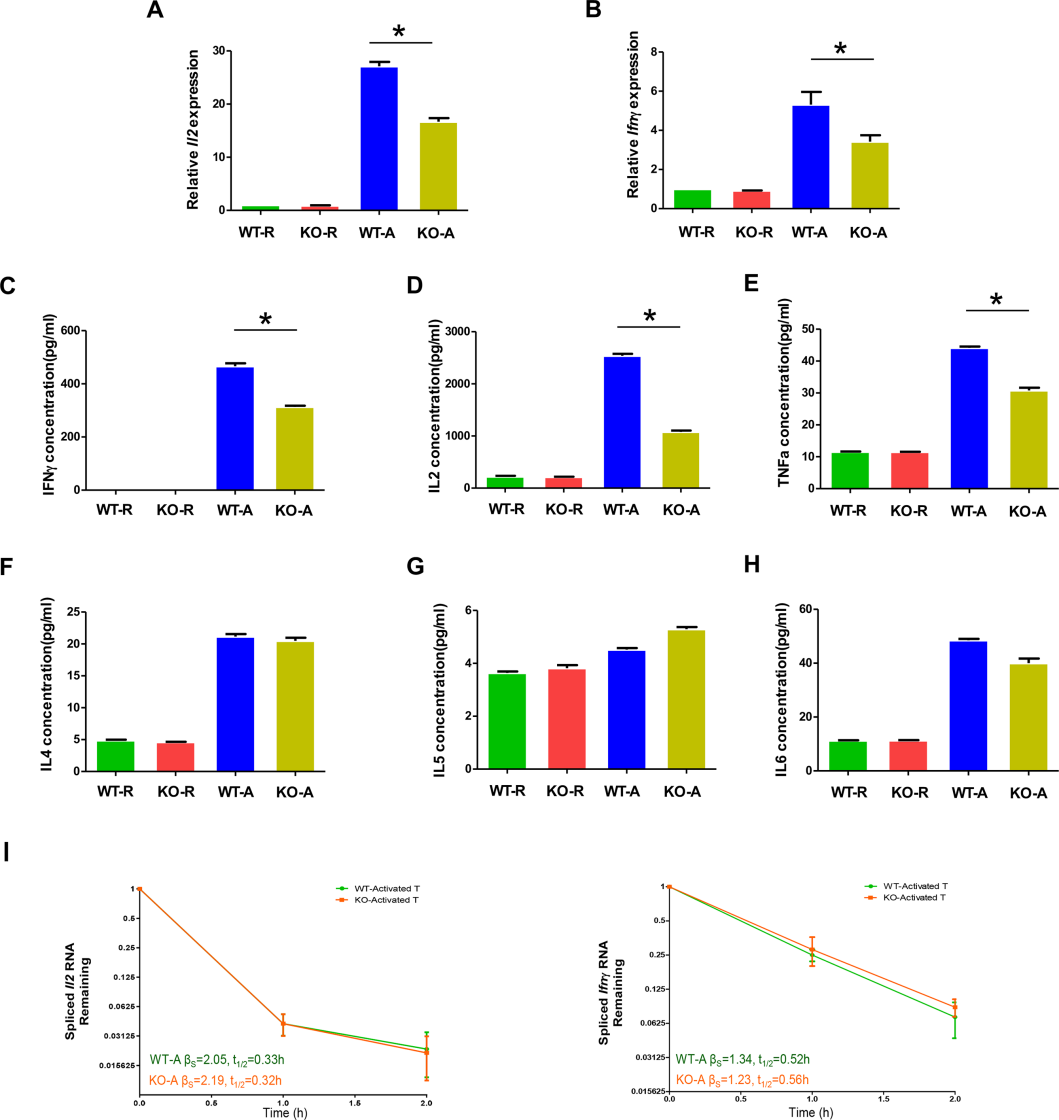
*Larp4* KO compromised the expression of NFκB target genes. (**A**, **B**) The relative mRNA levels of steady-state *Il2* and *Ifnγ* in resting and activated CD4^+^ T cells derived from the WT and *Larp4* KO mice. *Gapdh* was used the internal control. (**C–H**) Analysis of cytokine secreted by resting and activated CD4^+^ T cells derived from the WT and *Larp4* KO mice. The level of each cytokine in the culture supernatant was determined by the LEGENDplex™ Cytometric Bead Assay (BioLegend). The concentrations of IFNγ (C), IL2 (D), TNFα (E), IL4 (F), IL5 (G) and IL6 (H) were determined. (**I**) The stability of *Il2* and *Ifnγ* mRNA transcripts, as measured by RT-qPCR following FLV treatment for the indicated time, in activated CD4^+^ T cells obtained from WT and *Larp4* KO mice. *Gapdh* was used the internal controls.R and A represent resting and activated CD4^+^ T cells, respectively. The error bars represent ± SEM based on three biological replicates (*P < 0.05).

Activated CD4^+^ T cells secrete multiple cytokines critical for the proliferation and function of immune cells (2). Two well-established cytokines, IL2 and IFNγ, are under the direct control of NFκB at the transcriptional level (http://www.bu.edu/nf-kb/gene-resources/target-genes/). We therefore measured *Il2* and *Ifnγ* mRNAs in CD4^+^ T cells from WT and *Larp4* KO mice to assess the potential effects of *Larp4* knockout on T cell function. As expected, WT CD4^+^ T cell activation increased the steady-state level of both mRNAs (Figure 7A and B). Such increases were significantly reduced when *Larp4* was knocked out (Figure 7A and B). We also assayed for several cytokines produced by CD4^+^ T cells (Figure 7C–H and Supplementary Figure S12). The protein levels of IL2 and IFNγ in CD4^+^ T cells were significantly induced upon cell activation under both WT and *Larp4* KO conditions. However, the levels of both proteins were higher in WT cells than in KO cells, consistent with the observation obtained from the mRNA data (Figure 7C and D). *Il2 and Ifnγ* mRNA stability analysis showed a comparable degradation rate (Figure 7I), ruling out the direct effect of LARP4 on *Il2* and *Ifnγ* stability. These data supported the notion that the difference observed was likely due to changes in the NFκB expression level.

### Mechanistic investigation of LARP4-mediated *NFκB1* mRNA stability

**Figure 8.**
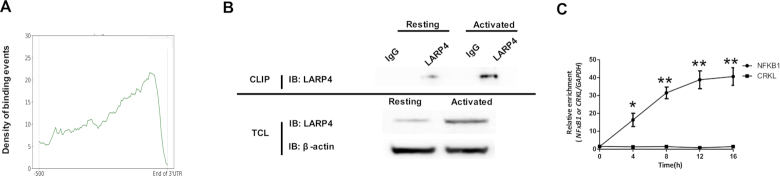
Recruitment of LARP4 to its cognate transcripts was mediated by the 3′ untranslated region. (**A**) LARP4–mRNA interaction events were enriched towards the distal end of the 3′ UTRs. The binding profile was derived from the ENCODE LARP4 eCLIP-Seq data from K562 cells. The y-axis represents the density of LARP4 binding events. (**B**, **C**) The relative enrichment of LARP4 at the 3′ UTR of the *NFκB1* and *CRKL* mRNAs. Human resting CD4^+^ T cells were activated with anti-CD3/CD28 antibodies for the time intervals as indicated. Cross-linking immunoprecipitation (CLIP) was performed using an anti-human LARP4 antibody or a nonspecific IgG. The resulting materials were immunoblotted (IB) with anti-LARP4 antibody (B, upper panel). The total cell lysates (TCL) from resting and activated human CD4^+^ T cells were immunoblotted with anti-LARP4 and anti-β-actin antibodies (B, lower panel). The resulting RNA was analyzed by RT-qPCR using specific primers against the 3′ UTRs of *NFκB1, CRKL* and *GAPDH*. The enrichment of LARP4 at the 3′ UTR of the *NFκB1* and *CRKL* mRNAs was determined using *GAPDH* as the internal control. Data are expressed as the fold change of relative enrichment between LARP4 and IgG immunoprecipitation. R and A represent resting and activated CD4^+^ T cells, respectively. All error bars represent ±SEM from three biological replicates (*P < 0.05; **P < 0.01).

To better understand how LARP4 functions in regulating mRNA stability, we took advantage of the LARP4 eCLIP data in the K562 cell line to identify potential binding preferences. Interestingly, we found that the LARP4 binding events were highly enriched towards the end of 3′ UTRs (Figure 8A). The protein level of LARP4 in human CD4^+^ T cells increased dramatically upon cell activation (Figure 8B). Cross-linking immunoprecipitation-qPCR (CLIP-qPCR) was then performed with primer pairs targeting the 3′ UTR of the *NFκB1 and CRKL* transcripts (Supplementary Figure S13). We found that LARP4 immunoprecipitation was specific (Figure 8B). The interaction between LARP4 with the *NFκB1* transcript did not occur in human resting CD4^+^ T cells but gradually increased during T cell activation (Figure 8C). The results agreed well with the observation that *Larp4* KO destabilized *Nfκb1* transcripts in only activated T cells (Figure 6F). Furthermore, no binding was detected between LARP4 and *CRKL* during T cell activation, consistent with LARP4-independent regulation of *CRKL* mRNA stability (Figure 8C). Taken together, these data suggested that the interaction between LARP4 and cognate mRNA might contribute to target specificity.

## DISCUSSION

In this study, we performed BruChase-Seq to monitor the expression dynamics of nascent transcripts in resting and activated CD4^+^ T cells. To our knowledge, this was the first genome-wide study to investigate RNA stability by considering both spliced and intron-retained transcripts. Using multiple orthogonal approaches our results confirmed that intron-retained transcripts were generally less stable than spliced transcripts, highlighting the coupling between IR and RNA stability as a novel mechanism to control gene expression.

We found spliced transcripts overall were stabilized upon T cell activation (Figure 4A–C), leading to increased gene expression. In addition, the vast majority of transcripts exhibit reduced IR, reinforcing the effect of activation-mediated mRNA stabilization. These asymmetric posttranscriptional gene regulation events likely reflected a consistent theme of T cell activation and drove polarized cell specifications.

Our data allowed for the integrative investigation of gene regulation in T cell activation, including transcription, splicing (IR) and mRNA stability. While extensive cross-talk do exist among different mechanisms, we focused on the 1512 genes whose expression was primarily regulated by RNA stability, for which the NFκB pathway was highly enriched. Previous studies on the regulation of NFκB1 activity have mainly focused on posttranslational mechanisms as well as subcellular localization (44). Here, we uncovered an additional layer of regulation via mRNA stability, highlighting its complexity and importance in adaptive immunity.

The stability of mRNA can be regulated by multiple mechanisms, including alternative 3′ UTR choice (46,51), regulation of the poly(A) tail length (52–55), and interactions between RBPs and their cognate sites (56,57). We provided multiple lines of evidence demonstrating LARP4 as a major contributor of *Nfκb1* stability. *Nfκb1* mRNA was stabilized upon activation of WT CD4^+^ T cells, and the stabilization was compromised by LARP4 ablation (Figure 6F). We found evidence from eCLIP ENCODE data of LARP4 binding sites in the 3′ UTRs of mRNAs in K562 cells (Figure 8A). Consistent with this we confirmed LARP4 binding to the 3′ UTR of *NFκB1* mRNA by eCLIP RT-qPCR (Figure 8C).

LARP4 is an intriguing protein. Previous studies have demonstrated that it harbors multiple interaction domains; it can bind polyA directly as well as the cytoplasmic poly(A)-binding protein, PABP (49,58). Its activity to protect poly(A) tail from shortening is linked to mRNA stability (30). LARP4 is found in large ribonucleoprotein complexes *in vivo*, on translating polyribosomes, where mRNA metabolism is active (59). Consistent with this, we found that the LARP4 binding sites were highly enriched towards the end of 3′ UTRs (Figure 8A). We also noted that the stability of *Nfκb1* transcripts was moderately increased by T cell activation in *Larp4* KO mice, suggesting that additional RBPs might also be involved. DDX6, a well-known RBP that promotes RNA degradation (60,61), was the third highest ranked candidate from our computational screen (Figure 6A). Interestingly, *DDX6* expression levels decreased upon T cell activation, consistent with the model of increasing mRNA half-life by downregulating a negative regulator of mRNA stability.

In this study, the importance of LARP4 in T cell function was demonstrated by the compromised expression level of IL2 and IFNγ, two well-known targets of NFκB, in activated CD4^+^ cells derived from *Larp4* KO mice. Future studies are warranted to evaluate posttranscriptional regulation, such as LARP4-mediated RNA stabilization, in maintaining a proper immune response. Moreover, this study served as a valuable resource for reliably evaluating mRNA stability in CD4^+^ T cells. The systems approach employed here might help pave the road to a more comprehensive understanding of T cell biology.

## DATA AVAILABILITY

The raw data from the Bru-Seq and BruChase-Seq analyses be found at the Gene Expression Omnibus with GEO number GSE113730. We also downloaded published datasets of RNA pol II ChIP-Seq and bulk RNA-Seq of human resting and activated CD4^+^ T cells at the NCBI Sequence Read Archive with SRA number SRP058500.

## Supplementary Material

gkaa643_Supplemental_File
