# Supplementary material for: Transcriptome-wide stability analysis uncovers LARP4-mediated NFκB1 mRNA stabilization during T cell activation
Source: Nucleic Acids Res. 2020 Jul 31;48(15):8724–39. doi: 10.1093/nar/gkaa643 (PMC7470963; doi:10.1093/nar/gkaa643)
Supplement: gkaa643_Supplemental_File [file gkaa643_supplemental_file.pdf]

# Transcriptome-wide stability analysis uncovers LARP4-mediated NFκB1 mRNA stabilization during T cell activation

Yi Tian[1,2,3,†], Zhouhao Zeng[1,†], Xiang Li[1,†], Yiyin Wang[2], Runsen Chen[2], Sandy Mattijssen[4], Sergei Gaidamakov[4], Yuzhang Wu[3], Richard Maraia[4], Weiqun Peng[1,*], Jun Zhu[2,*]

## Inventory

**Figure S1. The stability of BrU-labeled transcripts derived from 12 housekeeping genes in resting and activated CD4$^+$ T cells.**

**Figure S2. Enrichment analysis of BrU immunoprecipitation.**

**Figure S3. Comparison of $\beta_S$ and $\beta_{IR}$ of expressed genes derived from three independent datasets of human resting CD4$^+$ T cells.**

**Figure S4. Genes with unstable and stable transcripts were enriched in distinct functions.**

**Figure S5. RT-qPCR analysis of the relative expression level of U6 splicesomal RNA in cytoplasmic (Cyto) and nuclear (Nuc) RNA.**

**Figure S6. Validation of the mixture model using cytoplasmic BruChase-seq data of resting CD4$^+$ T cells.**

**Figure S7. Validation using orthogonal approaches.**

**Figure S8. Comparative analysis of transcriptional activity determined by RNA Pol II ChIP-Seq and Bru-Seq.**

**Figure S9. Normalized read-profiles (Bru-seq data) of BrU-labeled transcripts for the *NFκB1* (A) and *CRKL* (B) genes at 0h time point in resting and activated human CD4$^+$ T cells.**

**Figure S10. Validation of activation-stabilized transcripts using cytoplasmic mRNA and transcription inhibition.**

**Figure S11. The effect of *LARP4* knockdown (KD) on *NFκB1* expression in human CD4$^+$ T cells.**

**Figure S12. Cytokine production by WT and *Lapr4* KO CD4$^+$ T cells.**

**Figure S13. The coordinates of the CLIP-qPCR primer pairs in the 3'UTR of *NFkB1* and *CRKL.***

**Table S1. Primers used for RT-PCR validation of the spliced and intron-retained transcript level in human.**

**Table S2. Primers used for RT-PCR validation of the spliced transcript level in mouse.**

**Table S3. Primers used for Pol II ChIP-qPCR validation in human.**

**Table S4. Primers used for Pol II ChIP-qPCR validation in mouse.**

**Table S5. Primers used for LARP4 CLIP-qPCR validation in human.**

**Table S6. The complete list for the upregulated genes with increased Pol II occupancy, increased splicing efficiency (PST) and/or increased stability ($\beta_S$) upon activation in human T cells.**

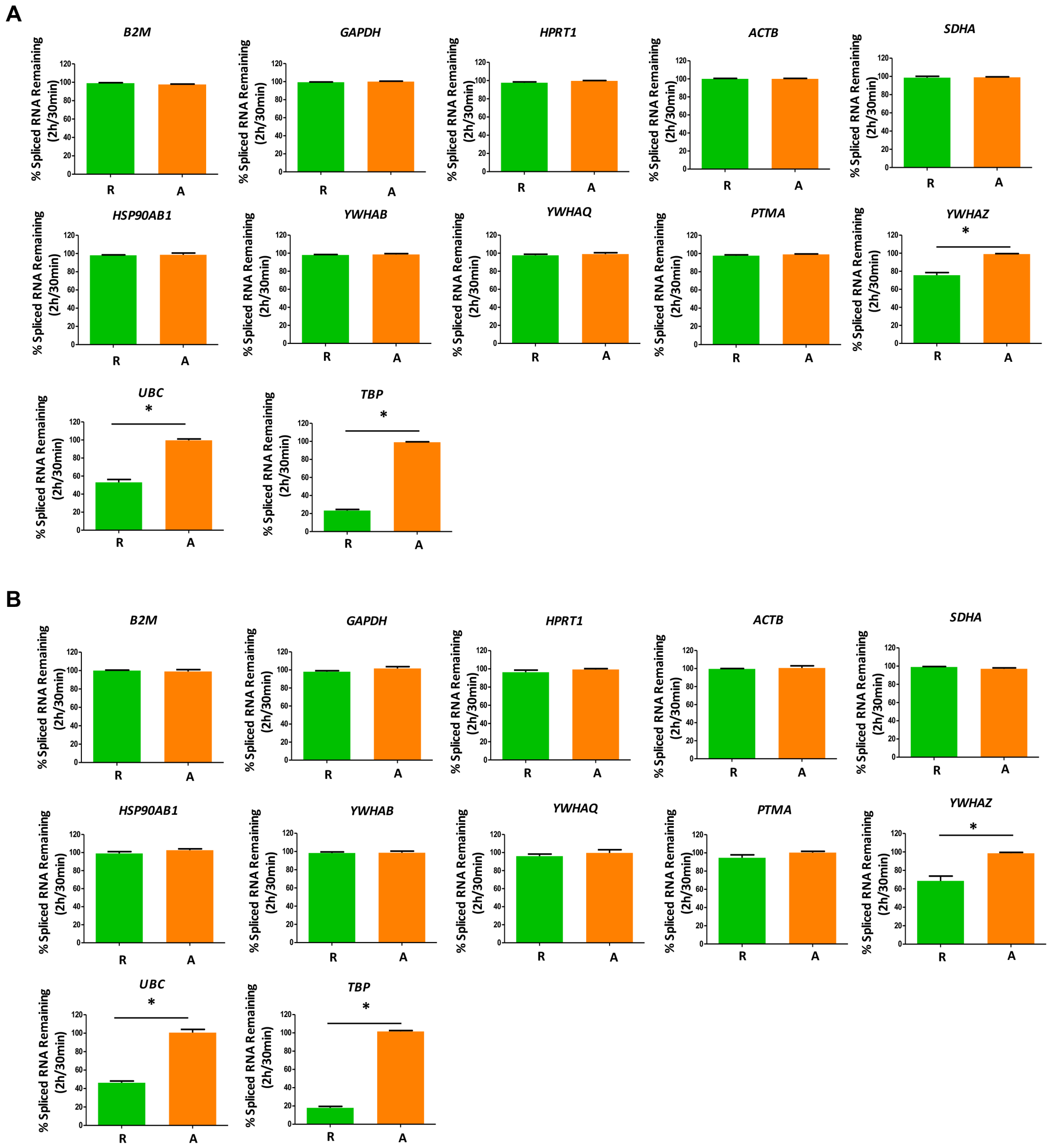


**Figure S1. The stability of BrU-labeled transcripts derived from 12 housekeeping genes in resting and activated CD4$^+$ T cells.**

The Spike-in control RNA (ERCC-00002) was BrU labelled by in vitro transcription and added into individual BruChase-seq samples before RNA purification at equal amount.

A. Using total RNA as the input material, the stability of each transcript of interest was assessed by the percentage of BrU-labeled spliced transcripts at 2 h to that at 0.5 h, normalized by the level of BrU-labeled spike-in control. B. Using cytoplasmic RNA as the input material, the stability of each transcript of interest was assessed by the percentage of BrU-labeled spliced transcripts at 2 h to that at 0.5 h, normalized by the level of BrU-labeled spike-in control. R and A represent resting and activated CD4$^+$ T cells, respectively. The error bars represent ±SEM based on three biological replicates (*$p<0.05$).

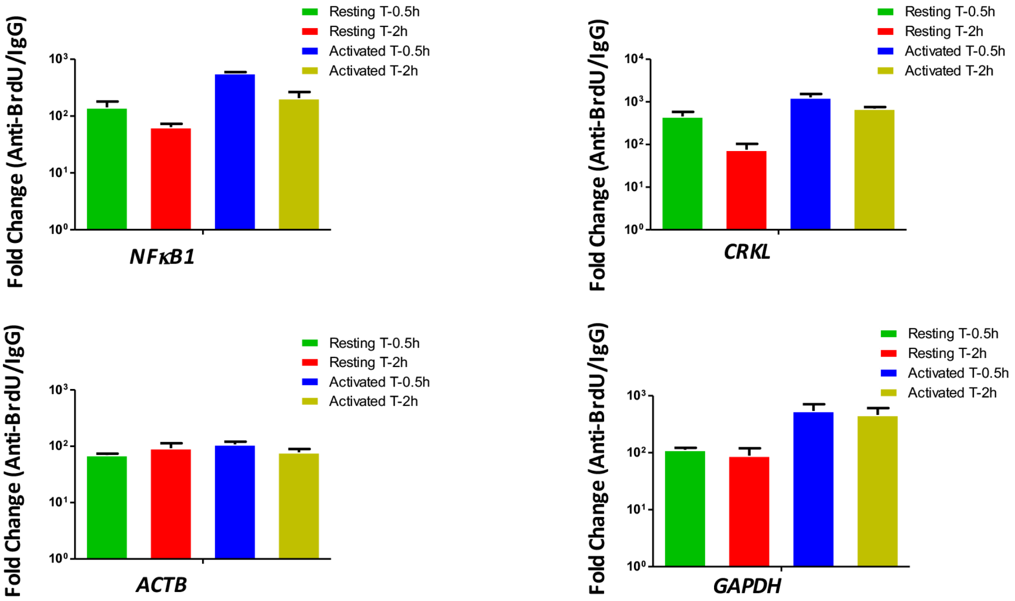


**Figure S2. Enrichment analysis of BrU immunoprecipitation.**

For each BruChase time point, BrU-labeled RNAs were immunoprecipitated with specific anti-BrdU antibody or non-specific IgG. The resulting RNAs were then subjected to RT-qPCR analysis. The fold enrichment of BrU RNA was determined by the ratio of the relative abundance of each transcript in specific and non-specific IP experiments. The error bars represent ±SEM based on three biological replicates.

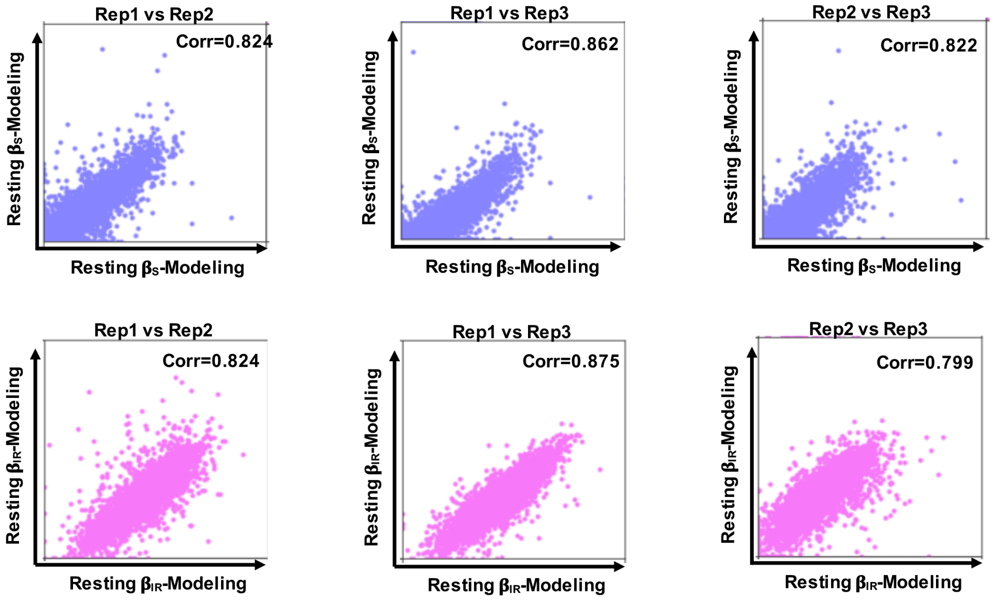


**Figure S3. Comparison of $\beta_S$ and $\beta_{IR}$ of expressed genes derived from three independent datasets of human resting CD4$^+$ T cells.**

Scatter plots showing pairwise correlation of model-fitted $\beta_S$ (blue) or $\beta_{IR}$ (pink) among three independent BruChase-Seq data sets. The three data sets were obtained using human resting CD4$^+$ T cells prepared from unrelated blood donors.

| | Homo Sapiens Housekeeping Genes 3804 | Homo Sapiens Transcription Factors Genes 1665 |
|---|---|---|
| $\beta_S$-low group: 196 genes from Bottom 1200 of βs, with RPKM > 30 | 113 | 10 |
| $\beta_S$-high group: 229 genes from Top 500 of βs | 44 | 98 |

Fisher Exact Test: p-value=6.4899e$^{-32}$

**Figure S4. Genes with unstable and stable transcripts were enriched in distinct functions.**

229 unstable transcripts were identified as the overlap between the top 500 transcripts with the highest $\beta_S$ in resting and activated states. 196 highly-expressed (RPKM>30 in both resting and activated states) stable transcripts were identified as those ranked among the bottom 1200 in terms of $\beta_S$ in both resting and activated states. The overlaps of these two gene sets with a database of housekeeping genes (https://www.tau.ac.il/~elieis/HKG/HK_genes.txt) and a database of transcription factors are shown (p =6.49E-32, Fisher's exact test).

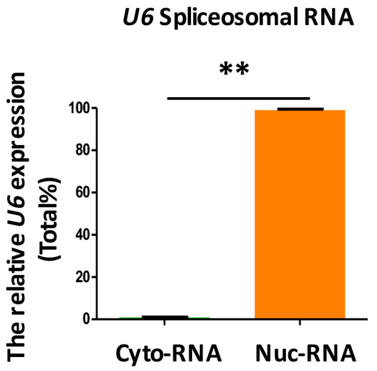


**Figure S5. RT-qPCR analysis of the relative expression level of U6 splicesomal RNA in cytoplasmic (Cyto) and nuclear (Nuc) RNA.**

The relative expression level of U6 splicesomal RNA in total, Cyto and Nuc RNA was determined by RT-qPCR. Data are expressed as the percentage of the total RNA. The error bars represent ±SEM based on three biological replicates (**p < 0.01).

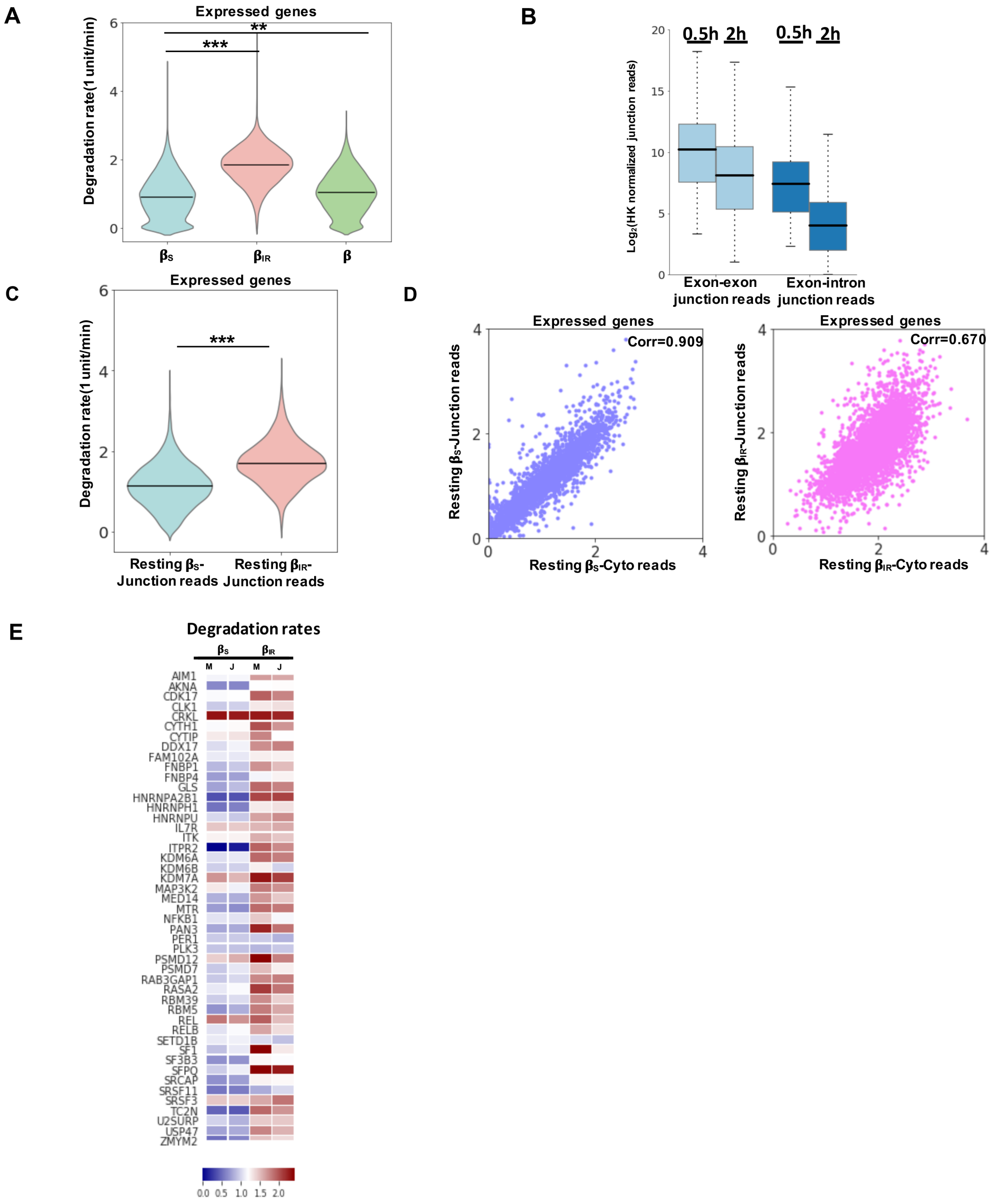


**Figure S6. Validation of the mixture model using cytoplasmic BruChase-seq data of resting CD4$^+$ T cells.**

**A.** Box plot showing the distribution of $\beta_S$ and $\beta_{IR}$ (fitted by the two-component model), β (fitted by the conventional one-component model) of expressed genes derived from cytoplasmic BruChase-seq data in resting CD4$^+$ T cells. In comparison, $\beta_S$ was significantly different from βIR and β, respectively. ** stands for p = 1.57E-46 and *** stands for p < 1E-300 (one-sided Mann-Whitney U test).

B. Distributions of normalized exon-exon and exon-intron junction reads of individual genes at the 0.5 h and 2 h chase-time points based on the cytoplasmic BruChase-seq data of resting CD4$^+$ T cells.

**C.** Violin plots showing the distribution of $\beta_S$ and $\beta_{IR}$ of expressed genes derived from the mixture model using junction reads in the resting state (p < 1E-300, one-sided Mann-Whitney U test).

**D.** Scatter plots showing the correlation between degradation rates of expressed genes derived from the mixture model using all reads (Cyto reads, x-axis) and junction reads (y-axis) of the cytoplasmic BruChase-seq data. $\beta_S$: blue and $\beta_{IR}$: pink.

**E.** Heat map for the $\beta_S$ and $\beta_{IR}$ values of 50 representative genes derived from the mixture model based on all reads (M) and the mixture model based on junction reads (J). The color denotes the row-wise z-score.

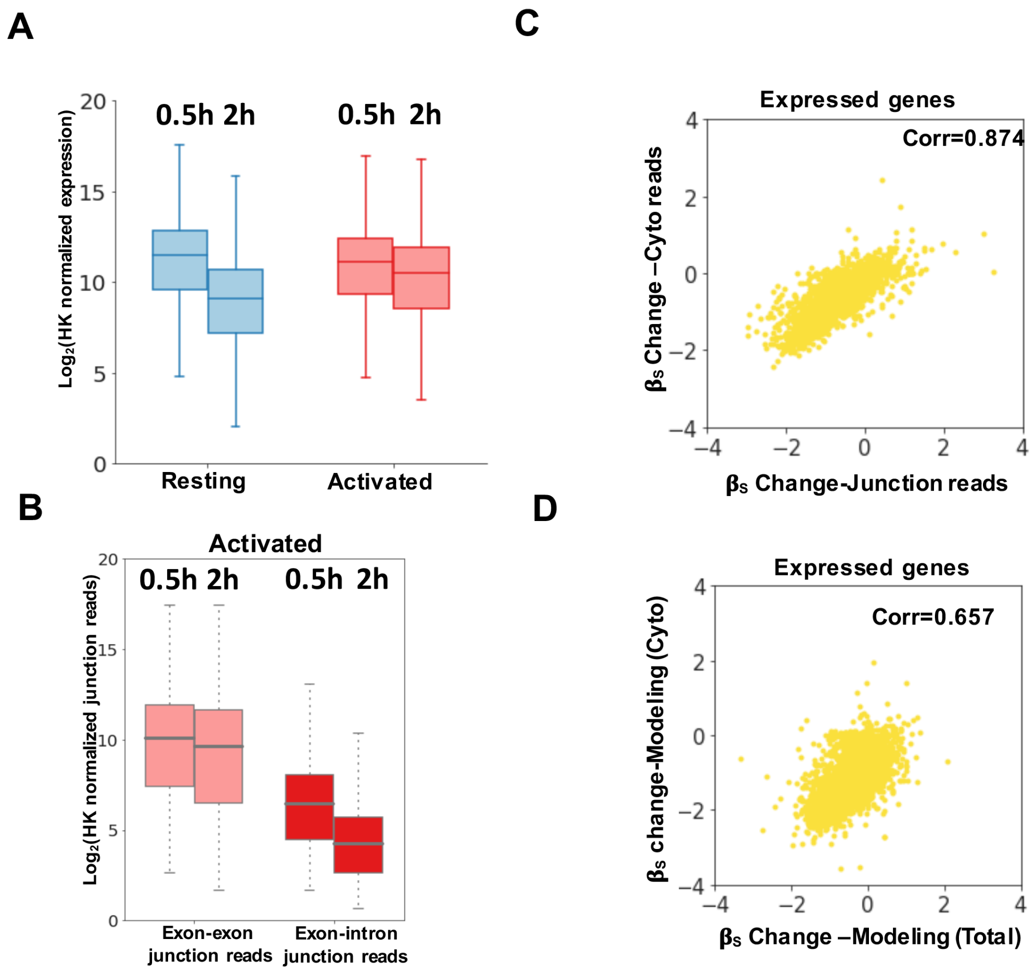


**Figure S7. Validation using orthogonal approaches.**

A. Boxplot showing the distribution of normalized gene expression levels at 0.5 h and 2 h of the cytoplasmic BruChase-seq data.

B. Boxplot showing normalized read counts of exon-exon junction reads and exon-intron junction reads at 0.5 h and 2 h of the cytoplasmic BruChase-seq data of activated $CD4^+$ T cells.

C. Correlation analysis of $\beta_S$ changes (activated-resting) estimated by the two-component mixture model using junction reads (x-axis) and all reads (Cyto reads, y-axis) of cytoplasmic BruChase-seq data.

D. Correlation analysis of $\beta_S$ changes (activated-resting) estimated by the two-component mixture model based on total (x-axis) and cytoplasmic (y-axis) BruChase-seq data.

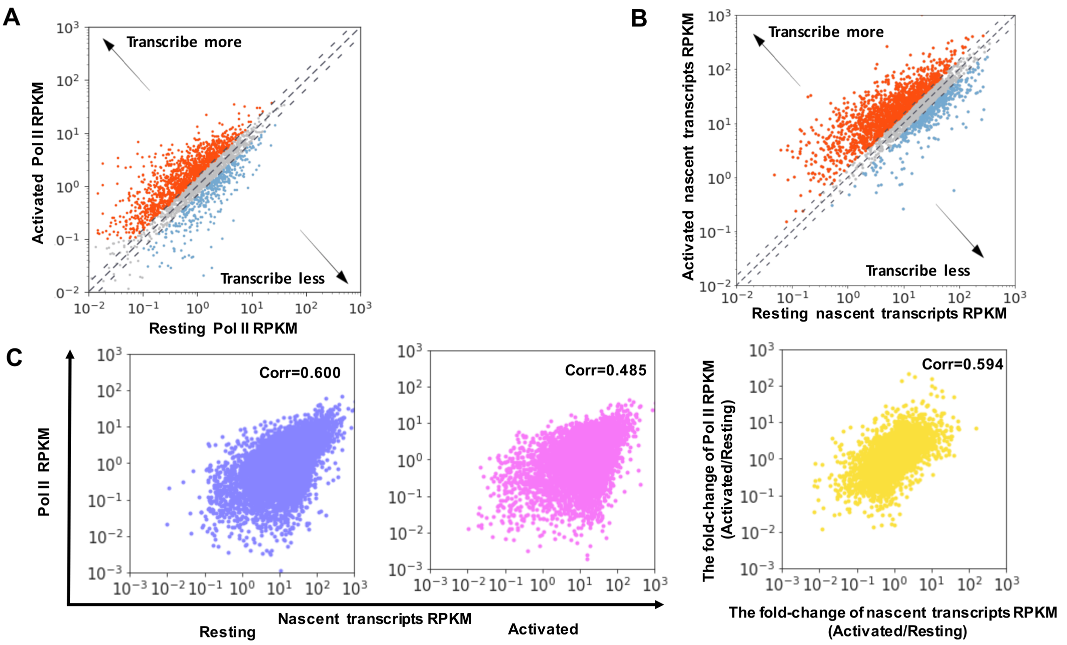


**Figure S8. Comparative analysis of transcriptional activity determined by RNA Pol II ChIP-Seq and Bru-Seq.**

A. Scatterplot showing the RPKM level of RNA Pol II at the promoters of the upregulated genes under resting (x-axis) and activated (y-axis) conditions. Dashed lines denote Pol II RPKM fold change = ± 1.5. p = 3.27E-15, one-sided Mann-Whitney U test.

B. Scatterplot showing the RPKM level of nascent transcripts (Bru-seq or BruChase-Seq 0 hr) of the upregulated genes under resting (x-axis) and activated (y-axis) conditions. Dashed lines denote Pol II RPKM fold change = ± 1.5. p = 3.77E-41, one-sided Mann-Whitney U test.

C. Scatterplot showing the correlation between the RNA Pol II RPKM (x-axis) and nascent transcripts RPKM (y-axis). The results for resting and activated CD4$^{+}$ T cells are shown in blue and pink, respectively. The changes between the two conditions (activated/resting) are shown in yellow.

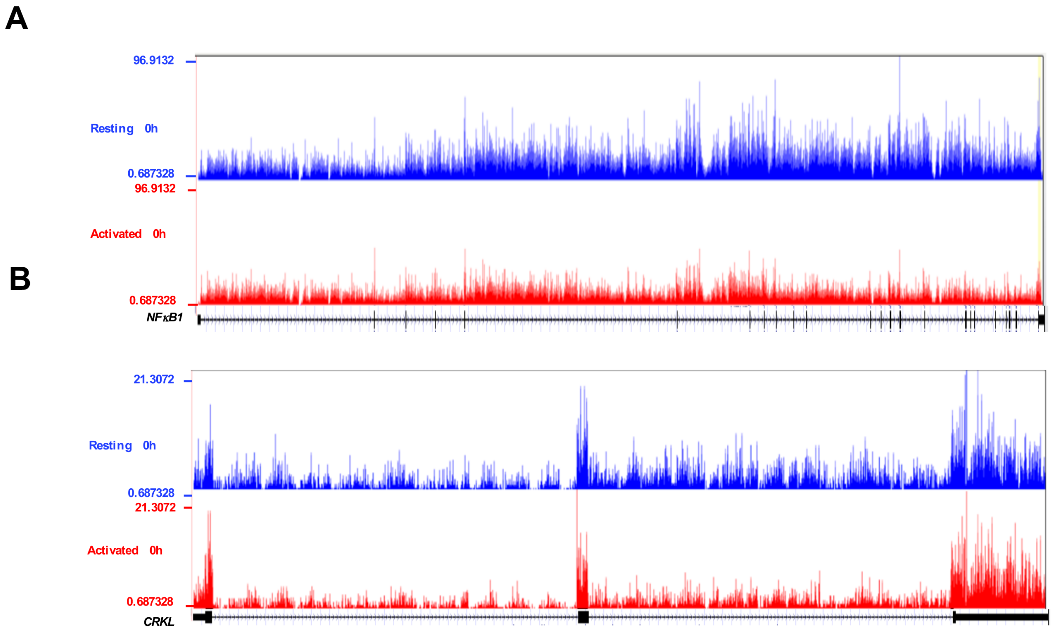

**Figure S9. Normalized read-profiles (Bru-seq data) of BrU-labeled transcripts for the *NFκB1* (A) and *CRKL* (B) genes at 0h time point in resting and activated human CD4$^+$ T cells.**

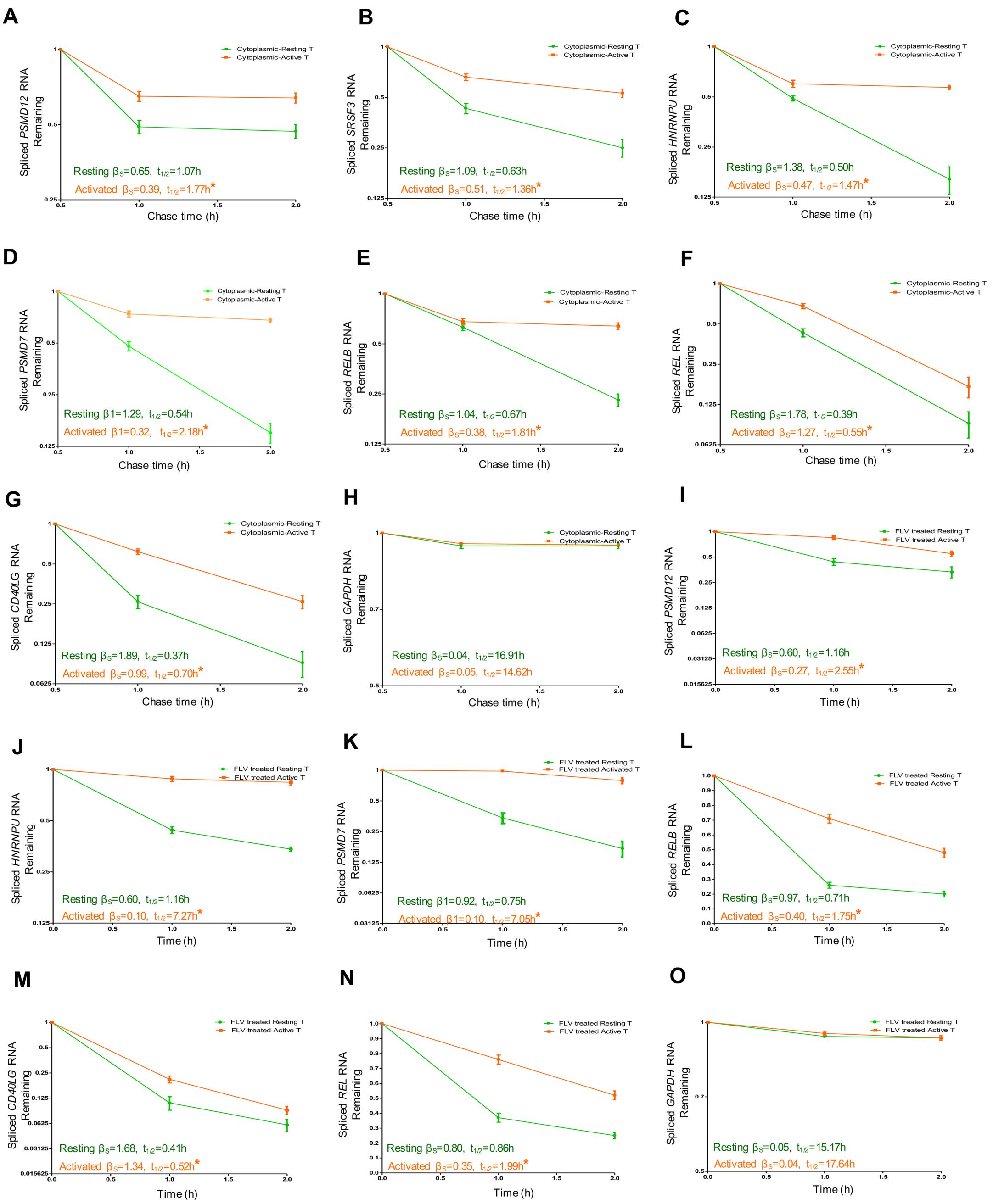


**Figure S10 Validation of activation-stabilized transcripts using cytoplasmic mRNA and transcription inhibition.**

The half-lives of BrU-labeled cytoplasmic *PSMD12* (A), *SRSF3* (B), *HNRNPU* (C), *PSMD7* (D), *RELB* (E), *REL* (F), and *CD40LG* (G) mRNAs were determined by RT-qPCR using a 3-point time course. The half-lives of *PSMD12* (I), *HNRNPU* (J), *PSMD7* (K), *RELB* (L), *CD40LG* (M), and *REL* (N) mRNAs were determined by RT-qPCR following FLV-mediated transcription inhibition. The *GAPDH* (H and O) transcript was used as a internal control. Data from the resting and activated T cells are shown in green and orange, respectively. The mRNA half-life was

calculated using a one-phase decay model. The significance of the differences was calculated using paired t-test and Error bars represent ±SEM from three biological replicates (* $p < 0.05$; ** $p < 0.01$).

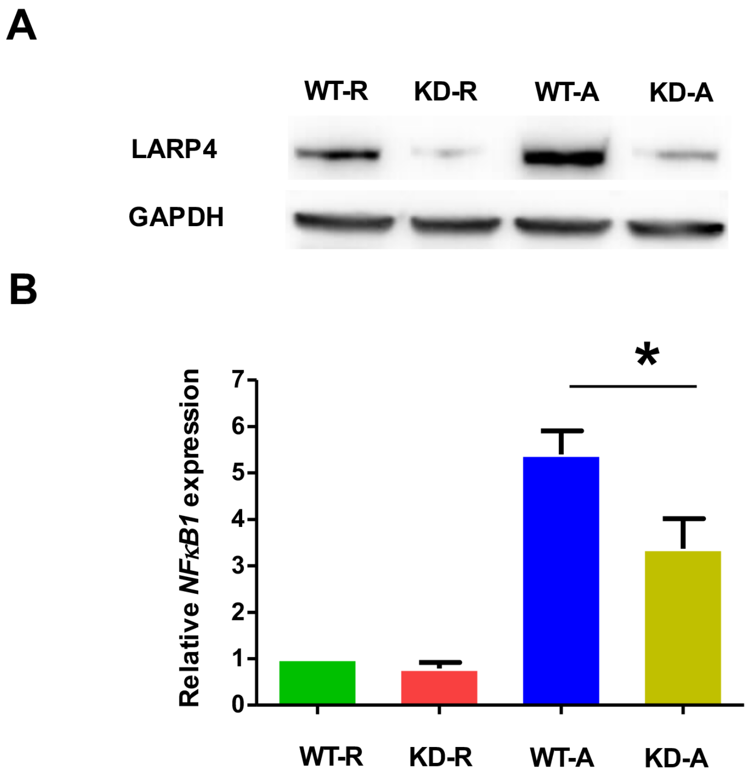


**Figure S11. The effect of *LARP4* knockdown (KD) on *NFκB1* expression in human CD4$^+$ T cells.**

Human CD4$^+$ T cells were transfected with *LARP4* siRNA or control RNA. 48 h after transfection, cells were activated by CD3/CD28 for an additional 16 hours. (A) Western Blotting analysis of the LARP4 protein in human resting and activated CD4$^+$ T cells with and without *LARP4* KD. GAPDH was used as an internal control. (B) RT-qPCR analysis of the relative expression level of the *NFκB1* mRNA in human resting and activated CD4$^+$ T cells with and without *LARP4* KD. *GAPDH* was used as the normalizing control. The error bars represent ±SEM based on three biological replicates (* $p < 0.05$).

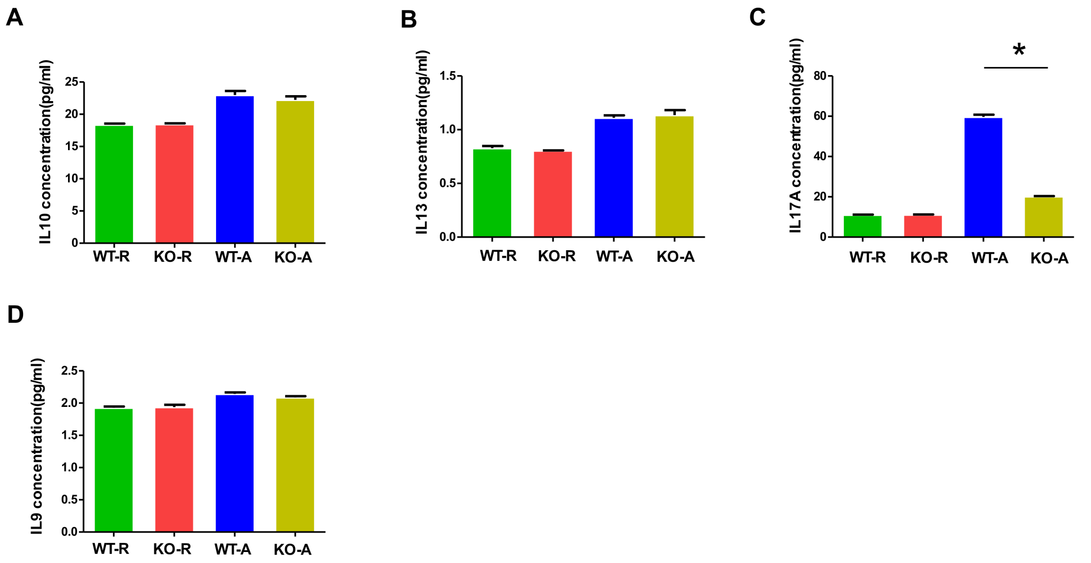


**Figure S12. Cytokine production by WT and LARP4 KO CD4$^+$ T cells.**

Cytokines secreted by resting and activated CD4$^+$ T cells were analyzed in WT and LARP4 KO mice. The levels of IL-10 (**A**), IL-13 (**B**), IL-17A **(C)** and IL-9 (**D**) in the culture supernatant were determined by the LEGENDplex™ Cytometric Bead Assay (BioLegend). R and A represent resting and activated CD4$^+$ T cells, respectively. Error bars represent ±SEM from three biological replicates (*$p < 0.05$).

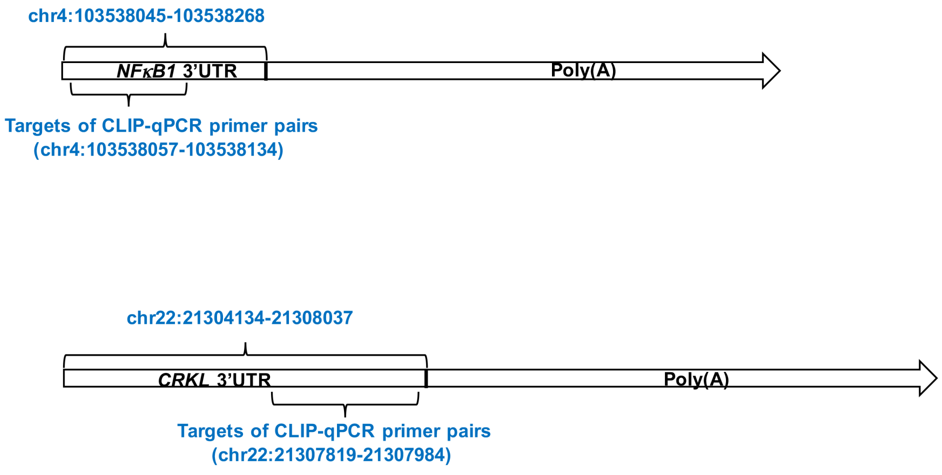


**Figure S13. The coordinates of the CLIP-qPCR primer pairs in the 3'UTR of NFkB1 and CRKL.**

**Table S1. Primers used for RT-PCR validation of the spliced and intron-retained transcript level in human.**

| Gene name | Primer name | Primer sequence (5´ to 3´) |
|---|---|---|
| ***NFκB1*** | NFκB1_E6_F | AACTGCTGGACCCAAGGACA |
| | NFκB1_I6_R | GTGGTCACCTTACTTCCTGGA |
| | NFκB1_E7_R | ACGCCTCTGTCATTCGTGCT |
| ***CRKL*** | CRKL_E1_F | CCACTACCTGGACACCACCA |
| | CRKL_I1_R | CCCCTATACAAGCACTCTTCG |
| | CRKL_E2_R | AAAGGGCAGGTCTTCGGCAT |
| ***PSMD7*** | PSMD7_E3_F | TTTGATGAAGATGACAAAGACGA |
| | PSMD7_E4_R | AGGACAGTATCTTTTCATGAGTTCG |
| ***PSMD12*** | PSMD12_E5_F | ATGGTGATGTGAAAGAGGCA |
| | PSMD12_E6_R | TAGGCAGAGCCTCATTTGCT |
| ***SRSF3*** | SRSF3_E2_F | CTGATGCAGTCCGAGAGCTA |
| | SRSF3_E3_R | GAGGTGGGCCACGATTTCTA |
| ***HNRNPU*** | HNRNPU_E12_F | ACAGAGGGAACTACAACCAGAACT |
| | HNRNPU_E13_R | ACCCTGCTGCCACTGGTTG |
| ***RELB*** | RELB_E4_F | CCATCGAGCTCCGGGATTGT |
| | RELB_E5_R | CAGTCCTTCCACACCAGGCA |
| ***REL*** | REL_E1_F | GGCCTCCTGACTGACTGACTG |
| | REL_E2_R | CCCTCTGCCTGGGTTGTTCA |
| ***CD40LG*** | CD40LG_E1_F | ATTGGGTCAGCACTTTTTGC |
| | CD40LG_E2_R | TCACAAAGCCTTCAAACTGG |
| ***GAPDH*** | GAPDH_F | CAAGAAGGTGGTGAAGCAGG |
| | GAPDH_R | AAAGGTGGAGGAGTGGGTGT |
| ***B2M*** | B2M-F | CCTGAATTGCTATGTGTCTG |
| | B2M-R | CTAACTATCTTGGGCTGTGA |
| ***ACTB*** | ACTB-F | GAGACCTTCAACACCCCAG |
| | ACTB-R | TCACGCACGATTTCCCGCT |
| ***HPRT1*** | HPRT1-F | TTCAGGCGGCTGCGACGAG |
| | HPRT1-R | GTGGCGGAGCAGAGGAGGA |
| ***SDHA*** | SDHA-F | TGATCTTCGCTGGCGTGGAC |
| | SDHA-R | CTGCCCCTTGTAGTTGGTGG |
| ***HSP90AB1*** | HSP90AB1-F | ATGAGGAGGATGACAGCGGT |
| | HSP90AB1-R | GGGTTTCTGGTCCAAATAGGC |
| ***YWHAB*** | YWHAB-F | CAGAAAGCCAAACTCGCTGA |
| | YWHAB-R | AATGCTGGAGATGACACGCC |
| ***YWHAQ*** | YWHAQ-F | CGCTCCCTCTCTGCTTTTTG |
| | YWHAQ-R | GCTCGGTCACTGCCTTCATG |
| ***PTMA*** | PTMA-F | AATGAGGAAAATGGGGAGCAG |
| | PTMA-R | TCACCTTCTTCTTCCTCCT |
| ***YWHAZ*** | YWHAZ-F | CAACAAGCATACCAAGAAGC |
| | YWHAZ-R | GAACACAGAGAAGTTAAGGGC |
| ***UBC*** | UBC-F | ATTTGGGTCGCGGTTCTTG |
| | UBC-R | TGCCTTGACATTCTCGATGGT |
| ***TBP*** | TBP-F | TGTGCTGGAGATGCTCTAGG |
| | TBP-R | GTAAGGTGGCAGGCTGTTGT |

**Note: E6 denotes the primer locates at exon6 while I6 means the primer locates at intron 6. F represents forward primer of mRNA while R denotes reverse primer.**

**Table S2. Primers used for RT-PCR validation of the spliced transcript level in mouse.**

| Gene | Primer name | Primer sequence (5´ to 3´) |
|---|---|---|

| name | | |
|---|---|---|
| *Nfκb1* | mNFκB1_E7_F | TATGCACCGTAACAGCAGGA |
| | mNFκB1_E8_R | CCGTGCTTCCAGTGTTTCAA |
| *Crkl* | mCRKL_E2_F | CGATCAACCCTTTGCCATCC |
| | mCRKL_E3_R | ATTCACCTCGCCTTCCCATT |
| *Il2* | mIL2-E3-F | AGATGAACTTGGACCTCTGC |
| | mIL2-E4-R | ATGTGTTGTCAGAGCCCTTT |
| *Ifnγ* | mIFNG-E3-F | TTCTTCAGCAACAGCAAGGC |
| | mIFNG-E4-R | GAATCAGCAGCGACTCCTTT |
| *Larp4* | mLARP4-E1-F | GCTTTGGAGCAGGTGACATC |
| | mLARP4-E2-R | TCATGCCAAGAGGGTTCAGT |

**Table S3. Primers used for Pol II ChIP-qPCR validation in human.**

| Gene name | Primer name | Primer sequence (5´ to 3´) |
|---|---|---|
| *NFκB1* | NFκB1_ChIP_F | CCTAGAAGTGCGGGCTTCCC |
| | NFκB1_ChIP_R | CCACTGACGTCGAGAGAGCA |
| *STAT1* | STAT1_ChIP_F | CAGACTCTGCGCAGGAAAGCGA |
| | STAT1_ChIP_R | CGTCTAATTGGCTGAGCGCGGA |
| *CRKL* | CRKL_ChIP_F | CACGTTACGCTTTGTTCCCA |
| | CRKL_ChIP-R | CGGTAGGCACTCTCTTCCTG |

**Table S4. Primers used for Pol II ChIP-qPCR validation in mouse.**

| Gene name | Primer name | Primer sequence (5´ to 3´) |
|---|---|---|
| *Nfκb1* | mNFκB1_F | CCTCGGTACTTCTCTCTCGT |
| | mNFκB1_R | CTCGACGTCAGTGGGAATTT |
| *Crkl* | mCRKL_ChIP_F | GTCCAGTCATCCTCACCAAC |
| | mCRKL_ChIP_R | CCAGAAGGCCCTGGTAAATC |
| *Il2* | mIL2_F | GAGCTTGAAGTGGAGCTTGA |
| | mIL2-R | AAATTGCCTCCCATGCTGAA |

**Table S5. Primers used for LARP4 CLIP-qPCR validation in human.**

| Gene name | Primer name | Primer sequence (5´ to 3´) |
|---|---|---|
| *NFκB1* | NFκB1_CLIP_F | GCTGTTGTCCCTCTGCTACG |
| | NFκB1_CLIP_R | TGCTGTGGTCAGAAGGAATGC |
| *CRKL* | CRKL_CLIP_F | TTTGGGGATCATAGCTTGTTTT |
| | CRKL_CLIP_R | CCATGGAAACAAGGGTTTTG |

**Table S6. The complete list for the upregulated genes with increased Pol II occupancy, increased splicing efficiency (PST) and/or increased stability (βs) upon activation in human T cells.**

| Gene name \ Regulatory factor | Pol II | PST | βs |
|---|---|---|---|
| *RNF14* | TRUE | TRUE | TRUE |
| *C16orf13* | TRUE | FALSE | FALSE |
| *COPRS* | TRUE | TRUE | TRUE |
| *ZC3H10* | TRUE | TRUE | FALSE |
| *ZC3H14* | TRUE | TRUE | TRUE |
| *GRINA* | TRUE | TRUE | FALSE |
| *NUP93* | TRUE | TRUE | TRUE |
| *GOLIM4* | TRUE | TRUE | FALSE |
| *SPPL2A* | TRUE | FALSE | TRUE |
| *CYP27B1* | TRUE | TRUE | FALSE |
| *BCL2A1* | TRUE | FALSE | TRUE |
| *CYFIP1* | TRUE | TRUE | FALSE |
| *SLC36A1* | TRUE | TRUE | TRUE |
| *CRTAM* | TRUE | TRUE | FALSE |
| *IPO11* | TRUE | TRUE | TRUE |
| *IPO13* | TRUE | TRUE | TRUE |
| *SRBD1* | TRUE | TRUE | FALSE |
| *MCM10* | TRUE | TRUE | TRUE |
| *AAGAB* | TRUE | TRUE | TRUE |
| *XPNPEP1* | TRUE | TRUE | TRUE |
| *ZNF57* | TRUE | FALSE | FALSE |
| *GGCT* | TRUE | TRUE | TRUE |
| *IGFBP4* | TRUE | FALSE | TRUE |
| *EIF2B4* | TRUE | TRUE | TRUE |
| *EIF2B3* | TRUE | TRUE | TRUE |
| *EIF2B1* | TRUE | FALSE | TRUE |
| *KIAA0020* | TRUE | TRUE | TRUE |
| *TMEM141* | TRUE | FALSE | FALSE |
| *RTKN2* | TRUE | FALSE | TRUE |
| *VMP1* | TRUE | TRUE | TRUE |
| *C19orf24* | TRUE | TRUE | FALSE |
| *MYBL2* | TRUE | FALSE | FALSE |
| *TIPIN* | TRUE | TRUE | TRUE |
| *AP2A1* | TRUE | TRUE | FALSE |
| *NSUN2* | TRUE | FALSE | TRUE |
| *TUBG1* | TRUE | FALSE | TRUE |

| | | | |
|---|---|---|---|
| *TIMM44* | TRUE | TRUE | TRUE |
| *RRP7A* | TRUE | FALSE | TRUE |
| *RRP7B* | TRUE | FALSE | TRUE |
| *SPG21* | TRUE | FALSE | FALSE |
| *PTPN11* | TRUE | TRUE | TRUE |
| *C16orf70* | TRUE | TRUE | TRUE |
| *MRPL23* | TRUE | FALSE | FALSE |
| *MRPL28* | TRUE | FALSE | TRUE |
| *NPTX1* | TRUE | TRUE | FALSE |
| *SRSF4* | TRUE | TRUE | TRUE |
| *GID8* | TRUE | FALSE | TRUE |
| *ZNF583* | TRUE | FALSE | TRUE |
| *CAMKK2* | TRUE | FALSE | TRUE |
| *CTPS1* | TRUE | TRUE | TRUE |
| *SRF* | TRUE | FALSE | TRUE |
| *SRI* | TRUE | TRUE | TRUE |
| *SRM* | TRUE | TRUE | FALSE |
| *EIF2AK4* | TRUE | TRUE | TRUE |
| *MYO19* | TRUE | FALSE | FALSE |
| *FAM136A* | TRUE | TRUE | TRUE |
| *MPDU1* | TRUE | FALSE | TRUE |
| *GCNT1* | TRUE | FALSE | FALSE |
| *LPCAT1* | TRUE | FALSE | TRUE |
| *PIGU* | TRUE | TRUE | TRUE |
| *RMND1* | TRUE | TRUE | TRUE |
| *ANKLE1* | TRUE | TRUE | TRUE |
| *ANKLE2* | TRUE | TRUE | TRUE |
| *C9orf114* | TRUE | FALSE | TRUE |
| *SLC39A14* | TRUE | TRUE | TRUE |
| *EIF1AD* | TRUE | FALSE | TRUE |
| *EVI5* | TRUE | TRUE | TRUE |
| *NARFL* | TRUE | TRUE | TRUE |
| *PTPMT1* | TRUE | FALSE | TRUE |
| *CENPBD1P1* | TRUE | FALSE | TRUE |
| *DAG1* | TRUE | TRUE | TRUE |
| *HELLS* | TRUE | FALSE | TRUE |
| *GGA1* | TRUE | FALSE | TRUE |
| *PKD2* | TRUE | TRUE | TRUE |
| *SPRYD3* | TRUE | FALSE | TRUE |
| *PSMB5* | TRUE | TRUE | TRUE |
| *PSMB2* | TRUE | FALSE | TRUE |
| *KCNK5* | TRUE | FALSE | FALSE |
| *KCNK6* | TRUE | FALSE | TRUE |
| *MLH1* | TRUE | TRUE | TRUE |

| | | | |
|---|---|---|---|
| *NUP160* | TRUE | TRUE | TRUE |
| *GNA12* | TRUE | FALSE | TRUE |
| *GNA11* | TRUE | FALSE | TRUE |
| *CHTF18* | TRUE | FALSE | FALSE |
| *BCAT2* | TRUE | TRUE | FALSE |
| *GTF2F2* | TRUE | TRUE | TRUE |
| *EIF2A* | TRUE | FALSE | TRUE |
| *ODF2* | TRUE | TRUE | TRUE |
| *RRP1B* | TRUE | FALSE | TRUE |
| *CCL20* | TRUE | TRUE | TRUE |
| *MAP3K8* | TRUE | FALSE | FALSE |
| *ACAT1* | TRUE | TRUE | TRUE |
| *IVD* | TRUE | TRUE | TRUE |
| *IL2RA* | TRUE | FALSE | TRUE |
| *IL2RB* | TRUE | FALSE | TRUE |
| *SLC37A3* | TRUE | TRUE | TRUE |
| *CDK12* | TRUE | FALSE | TRUE |
| *GPR125* | TRUE | TRUE | FALSE |
| *NCL* | TRUE | TRUE | TRUE |
| *SETD6* | TRUE | TRUE | TRUE |
| *CCDC152* | TRUE | TRUE | TRUE |
| *WRAP73* | TRUE | TRUE | TRUE |
| *MTCH2* | TRUE | FALSE | TRUE |
| *RINL* | TRUE | TRUE | TRUE |
| *ICMT* | TRUE | FALSE | TRUE |
| *EME1* | TRUE | FALSE | TRUE |
| *ATP6V0A2* | TRUE | TRUE | TRUE |
| *AAMP* | TRUE | FALSE | TRUE |
| *TTC26* | TRUE | TRUE | TRUE |
| *DOT1L* | TRUE | FALSE | TRUE |
| *ENO1* | TRUE | FALSE | FALSE |
| *ACOX3* | TRUE | TRUE | TRUE |
| *NSMCE2* | TRUE | FALSE | FALSE |
| *ARHGDIA* | TRUE | FALSE | TRUE |
| *NANP* | TRUE | TRUE | FALSE |
| *DDX21* | TRUE | FALSE | TRUE |
| *ZNF90* | TRUE | TRUE | FALSE |
| *TBX21* | TRUE | TRUE | TRUE |
| *TACO1* | TRUE | TRUE | TRUE |
| *TRUB1* | TRUE | TRUE | TRUE |
| *TRUB2* | TRUE | FALSE | TRUE |
| *CCDC77* | TRUE | TRUE | TRUE |
| *AAAS* | TRUE | FALSE | TRUE |
| *MANEAL* | TRUE | TRUE | TRUE |

| | | | |
|---|---|---|---|
| *CDC16* | TRUE | TRUE | TRUE |
| *MAP1S* | TRUE | FALSE | TRUE |
| *AGFG1* | TRUE | FALSE | TRUE |
| *ASNA1* | TRUE | FALSE | FALSE |
| *SDR42E1* | TRUE | FALSE | TRUE |
| *PPCDC* | TRUE | FALSE | TRUE |
| *SLC43A3* | TRUE | TRUE | TRUE |
| *SLC43A1* | TRUE | TRUE | TRUE |
| *NAA10* | TRUE | FALSE | FALSE |
| *RRP36* | TRUE | FALSE | TRUE |
| *RPA2* | TRUE | FALSE | TRUE |
| *UFD1L* | TRUE | TRUE | FALSE |
| *TPX2* | TRUE | TRUE | FALSE |
| *INPP4B* | TRUE | FALSE | TRUE |
| *MICAL2* | TRUE | FALSE | FALSE |
| *ZNF367* | TRUE | TRUE | FALSE |
| *H2AFY* | TRUE | FALSE | FALSE |
| *NCAPG2* | TRUE | TRUE | TRUE |
| *NMD3* | TRUE | FALSE | TRUE |
| *MAP7D3* | TRUE | TRUE | TRUE |
| *FOXP3* | TRUE | TRUE | TRUE |
| *POLDIP2* | TRUE | TRUE | TRUE |
| *GTDC1* | TRUE | TRUE | FALSE |
| *ALDH18A1* | TRUE | TRUE | TRUE |
| *CPNE7* | TRUE | FALSE | FALSE |
| *SNRPA* | TRUE | TRUE | TRUE |
| *ZDHHC16* | TRUE | TRUE | TRUE |
| *PRKAG1* | TRUE | TRUE | TRUE |
| *ESPL1* | TRUE | FALSE | TRUE |
| *LARP4* | TRUE | TRUE | TRUE |
| *DDX49* | TRUE | FALSE | TRUE |
| *DDX41* | TRUE | TRUE | FALSE |
| *ZNF175* | TRUE | TRUE | TRUE |
| *ELAVL1* | TRUE | FALSE | TRUE |
| *SETBP1* | TRUE | FALSE | FALSE |
| *FARSB* | TRUE | TRUE | TRUE |
| *NFIL3* | TRUE | FALSE | TRUE |
| *CCDC58* | TRUE | FALSE | TRUE |
| *SLC35B4* | TRUE | TRUE | TRUE |
| *PELP1* | TRUE | TRUE | TRUE |
| *CDC37* | TRUE | FALSE | FALSE |
| *ALAS1* | TRUE | TRUE | TRUE |
| *LONRF3* | TRUE | TRUE | TRUE |
| *ANKRD16* | TRUE | TRUE | TRUE |

| | | | |
|---|---|---|---|
| *CCNA2* | TRUE | TRUE | TRUE |
| *ATP13A3* | TRUE | TRUE | TRUE |
| *SKA3* | TRUE | TRUE | FALSE |
| *DDRGK1* | TRUE | FALSE | FALSE |
| *E2F5* | TRUE | TRUE | TRUE |
| *RPGR* | TRUE | FALSE | TRUE |
| *URB2* | TRUE | TRUE | TRUE |
| *URB1* | TRUE | TRUE | TRUE |
| *EIF2B2* | TRUE | TRUE | TRUE |
| *DFFA* | TRUE | FALSE | TRUE |
| *SEC24D* | TRUE | FALSE | TRUE |
| *SEC24A* | TRUE | FALSE | TRUE |
| *MRPL45* | TRUE | TRUE | TRUE |
| *CCDC6* | TRUE | TRUE | TRUE |
| *MTHFD2* | TRUE | FALSE | TRUE |
| *GMNN* | TRUE | FALSE | TRUE |
| *ZNF341* | TRUE | TRUE | TRUE |
| *ABRACL* | TRUE | FALSE | TRUE |
| *ZNF432* | TRUE | TRUE | TRUE |
| *NUDT19* | TRUE | TRUE | TRUE |
| *DRG1* | TRUE | FALSE | TRUE |
| *NUP107* | TRUE | TRUE | TRUE |
| *AP1B1* | TRUE | TRUE | TRUE |
| *TXLNA* | TRUE | FALSE | TRUE |
| *IL12RB2* | TRUE | TRUE | TRUE |
| *ACOT4* | TRUE | FALSE | TRUE |
| *ADCY3* | TRUE | FALSE | FALSE |
| *KIF23* | TRUE | FALSE | TRUE |
| *RBBP4* | TRUE | FALSE | TRUE |
| *FAM208B* | TRUE | TRUE | TRUE |
| *PTPLAD1* | TRUE | FALSE | TRUE |
| *C12orf4* | TRUE | TRUE | TRUE |
| *XRCC3* | TRUE | TRUE | TRUE |
| *XRCC2* | TRUE | TRUE | TRUE |
| *TFRC* | TRUE | FALSE | TRUE |
| *CTLA4* | TRUE | TRUE | FALSE |
| *TFR2* | TRUE | FALSE | FALSE |
| *MIER2* | TRUE | TRUE | TRUE |
| *LMNB2* | TRUE | TRUE | TRUE |
| *LMNB1* | TRUE | FALSE | TRUE |
| *CD274* | TRUE | FALSE | TRUE |
| *NAMPT* | TRUE | FALSE | TRUE |
| *ACACA* | TRUE | TRUE | TRUE |
| *PITRM1* | TRUE | TRUE | TRUE |

| | | | |
|---|---|---|---|
| *SARS2* | TRUE | FALSE | TRUE |
| *GUSBP1* | TRUE | TRUE | TRUE |
| *THUMPD2* | TRUE | TRUE | TRUE |
| *NUP188* | TRUE | TRUE | TRUE |
| *GTPBP4* | TRUE | FALSE | TRUE |
| *GTPBP3* | TRUE | FALSE | FALSE |
| *BCL6* | TRUE | TRUE | FALSE |
| *PLK4* | TRUE | TRUE | TRUE |
| *CSNK1G1* | TRUE | TRUE | TRUE |
| *PCGF6* | TRUE | TRUE | TRUE |
| *ELP6* | TRUE | FALSE | TRUE |
| *ELP5* | TRUE | FALSE | TRUE |
| *CCDC86* | TRUE | FALSE | TRUE |
| *PTGFRN* | TRUE | TRUE | TRUE |
| *UBAP2* | TRUE | TRUE | TRUE |
| *EBNA1BP2* | TRUE | TRUE | TRUE |
| *ZBTB32* | TRUE | FALSE | TRUE |
| *TMEM97* | TRUE | FALSE | TRUE |
| *SGTA* | TRUE | FALSE | TRUE |
| *PEX5* | TRUE | TRUE | TRUE |
| *ME3* | TRUE | TRUE | FALSE |
| *ME2* | TRUE | TRUE | TRUE |
| *MPZL2* | TRUE | TRUE | FALSE |
| *AURKAIP1* | TRUE | TRUE | FALSE |
| *NFKBIB* | TRUE | TRUE | TRUE |
| *PREP* | TRUE | FALSE | TRUE |
| *RNH1* | TRUE | FALSE | FALSE |
| *CLPP* | TRUE | FALSE | TRUE |
| *CLPB* | TRUE | TRUE | TRUE |
| *RRAGD* | TRUE | FALSE | TRUE |
| *GGT1* | TRUE | TRUE | FALSE |
| *MCOLN1* | TRUE | FALSE | FALSE |
| *MCOLN2* | TRUE | TRUE | TRUE |
| *STYXL1* | TRUE | TRUE | TRUE |
| *CPM* | TRUE | TRUE | TRUE |
| *IFT22* | TRUE | TRUE | TRUE |
| *RNASEH1* | TRUE | FALSE | TRUE |
| *DUSP6* | TRUE | FALSE | FALSE |
| *SDAD1* | TRUE | TRUE | TRUE |
| *ATAD3B* | TRUE | TRUE | TRUE |
| *GDI2* | TRUE | FALSE | TRUE |
| *ARL5A* | TRUE | FALSE | TRUE |
| *ERLIN1* | TRUE | TRUE | TRUE |
| *WDR36* | TRUE | FALSE | TRUE |

| | | | |
|---|---|---|---|
| *IL15RA* | TRUE | FALSE | TRUE |
| *ABHD17C* | TRUE | FALSE | TRUE |
| *GIGYF2* | TRUE | TRUE | TRUE |
| *ZFP69B* | TRUE | TRUE | TRUE |
| *CDC6* | TRUE | FALSE | TRUE |
| *CDC7* | TRUE | FALSE | TRUE |
| *CRYZL1* | TRUE | FALSE | TRUE |
| *TFPT* | TRUE | TRUE | FALSE |
| *TM9SF4* | TRUE | TRUE | TRUE |
| *COX5A* | TRUE | FALSE | TRUE |
| *TTLL4* | TRUE | FALSE | TRUE |
| *SLC35F2* | TRUE | TRUE | TRUE |
| *REXO4* | TRUE | FALSE | TRUE |
| *SLC25A12* | TRUE | TRUE | TRUE |
| *SLC25A10* | TRUE | TRUE | FALSE |
| *NDUFA13* | TRUE | FALSE | FALSE |
| *SYNCRIP* | TRUE | TRUE | TRUE |
| *MMAA* | TRUE | FALSE | TRUE |
| *ALG8* | TRUE | TRUE | TRUE |
| *ALG3* | TRUE | FALSE | TRUE |
| *CCNE2* | TRUE | FALSE | TRUE |
| *CCNE1* | TRUE | TRUE | TRUE |
| *IRF4* | TRUE | FALSE | TRUE |
| *IRF8* | TRUE | FALSE | TRUE |
| *RUNDC1* | TRUE | TRUE | FALSE |
| *PANX1* | TRUE | TRUE | TRUE |
| *TRIM69* | TRUE | TRUE | TRUE |
| *MTOR* | TRUE | TRUE | TRUE |
| *UNG* | TRUE | TRUE | TRUE |
| *TRIT1* | TRUE | TRUE | TRUE |
| *C2orf69* | TRUE | FALSE | TRUE |
| *SNHG15* | TRUE | FALSE | TRUE |
| *SNHG17* | TRUE | FALSE | TRUE |
| *SNHG16* | TRUE | FALSE | TRUE |
| *DHCR24* | TRUE | FALSE | TRUE |
| *HSF5* | TRUE | TRUE | FALSE |
| *RAPH1* | TRUE | TRUE | TRUE |
| *PARK7* | TRUE | FALSE | FALSE |
| *MDM2* | TRUE | TRUE | TRUE |
| *SNRPD3* | TRUE | FALSE | TRUE |
| *SNRPD1* | TRUE | TRUE | TRUE |
| *SYNGR2* | TRUE | TRUE | FALSE |
| *SLC25A15* | TRUE | TRUE | TRUE |
| *PC* | TRUE | TRUE | FALSE |

| | | | |
|---|---|---|---|
| *FAM174A* | TRUE | FALSE | TRUE |
| *FBL* | TRUE | FALSE | FALSE |
| *DOHH* | TRUE | TRUE | TRUE |
| *GCFC2* | TRUE | TRUE | TRUE |
| *HPRT1* | TRUE | TRUE | FALSE |
| *PTPN6* | TRUE | TRUE | TRUE |
| *FOSL1* | TRUE | FALSE | TRUE |
| *CD63* | TRUE | FALSE | FALSE |
| *SHCBP1* | TRUE | TRUE | TRUE |
| *WDR12* | TRUE | TRUE | TRUE |
| *WDR18* | TRUE | TRUE | FALSE |
| *TIMM9* | TRUE | TRUE | TRUE |
| *C1orf216* | TRUE | FALSE | TRUE |
| *ZMYND19* | TRUE | FALSE | TRUE |
| *C10orf35* | TRUE | FALSE | TRUE |
| *PKN3* | TRUE | FALSE | FALSE |
| *TEX261* | TRUE | TRUE | TRUE |
| *CRCP* | TRUE | TRUE | TRUE |
| *KCTD17* | TRUE | FALSE | TRUE |
| *SLC25A32* | TRUE | TRUE | TRUE |
| *SLC25A39* | TRUE | FALSE | FALSE |
| *TUBA1C* | TRUE | TRUE | TRUE |
| *TAF6* | TRUE | FALSE | FALSE |
| *G6PD* | TRUE | FALSE | FALSE |
| *HMOX1* | TRUE | TRUE | TRUE |
| *NPLOC4* | TRUE | TRUE | TRUE |
| *AP2M1* | TRUE | TRUE | FALSE |
| *MINA* | TRUE | TRUE | TRUE |
| *SSRP1* | TRUE | TRUE | FALSE |
| *C1orf174* | TRUE | TRUE | TRUE |
| *RPIA* | TRUE | TRUE | TRUE |
| *LRP12* | TRUE | FALSE | TRUE |
| *BBS4* | TRUE | TRUE | TRUE |
| *AGL* | TRUE | TRUE | TRUE |
| *NIPA1* | TRUE | TRUE | TRUE |
| *CS* | TRUE | FALSE | TRUE |
| *SCFD2* | TRUE | FALSE | FALSE |
| *PDCD10* | TRUE | FALSE | TRUE |
| *PDCD11* | TRUE | TRUE | TRUE |
| *DIMT1* | TRUE | TRUE | TRUE |
| *MEN1* | TRUE | TRUE | TRUE |
| *ERI3* | TRUE | TRUE | FALSE |
| *ACLY* | TRUE | TRUE | TRUE |
| *WSB2* | TRUE | TRUE | TRUE |

| | | | |
|---|---|---|---|
| *ANAPC1* | TRUE | TRUE | FALSE |
| *ANAPC7* | TRUE | TRUE | TRUE |
| *PISD* | TRUE | TRUE | TRUE |
| *PSMD14* | TRUE | FALSE | TRUE |
| *PSMD11* | TRUE | FALSE | TRUE |
| *PSMD12* | TRUE | TRUE | TRUE |
| *UTP20* | TRUE | TRUE | TRUE |
| *METTL2B* | TRUE | TRUE | TRUE |
| *TELO2* | TRUE | FALSE | FALSE |
| *ARF6* | TRUE | FALSE | TRUE |
| *RAB11FIP1* | TRUE | TRUE | TRUE |
| *FPGS* | TRUE | FALSE | TRUE |
| *FAR1* | TRUE | TRUE | FALSE |
| *WDR77* | TRUE | TRUE | TRUE |
| *NQO1* | TRUE | TRUE | FALSE |
| *DOLPP1* | TRUE | FALSE | TRUE |
| *ELK1* | TRUE | TRUE | TRUE |
| *C4orf27* | TRUE | TRUE | TRUE |
| *TMEM194A* | TRUE | TRUE | TRUE |
| *TMEM214* | TRUE | TRUE | TRUE |
| *ANKRD13B* | TRUE | FALSE | FALSE |
| *PPA1* | TRUE | FALSE | FALSE |
| *FBXW9* | TRUE | FALSE | TRUE |
| *ETFA* | TRUE | FALSE | TRUE |
| *YKT6* | TRUE | TRUE | TRUE |
| *BABAM1* | TRUE | FALSE | FALSE |
| *SMARCA4* | TRUE | TRUE | FALSE |
| *PACRGL* | TRUE | FALSE | TRUE |
| *NLE1* | TRUE | FALSE | TRUE |
| *DPH2* | TRUE | TRUE | TRUE |
| *GPAM* | TRUE | TRUE | TRUE |
| *TAF4B* | TRUE | TRUE | TRUE |
| *EIF4ENIF1* | TRUE | TRUE | TRUE |
| *CUL7* | TRUE | TRUE | TRUE |
| *TRIM25* | TRUE | FALSE | FALSE |
| *TRIM21* | TRUE | FALSE | TRUE |
| *GNPAT* | TRUE | FALSE | TRUE |
| *EDARADD* | TRUE | TRUE | TRUE |
| *SP140* | TRUE | FALSE | TRUE |
| *GAPDH* | TRUE | FALSE | FALSE |
| *DNAJC21* | TRUE | FALSE | TRUE |
| *DTD1* | TRUE | TRUE | TRUE |
| *DTD2* | TRUE | FALSE | TRUE |
| *AEN* | TRUE | FALSE | FALSE |

| | | | |
|---|---|---|---|
| *TMEM70* | TRUE | FALSE | TRUE |
| *MRPL4* | TRUE | TRUE | TRUE |
| *MRPL3* | TRUE | FALSE | TRUE |
| *ZNF613* | TRUE | TRUE | TRUE |
| *SLC3A2* | TRUE | FALSE | TRUE |
| *EIF4EBP1* | TRUE | FALSE | TRUE |
| *BAG1* | TRUE | FALSE | TRUE |
| *BAG4* | TRUE | FALSE | TRUE |
| *GMFB* | TRUE | TRUE | TRUE |
| *UTP11L* | TRUE | FALSE | TRUE |
| *CPSF3L* | TRUE | TRUE | FALSE |
| *ATXN10* | TRUE | TRUE | FALSE |
| *MRPS17* | TRUE | FALSE | TRUE |
| *ILVBL* | TRUE | FALSE | TRUE |
| *TSTA3* | TRUE | FALSE | FALSE |
| *MAPKAP1* | TRUE | TRUE | FALSE |
| *NRDE2* | TRUE | TRUE | TRUE |
| *CDK2AP1* | TRUE | TRUE | FALSE |
| *ZSCAN21* | TRUE | TRUE | TRUE |
| *ZSCAN22* | TRUE | FALSE | TRUE |
| *MRPS5* | TRUE | FALSE | TRUE |
| *IFI35* | TRUE | FALSE | FALSE |
| *MRPS2* | TRUE | FALSE | TRUE |
| *ABHD2* | TRUE | TRUE | TRUE |
| *ABHD6* | TRUE | TRUE | TRUE |
| *PA2G4* | TRUE | TRUE | TRUE |
| *EIF4G3* | TRUE | TRUE | FALSE |
| *EIF4G1* | TRUE | TRUE | TRUE |
| *POLR2D* | TRUE | TRUE | TRUE |
| *POLR2F* | TRUE | FALSE | FALSE |
| *POLR2H* | TRUE | FALSE | TRUE |
| *ASCC3* | TRUE | FALSE | TRUE |
| *AGO1* | TRUE | TRUE | TRUE |
| *FAM120A* | TRUE | TRUE | TRUE |
| *B3GALNT2* | TRUE | TRUE | TRUE |
| *MRTO4* | TRUE | FALSE | TRUE |
| *BCL2L14* | TRUE | TRUE | FALSE |
| *NUP43* | TRUE | TRUE | TRUE |
| *CTU2* | TRUE | TRUE | TRUE |
| *NCKAP1* | TRUE | TRUE | FALSE |
| *TARBP2* | TRUE | TRUE | TRUE |
| *ATF3* | TRUE | TRUE | TRUE |
| *PPAP2A* | TRUE | FALSE | TRUE |
| *SUMO3* | TRUE | FALSE | FALSE |

| | | | |
|---|---|---|---|
| *EEF2K* | TRUE | FALSE | TRUE |
| *NADK2* | TRUE | TRUE | TRUE |
| *SLC38A5* | TRUE | TRUE | FALSE |
| *SLC38A7* | TRUE | FALSE | TRUE |
| *YBX3* | TRUE | FALSE | FALSE |
| *TNFRSF9* | TRUE | FALSE | TRUE |
| *TNFRSF4* | TRUE | FALSE | FALSE |
| *FUNDC1* | TRUE | TRUE | TRUE |
| *CEP170B* | TRUE | FALSE | FALSE |
| *DYNC1LI1* | TRUE | FALSE | TRUE |
| *ACP2* | TRUE | FALSE | TRUE |
| *ANKRD39* | TRUE | FALSE | TRUE |
| *NT5C3B* | TRUE | TRUE | FALSE |
| *CISH* | TRUE | FALSE | TRUE |
| *SLC27A4* | TRUE | TRUE | FALSE |
| *SLC27A2* | TRUE | TRUE | TRUE |
| *AK2* | TRUE | FALSE | TRUE |
| *RRP9* | TRUE | FALSE | TRUE |
| *BRMS1L* | TRUE | FALSE | TRUE |
| *MATK* | TRUE | FALSE | FALSE |
| *KDM1B* | TRUE | TRUE | TRUE |
| *IMPDH2* | TRUE | TRUE | FALSE |
| *MTMR4* | TRUE | TRUE | FALSE |
| *MTMR2* | TRUE | TRUE | TRUE |
| *TRAIP* | TRUE | TRUE | TRUE |
| *EXOSC8* | TRUE | FALSE | TRUE |
| *BCKDK* | TRUE | FALSE | TRUE |
| *EXOSC2* | TRUE | FALSE | TRUE |
| *EXOSC1* | TRUE | FALSE | TRUE |
| *EXOSC7* | TRUE | FALSE | TRUE |
| *STXBP1* | TRUE | TRUE | TRUE |
| *NOC2L* | TRUE | FALSE | TRUE |
| *FANCE* | TRUE | TRUE | TRUE |
| *FANCC* | TRUE | FALSE | FALSE |
| *FANCA* | TRUE | FALSE | FALSE |
| *HNRNPAB* | TRUE | TRUE | TRUE |
| *FANCI* | TRUE | TRUE | TRUE |
| *KNSTRN* | TRUE | TRUE | TRUE |
| *LRRFIP2* | TRUE | TRUE | TRUE |
| *CDK6* | TRUE | FALSE | TRUE |
| *CEP55* | TRUE | FALSE | FALSE |
| *SCRIB* | TRUE | FALSE | FALSE |
| *TYMS* | TRUE | TRUE | FALSE |
| *IARS* | TRUE | TRUE | TRUE |

| | | | |
|---|---|---|---|
| *CTSC* | TRUE | TRUE | TRUE |
| *TOMM34* | TRUE | FALSE | TRUE |
| *PCYT1A* | TRUE | FALSE | TRUE |
| *STIL* | TRUE | FALSE | TRUE |
| *DCTD* | TRUE | TRUE | TRUE |
| *PDP2* | TRUE | TRUE | TRUE |
| *UBA2* | TRUE | FALSE | TRUE |
| *PWP2* | TRUE | FALSE | TRUE |
| *PWP1* | TRUE | TRUE | TRUE |
| *NAA15* | TRUE | TRUE | TRUE |
| *NRROS* | TRUE | TRUE | TRUE |
| *TOP3A* | TRUE | FALSE | TRUE |
| *PNO1* | TRUE | TRUE | TRUE |
| *TALDO1* | TRUE | FALSE | FALSE |
| *BIRC5* | TRUE | FALSE | FALSE |
| *CMAHP* | TRUE | FALSE | TRUE |
| *DHDDS* | TRUE | TRUE | TRUE |
| *PARS2* | TRUE | TRUE | FALSE |
| *FAM221A* | TRUE | TRUE | TRUE |
| *SEMA7A* | TRUE | TRUE | FALSE |
| *LIN9* | TRUE | TRUE | TRUE |
| *ZNF212* | TRUE | TRUE | TRUE |
| *ZWINT* | TRUE | FALSE | FALSE |
| *NFKBID* | TRUE | TRUE | TRUE |
| *C10orf128* | TRUE | FALSE | TRUE |
| *KIAA0101* | TRUE | FALSE | FALSE |
| *IPO4* | TRUE | TRUE | TRUE |
| *IPO5* | TRUE | FALSE | TRUE |
| *DENND1A* | TRUE | FALSE | TRUE |
| *RAB8A* | TRUE | FALSE | TRUE |
| *GABPB1* | TRUE | FALSE | TRUE |
| *PAQR7* | TRUE | FALSE | TRUE |
| *FERMT3* | TRUE | TRUE | FALSE |
| *TOMM70A* | TRUE | FALSE | TRUE |
| *FADS1* | TRUE | FALSE | FALSE |
| *CSE1L* | TRUE | FALSE | TRUE |
| *MAK16* | TRUE | TRUE | TRUE |
| *KLHDC8B* | TRUE | TRUE | TRUE |
| *ZNF324B* | TRUE | TRUE | TRUE |
| *INPP1* | TRUE | FALSE | TRUE |
| *GFI1* | TRUE | TRUE | TRUE |
| *UNC45A* | TRUE | FALSE | TRUE |
| *BOD1* | TRUE | FALSE | TRUE |
| *ARHGEF10* | TRUE | TRUE | FALSE |

| | | | |
|---|---|---|---|
| *SF3A3* | TRUE | TRUE | TRUE |
| *RHBDD3* | TRUE | TRUE | TRUE |
| *CEP72* | TRUE | TRUE | TRUE |
| *CHCHD6* | TRUE | TRUE | FALSE |
| *CHCHD4* | TRUE | TRUE | TRUE |
| *SLC39A3* | TRUE | FALSE | TRUE |
| *GZMB* | TRUE | TRUE | FALSE |
| *MTHFD1L* | TRUE | FALSE | FALSE |
| *GZMH* | TRUE | TRUE | TRUE |
| *PIK3AP1* | TRUE | TRUE | TRUE |
| *NOC4L* | TRUE | FALSE | TRUE |
| *CAD* | TRUE | TRUE | FALSE |
| *PGD* | TRUE | TRUE | TRUE |
| *NUMBL* | TRUE | TRUE | TRUE |
| *COA7* | TRUE | FALSE | FALSE |
| *MRS2* | TRUE | FALSE | TRUE |
| *ARSB* | TRUE | TRUE | TRUE |
| *PAFAH1B3* | TRUE | FALSE | FALSE |
| *ADAL* | TRUE | TRUE | TRUE |
| *ZNF239* | TRUE | TRUE | TRUE |
| *UBL4A* | TRUE | FALSE | TRUE |
| *ZNF232* | TRUE | TRUE | TRUE |
| *RANBP1* | TRUE | FALSE | TRUE |
| *TXN2* | TRUE | TRUE | TRUE |
| *HDGFRP2* | TRUE | FALSE | FALSE |
| *YWHAQ* | TRUE | FALSE | TRUE |
| *ETS2* | TRUE | FALSE | TRUE |
| *CHST10* | TRUE | FALSE | TRUE |
| *AMICA1* | TRUE | TRUE | FALSE |
| *CAPRIN1* | TRUE | TRUE | TRUE |
| *VRK1* | TRUE | FALSE | TRUE |
| *VRK2* | TRUE | TRUE | TRUE |
| *SHMT2* | TRUE | FALSE | TRUE |
| *PLD6* | TRUE | FALSE | TRUE |
| *PPFIBP1* | TRUE | TRUE | TRUE |
| *ATAD3A* | TRUE | FALSE | TRUE |
| *RRP1* | TRUE | FALSE | TRUE |
| *SAAL1* | TRUE | FALSE | TRUE |
| *PLCB3* | TRUE | FALSE | TRUE |
| *SEC13* | TRUE | TRUE | TRUE |
| *MSMO1* | TRUE | TRUE | TRUE |
| *RPP30* | TRUE | FALSE | TRUE |
| *FBXO5* | TRUE | FALSE | TRUE |
| *FBXO6* | TRUE | FALSE | TRUE |

| | | | |
|---|---|---|---|
| *QSER1* | TRUE | TRUE | TRUE |
| *RITA1* | TRUE | TRUE | TRUE |
| *ATL3* | TRUE | TRUE | TRUE |
| *PPIH* | TRUE | FALSE | FALSE |
| *PPIF* | TRUE | TRUE | TRUE |
| *C14orf1* | TRUE | FALSE | TRUE |
| *AP4E1* | TRUE | TRUE | TRUE |
| *NCAPH2* | TRUE | FALSE | FALSE |
| *COG2* | TRUE | TRUE | TRUE |
| *C12orf43* | TRUE | TRUE | TRUE |
| *C12orf45* | TRUE | FALSE | TRUE |
| *ANKRD52* | TRUE | TRUE | TRUE |
| *AKR1A1* | TRUE | TRUE | FALSE |
| *EPT1* | TRUE | FALSE | TRUE |
| *NPRL3* | TRUE | TRUE | FALSE |
| *SIRT6* | TRUE | FALSE | TRUE |
| *PAK1IP1* | TRUE | TRUE | TRUE |
| *HOPX* | TRUE | FALSE | FALSE |
| *PHAX* | TRUE | FALSE | TRUE |
| *RBM28* | TRUE | FALSE | TRUE |
| *RFWD3* | TRUE | FALSE | TRUE |
| *TRMT1* | TRUE | TRUE | FALSE |
| *UGCG* | TRUE | FALSE | TRUE |
| *ARHGAP11A* | TRUE | TRUE | TRUE |
| *ARL1* | TRUE | TRUE | TRUE |
| *ZNF257* | TRUE | TRUE | TRUE |
| *RRM2* | TRUE | FALSE | TRUE |
| *NAGA* | TRUE | TRUE | TRUE |
| *PVRL1* | TRUE | FALSE | TRUE |
| *VAC14* | TRUE | FALSE | TRUE |
| *ZFYVE21* | TRUE | FALSE | TRUE |
| *APRT* | TRUE | TRUE | FALSE |
| *C19orf57* | TRUE | FALSE | TRUE |
| *TNFSF14* | TRUE | TRUE | TRUE |
| *SFXN4* | TRUE | TRUE | TRUE |
| *SFXN2* | TRUE | FALSE | TRUE |
| *SFXN1* | TRUE | TRUE | TRUE |
| *RWDD2B* | TRUE | FALSE | TRUE |
| *CCT5* | TRUE | FALSE | TRUE |
| *TIMM13* | TRUE | TRUE | TRUE |
| *USMG5* | TRUE | FALSE | FALSE |
| *KNTC1* | TRUE | TRUE | FALSE |
| *RNF20* | TRUE | FALSE | TRUE |
| *PCID2* | TRUE | TRUE | TRUE |

| | | | |
|---|---|---|---|
| *MOB1B* | TRUE | TRUE | TRUE |
| *APOL1* | TRUE | FALSE | TRUE |
| *ATN1* | TRUE | TRUE | FALSE |
| *TNIP2* | TRUE | FALSE | TRUE |
| *TNIP1* | TRUE | FALSE | TRUE |
| *RPP14* | TRUE | TRUE | TRUE |
| *GNPDA1* | TRUE | TRUE | TRUE |
| *USP31* | TRUE | TRUE | TRUE |
| *RAN* | TRUE | TRUE | TRUE |
| *ST7* | TRUE | TRUE | TRUE |
| *EXT2* | TRUE | TRUE | TRUE |
| *ALDH5A1* | TRUE | TRUE | TRUE |
| *SNX5* | TRUE | FALSE | TRUE |
| *TEFM* | TRUE | TRUE | TRUE |
| *LINC00665* | TRUE | TRUE | FALSE |
| *CCDC124* | TRUE | FALSE | FALSE |
| *KHSRP* | TRUE | FALSE | TRUE |
| *TIMELESS* | TRUE | TRUE | FALSE |
| *RNF219* | TRUE | FALSE | FALSE |
| *PKM* | TRUE | FALSE | FALSE |
| *DLAT* | TRUE | FALSE | TRUE |
| *UHRF1BP1L* | TRUE | FALSE | TRUE |
| *RNASEH2A* | TRUE | FALSE | TRUE |
| *DIEXF* | TRUE | FALSE | TRUE |
| *PGAM5* | TRUE | TRUE | TRUE |
| *C1QBP* | TRUE | TRUE | TRUE |
| *MED24* | TRUE | TRUE | FALSE |
| *LAMP3* | TRUE | TRUE | FALSE |
| *NAT10* | TRUE | TRUE | TRUE |
| *NELFCD* | TRUE | FALSE | FALSE |
| *RAD54L* | TRUE | TRUE | TRUE |
| *PSME3* | TRUE | FALSE | TRUE |
| *HMGA1* | TRUE | TRUE | TRUE |
| *FURIN* | TRUE | FALSE | TRUE |
| *EML2* | TRUE | TRUE | TRUE |
| *GMPS* | TRUE | TRUE | TRUE |
| *FAM98A* | TRUE | TRUE | TRUE |
| *NOP14* | TRUE | TRUE | TRUE |
| *C17orf51* | TRUE | TRUE | TRUE |
| *C17orf53* | TRUE | FALSE | FALSE |
| *TFDP1* | TRUE | FALSE | FALSE |
| *MIS18A* | TRUE | FALSE | TRUE |
| *BCL7A* | TRUE | FALSE | TRUE |
| *MRPL15* | TRUE | TRUE | TRUE |

| | | | |
|---|---|---|---|
| *LRP5* | TRUE | TRUE | FALSE |
| *LRP8* | TRUE | FALSE | TRUE |
| *MAP4* | TRUE | TRUE | FALSE |
| *MAP9* | TRUE | FALSE | TRUE |
| *THG1L* | TRUE | FALSE | TRUE |
| *GSS* | TRUE | FALSE | FALSE |
| *GSR* | TRUE | TRUE | TRUE |
| *LRPPRC* | TRUE | TRUE | TRUE |
| *SMIM15* | TRUE | TRUE | TRUE |
| *SERPINB8* | TRUE | FALSE | TRUE |
| *PTBP1* | TRUE | FALSE | TRUE |
| *HK2* | TRUE | FALSE | TRUE |
| *PABPC4* | TRUE | FALSE | FALSE |
| *EED* | TRUE | FALSE | TRUE |
| *MAPKAPK3* | TRUE | TRUE | TRUE |
| *PIK3R3* | TRUE | TRUE | TRUE |
| *PIK3R2* | TRUE | FALSE | TRUE |
| *CTNNBL1* | TRUE | FALSE | FALSE |
| *FAM111B* | TRUE | FALSE | TRUE |
| *ARMC9* | TRUE | TRUE | FALSE |
| *IDE* | TRUE | TRUE | TRUE |
| *TICAM1* | TRUE | TRUE | TRUE |
| *POLD1* | TRUE | TRUE | FALSE |
| *POLD2* | TRUE | TRUE | FALSE |
| *SAV1* | TRUE | FALSE | TRUE |
| *CEP152* | TRUE | TRUE | TRUE |
| *PDGFA* | TRUE | FALSE | TRUE |
| *LONP1* | TRUE | FALSE | TRUE |
| *ORC6* | TRUE | FALSE | TRUE |
| *ORC2* | TRUE | TRUE | TRUE |
| *ORC3* | TRUE | TRUE | TRUE |
| *ORC1* | TRUE | TRUE | TRUE |
| *RCC1* | TRUE | TRUE | TRUE |
| *ACTB* | TRUE | FALSE | TRUE |
| *SQLE* | TRUE | FALSE | TRUE |
| *NT5DC3* | TRUE | FALSE | TRUE |
| *KIAA1279* | TRUE | TRUE | TRUE |
| *SERBP1* | TRUE | TRUE | TRUE |
| *NDUFAF6* | TRUE | FALSE | TRUE |
| *GDAP1* | TRUE | FALSE | TRUE |
| *PRKAR1B* | TRUE | TRUE | TRUE |
| *NOL6* | TRUE | TRUE | TRUE |
| *C19orf10* | TRUE | TRUE | TRUE |
| *CDC45* | TRUE | FALSE | TRUE |

| | | | |
|---|---|---|---|
| *DANCR* | TRUE | FALSE | TRUE |
| *CHAF1A* | TRUE | TRUE | TRUE |
| *CHAF1B* | TRUE | TRUE | TRUE |
| *CDCA7* | TRUE | FALSE | TRUE |
| *CDCA4* | TRUE | TRUE | TRUE |
| *CDCA5* | TRUE | TRUE | FALSE |
| *TIMM50* | TRUE | FALSE | TRUE |
| *ALKBH3* | TRUE | TRUE | TRUE |
| *SPRED2* | TRUE | FALSE | TRUE |
| *ZMPSTE24* | TRUE | FALSE | TRUE |
| *MRPL37* | TRUE | TRUE | TRUE |
| *MRPL35* | TRUE | TRUE | TRUE |
| *MRPL30* | TRUE | FALSE | TRUE |
| *MRPL38* | TRUE | FALSE | TRUE |
| *MRPL39* | TRUE | FALSE | TRUE |
| *THOC5* | TRUE | TRUE | TRUE |
| *WHSC1* | TRUE | FALSE | TRUE |
| *RECQL4* | TRUE | FALSE | FALSE |
| *COPS6* | TRUE | FALSE | FALSE |
| *ZNF598* | TRUE | FALSE | TRUE |
| *TKT* | TRUE | FALSE | FALSE |
| *NLN* | TRUE | TRUE | TRUE |
| *NUDT1* | TRUE | FALSE | FALSE |
| *TK1* | TRUE | FALSE | FALSE |
| *CELSR3* | TRUE | TRUE | TRUE |
| *LRRC32* | TRUE | TRUE | TRUE |
| *MFN2* | TRUE | FALSE | TRUE |
| *MAD2L2* | TRUE | TRUE | TRUE |
| *UQCRH* | TRUE | FALSE | FALSE |
| *GPS1* | TRUE | FALSE | FALSE |
| *GLO1* | TRUE | TRUE | TRUE |
| *COIL* | TRUE | TRUE | FALSE |
| *SLC15A4* | TRUE | TRUE | TRUE |
| *RRAS2* | TRUE | FALSE | TRUE |
| *SLC31A2* | TRUE | TRUE | TRUE |
| *CHEK1* | TRUE | TRUE | TRUE |
| *GINS3* | TRUE | TRUE | TRUE |
| *GINS4* | TRUE | TRUE | TRUE |
| *C19orf48* | TRUE | FALSE | TRUE |
| *CHCHD10* | TRUE | FALSE | FALSE |
| *ZNF100* | TRUE | TRUE | TRUE |
| *GUCD1* | TRUE | TRUE | TRUE |
| *TRAFD1* | TRUE | FALSE | TRUE |
| *AGPAT9* | TRUE | FALSE | TRUE |

| | | | |
|---|---|---|---|
| *PEX10* | TRUE | FALSE | TRUE |
| *ODC1* | TRUE | FALSE | TRUE |
| *PPP1CC* | TRUE | FALSE | TRUE |
| *WBP4* | TRUE | TRUE | TRUE |
| *PPP1R16A* | TRUE | FALSE | FALSE |
| *TMA16* | TRUE | FALSE | TRUE |
| *IL1R1* | TRUE | TRUE | TRUE |
| *MRPL11* | TRUE | FALSE | FALSE |
| *MRPL12* | TRUE | FALSE | TRUE |
| *CDC25A* | TRUE | TRUE | TRUE |
| *RNF40* | TRUE | TRUE | TRUE |
| *DCUN1D1* | TRUE | FALSE | TRUE |
| *DCUN1D3* | TRUE | TRUE | TRUE |
| *ZNF827* | TRUE | TRUE | TRUE |
| *KDSR* | TRUE | FALSE | TRUE |
| *POLE* | TRUE | TRUE | FALSE |
| *AIFM2* | TRUE | TRUE | TRUE |
| *SACS* | TRUE | TRUE | TRUE |
| *NBN* | TRUE | TRUE | TRUE |
| *DNPEP* | TRUE | FALSE | TRUE |
| *SIAH2* | TRUE | TRUE | TRUE |
| *CLPTM1L* | TRUE | FALSE | FALSE |
| *PHLPP2* | TRUE | FALSE | TRUE |
| *DAPP1* | TRUE | FALSE | TRUE |
| *HSPBP1* | TRUE | FALSE | TRUE |
| *GSTP1* | TRUE | FALSE | FALSE |
| *RMDN3* | TRUE | FALSE | TRUE |
| *LRRC59* | TRUE | FALSE | FALSE |
| *ERRFI1* | TRUE | FALSE | TRUE |
| *SMG9* | TRUE | TRUE | TRUE |
| *GRWD1* | TRUE | FALSE | TRUE |
| *IFNG* | TRUE | TRUE | FALSE |
| *IFRD2* | TRUE | FALSE | TRUE |
| *TEX2* | TRUE | TRUE | FALSE |
| *STARD7* | TRUE | FALSE | TRUE |
| *IMPAD1* | TRUE | FALSE | TRUE |
| *TOMM6* | TRUE | FALSE | FALSE |
| *GEMIN4* | TRUE | TRUE | TRUE |
| *LARP1B* | TRUE | FALSE | TRUE |
| *LARS2* | TRUE | TRUE | TRUE |
| *DDX10* | TRUE | TRUE | TRUE |
| *DDX11* | TRUE | TRUE | TRUE |
| *GJB6* | TRUE | FALSE | TRUE |
| *PSMC2* | TRUE | FALSE | TRUE |

| | | | |
|---|---|---|---|
| *PSMC3* | TRUE | FALSE | TRUE |
| *TRIP10* | TRUE | TRUE | TRUE |
| *FN3KRP* | TRUE | TRUE | TRUE |
| *GRHPR* | TRUE | TRUE | FALSE |
| *EIF3B* | TRUE | FALSE | TRUE |
| *NOL10* | TRUE | TRUE | TRUE |
| *NOL12* | TRUE | FALSE | TRUE |
| *NME6* | TRUE | TRUE | TRUE |
| *HCCS* | TRUE | TRUE | TRUE |
| *GTF2E2* | TRUE | TRUE | TRUE |
| *PPP1R14B* | TRUE | TRUE | FALSE |
| *ALKBH8* | TRUE | TRUE | TRUE |
| *VPS53* | TRUE | TRUE | TRUE |
| *ALKBH2* | TRUE | TRUE | TRUE |
| *DBF4B* | TRUE | TRUE | TRUE |
| *PHB2* | TRUE | FALSE | TRUE |
| *MSH3* | TRUE | TRUE | TRUE |
| *MSH6* | TRUE | FALSE | TRUE |
| *C16orf45* | TRUE | FALSE | TRUE |
| *PACSIN3* | TRUE | FALSE | FALSE |
| *PM20D2* | TRUE | TRUE | TRUE |
| *SAE1* | TRUE | TRUE | TRUE |
| *NT5C* | TRUE | FALSE | FALSE |
| *TOMM5* | TRUE | FALSE | TRUE |
| *CHERP* | TRUE | FALSE | TRUE |
| *NME4* | TRUE | TRUE | FALSE |
| *CLSPN* | TRUE | FALSE | TRUE |
| *CLUH* | TRUE | FALSE | FALSE |
| *TBCE* | TRUE | TRUE | TRUE |
| *UPF2* | TRUE | TRUE | TRUE |
| *PHTF2* | TRUE | TRUE | TRUE |
| *LINC00116* | TRUE | FALSE | TRUE |
| *COMT* | TRUE | FALSE | FALSE |
| *ARL6* | TRUE | TRUE | TRUE |
| *COLGALT1* | TRUE | FALSE | TRUE |
| *TTF2* | TRUE | TRUE | TRUE |
| *TTF1* | TRUE | TRUE | TRUE |
| *PINX1* | TRUE | TRUE | TRUE |
| *AEBP1* | TRUE | TRUE | FALSE |
| *LTA4H* | TRUE | TRUE | FALSE |
| *ANXA7* | TRUE | FALSE | TRUE |
| *MCM6* | TRUE | FALSE | TRUE |
| *MCM5* | TRUE | TRUE | FALSE |
| *MCM4* | TRUE | FALSE | TRUE |

| | | | |
|---|---|---|---|
| *MCM3* | TRUE | FALSE | TRUE |
| *DUSP18* | TRUE | TRUE | FALSE |
| *MCM9* | TRUE | FALSE | TRUE |
| *ZNF142* | TRUE | TRUE | TRUE |
| *MBIP* | TRUE | FALSE | TRUE |
| *DDX31* | TRUE | FALSE | FALSE |
| *COX8A* | TRUE | FALSE | FALSE |
| *PFAS* | TRUE | TRUE | TRUE |
| *C20orf27* | TRUE | TRUE | TRUE |
| *KCNN4* | TRUE | TRUE | TRUE |
| *NIT2* | TRUE | TRUE | TRUE |
| *MIS12* | TRUE | TRUE | TRUE |
| *SLC25A43* | TRUE | TRUE | FALSE |
| *PDZD8* | TRUE | FALSE | TRUE |
| *TNPO2* | TRUE | TRUE | TRUE |
| *TNPO3* | TRUE | FALSE | TRUE |
| *MAPK6* | TRUE | FALSE | TRUE |
| *FDX1L* | TRUE | TRUE | TRUE |
| *KCTD6* | TRUE | FALSE | TRUE |
| *C1orf109* | TRUE | TRUE | TRUE |
| *HCFC1* | TRUE | FALSE | TRUE |
| *FOXRED2* | TRUE | FALSE | TRUE |
| *FOXRED1* | TRUE | TRUE | FALSE |
| *ANP32B* | TRUE | FALSE | TRUE |
| *RPF2* | TRUE | TRUE | TRUE |
| *ZNF689* | TRUE | FALSE | TRUE |
| *GRSF1* | TRUE | TRUE | TRUE |
| *IQCG* | TRUE | TRUE | TRUE |
| *GPR114* | TRUE | TRUE | TRUE |
| *GPAA1* | TRUE | TRUE | FALSE |
| *NCAPD3* | TRUE | TRUE | TRUE |
| *BLM* | TRUE | TRUE | TRUE |
| *MMACHC* | TRUE | FALSE | TRUE |
| *ATXN2* | TRUE | TRUE | TRUE |
| *RIOK1* | TRUE | FALSE | TRUE |
| *LTV1* | TRUE | FALSE | TRUE |
| *MDN1* | TRUE | TRUE | FALSE |
| *NHEJ1* | TRUE | TRUE | TRUE |
| *CSF1* | TRUE | TRUE | TRUE |
| *DDX55* | TRUE | FALSE | TRUE |
| *BUB1* | TRUE | TRUE | TRUE |
| *PLA2G15* | TRUE | TRUE | TRUE |
| *TMEM138* | TRUE | TRUE | TRUE |
| *SPRY1* | TRUE | FALSE | FALSE |

| | | | |
|---|---|---|---|
| *COASY* | TRUE | FALSE | TRUE |
| *CDKL1* | TRUE | TRUE | TRUE |
| *MCMBP* | TRUE | FALSE | TRUE |
| *CKAP2L* | TRUE | TRUE | TRUE |
| *NDUFS1* | TRUE | FALSE | TRUE |
| *NDUFS4* | TRUE | FALSE | TRUE |
| *KRI1* | TRUE | TRUE | TRUE |
| *FIBP* | TRUE | FALSE | FALSE |
| *CSTF2* | TRUE | TRUE | TRUE |
| *PCCB* | TRUE | FALSE | TRUE |
| *LIMA1* | TRUE | FALSE | TRUE |
| *PNPO* | TRUE | TRUE | TRUE |
| *HRAS* | TRUE | TRUE | FALSE |
| *ZNF132* | TRUE | FALSE | TRUE |
| *C16orf87* | TRUE | FALSE | TRUE |
| *GPHN* | TRUE | TRUE | TRUE |
| *AIMP2* | TRUE | TRUE | TRUE |
| *VWA9* | TRUE | FALSE | TRUE |
| *CASP2* | TRUE | FALSE | TRUE |
| *CASP3* | TRUE | FALSE | TRUE |
| *LSG1* | TRUE | TRUE | TRUE |
| *PICALM* | TRUE | TRUE | FALSE |
| *ANAPC13* | TRUE | TRUE | TRUE |
| *MYC* | TRUE | TRUE | FALSE |
| *MYB* | TRUE | TRUE | FALSE |
| *ENTPD7* | TRUE | TRUE | TRUE |
| *SNAPC4* | TRUE | TRUE | TRUE |
| *SLC4A2* | TRUE | FALSE | FALSE |
| *NPEPPS* | TRUE | TRUE | TRUE |
| *KIF3B* | TRUE | TRUE | TRUE |
| *VDR* | TRUE | TRUE | TRUE |
| *SNUPN* | TRUE | TRUE | TRUE |
| *PDSS1* | TRUE | FALSE | TRUE |
| *STOML2* | TRUE | TRUE | TRUE |
| *LEPRE1* | TRUE | TRUE | TRUE |
| *CDKN3* | TRUE | FALSE | TRUE |
| *LSM10* | TRUE | FALSE | TRUE |
| *LSM11* | TRUE | TRUE | TRUE |
| *SGPL1* | TRUE | TRUE | TRUE |
| *NOB1* | TRUE | FALSE | TRUE |
| *IL21-AS1* | TRUE | TRUE | TRUE |
| *IRAK1* | TRUE | FALSE | TRUE |
| *WDR5* | TRUE | FALSE | TRUE |
| *WDR4* | TRUE | TRUE | TRUE |

| | | | |
|---|---|---|---|
| *POU2AF1* | TRUE | TRUE | TRUE |
| *CCND1* | TRUE | FALSE | TRUE |
| *MSH2* | TRUE | TRUE | TRUE |
| *SERPINE2* | TRUE | FALSE | FALSE |
| *CHRAC1* | TRUE | FALSE | TRUE |
| *PGM2* | TRUE | FALSE | TRUE |
| *ZDHHC6* | TRUE | TRUE | TRUE |
| *C8orf37* | TRUE | TRUE | TRUE |
| *KLHL26* | TRUE | TRUE | TRUE |
| *SMS* | TRUE | FALSE | TRUE |
| *POLK* | TRUE | TRUE | TRUE |
| *NDUFV3* | TRUE | TRUE | TRUE |
| *NDUFV1* | TRUE | FALSE | FALSE |
| *SIGMAR1* | TRUE | TRUE | TRUE |
| *PRC1* | TRUE | FALSE | TRUE |
| *RPTOR* | TRUE | FALSE | TRUE |
| *ZNF649* | TRUE | FALSE | TRUE |
| *KPNB1* | TRUE | TRUE | TRUE |
| *HUWE1* | TRUE | TRUE | FALSE |
| *UTP14A* | TRUE | TRUE | TRUE |
| *HTATIP2* | TRUE | TRUE | TRUE |
| *SLC29A1* | TRUE | TRUE | FALSE |
| *ERO1L* | TRUE | FALSE | TRUE |
| *CD70* | TRUE | FALSE | TRUE |
| *CD72* | TRUE | TRUE | FALSE |
| *RFC5* | TRUE | TRUE | TRUE |
| *RFC4* | TRUE | FALSE | TRUE |
| *RFC3* | TRUE | TRUE | TRUE |
| *RFC2* | TRUE | TRUE | TRUE |
| *CBX3* | TRUE | TRUE | TRUE |
| *FASTKD1* | TRUE | TRUE | TRUE |
| *PSEN2* | TRUE | FALSE | TRUE |
| *KIF1B* | TRUE | TRUE | TRUE |
| *KIF1C* | TRUE | FALSE | TRUE |
| *AATF* | TRUE | TRUE | TRUE |
| *TPI1* | TRUE | TRUE | FALSE |
| *ERCC1* | TRUE | TRUE | TRUE |
| *ERCC2* | TRUE | TRUE | FALSE |
| *MRPS15* | TRUE | TRUE | FALSE |
| *PFKP* | TRUE | FALSE | FALSE |
| *SUZ12* | TRUE | TRUE | TRUE |
| *WARS* | TRUE | TRUE | FALSE |
| *SEH1L* | TRUE | TRUE | TRUE |
| *NCDN* | TRUE | FALSE | TRUE |

| | | | |
|---|---|---|---|
| *SLC25A22* | TRUE | FALSE | TRUE |
| *SLC25A20* | TRUE | TRUE | TRUE |
| *CARM1* | TRUE | TRUE | TRUE |
| *RPUSD4* | TRUE | FALSE | TRUE |
| *RPAP1* | TRUE | TRUE | TRUE |
| *PSTPIP2* | TRUE | TRUE | TRUE |
| *POFUT1* | TRUE | TRUE | TRUE |
| *SNX10* | TRUE | TRUE | TRUE |
| *ICAM1* | TRUE | FALSE | TRUE |
| *SMTN* | TRUE | FALSE | FALSE |
| *TYW3* | TRUE | TRUE | TRUE |
| *ARPC5L* | TRUE | FALSE | TRUE |
| *MAP4K5* | TRUE | FALSE | TRUE |
| *MIR17HG* | TRUE | FALSE | FALSE |
| *NCKIPSD* | TRUE | FALSE | TRUE |
| *PDCD2L* | TRUE | FALSE | TRUE |
| *SUV39H2* | TRUE | FALSE | TRUE |
| *CYC1* | TRUE | FALSE | TRUE |
| *BCL2L1* | TRUE | TRUE | TRUE |
| *SCO1* | TRUE | FALSE | TRUE |
| *SPINT1* | TRUE | FALSE | TRUE |
| *YBEY* | TRUE | TRUE | TRUE |
| *C7orf49* | TRUE | FALSE | TRUE |
| *TDP1* | TRUE | TRUE | TRUE |
| *L3MBTL2* | TRUE | TRUE | TRUE |
| *C9orf3* | TRUE | FALSE | TRUE |
| *TMED2* | TRUE | FALSE | TRUE |
| *ARPP19* | TRUE | TRUE | TRUE |
| *HNRNPR* | TRUE | TRUE | TRUE |
| *HNRNPF* | TRUE | TRUE | TRUE |
| *ZBTB45* | TRUE | TRUE | TRUE |
| *PDIA6* | TRUE | FALSE | TRUE |
| *ARMC6* | TRUE | TRUE | TRUE |
| *CD226* | TRUE | TRUE | TRUE |
| *ZDHHC18* | TRUE | TRUE | TRUE |
| *CEP83* | TRUE | TRUE | TRUE |
| *CEP85* | TRUE | TRUE | TRUE |
| *PTRH2* | TRUE | TRUE | FALSE |
| *NTHL1* | TRUE | FALSE | FALSE |
| *GTF3C6* | TRUE | FALSE | TRUE |
| *XPO1* | TRUE | TRUE | TRUE |
| *XPO5* | TRUE | TRUE | TRUE |
| *XPO7* | TRUE | TRUE | TRUE |
| *GAR1* | TRUE | TRUE | TRUE |

| | | | |
|---|---|---|---|
| *XPOT* | TRUE | TRUE | TRUE |
| *GART* | TRUE | TRUE | TRUE |
| *ATAD5* | TRUE | TRUE | TRUE |
| *ATAD2* | TRUE | FALSE | TRUE |
| *TMEM192* | TRUE | FALSE | TRUE |
| *MTIF2* | TRUE | FALSE | FALSE |
| *PRIM1* | TRUE | TRUE | TRUE |
| *TTC27* | TRUE | TRUE | TRUE |
| *ENOPH1* | TRUE | TRUE | TRUE |
| *SRPK1* | TRUE | TRUE | TRUE |
| *LARP1* | TRUE | TRUE | TRUE |
| *NOLC1* | TRUE | TRUE | TRUE |
| *THAP3* | TRUE | TRUE | TRUE |
| *CHID1* | TRUE | FALSE | FALSE |
| *PES1* | TRUE | TRUE | TRUE |
| *ASB2* | TRUE | TRUE | TRUE |
| *ATP5SL* | TRUE | FALSE | TRUE |
| *TBL3* | TRUE | FALSE | FALSE |
| *DBI* | TRUE | FALSE | FALSE |
| *MED9* | TRUE | FALSE | TRUE |
| *SDF2L1* | TRUE | TRUE | TRUE |
| *PPP5C* | TRUE | TRUE | TRUE |
| *NUTF2* | TRUE | FALSE | FALSE |
| *CHPT1* | TRUE | TRUE | FALSE |
| *UTP15* | TRUE | TRUE | TRUE |
| *DPAGT1* | TRUE | TRUE | FALSE |
| *SEPHS1* | TRUE | TRUE | TRUE |
| *TET2* | TRUE | FALSE | TRUE |
| *BUD13* | TRUE | FALSE | TRUE |
| *PRDX4* | TRUE | FALSE | TRUE |
| *PRDX3* | TRUE | TRUE | TRUE |
| *PRDX2* | TRUE | FALSE | FALSE |
| *OTUB1* | TRUE | FALSE | TRUE |
| *NPTN* | TRUE | TRUE | TRUE |
| *DCAF4* | TRUE | TRUE | TRUE |
| *CD38* | TRUE | TRUE | TRUE |
| *POLR3D* | TRUE | TRUE | TRUE |
| *POLR3A* | TRUE | TRUE | TRUE |
| *WDR62* | TRUE | FALSE | TRUE |
| *PRMT3* | TRUE | TRUE | TRUE |
| *TONSL* | TRUE | TRUE | FALSE |
| *HMGCR* | TRUE | FALSE | TRUE |
| *MTPAP* | TRUE | FALSE | TRUE |
| *PPARGC1B* | TRUE | FALSE | TRUE |

| | | | |
|---|---|---|---|
| *ACAD9* | TRUE | FALSE | TRUE |
| *VDAC3* | TRUE | FALSE | TRUE |
| *TMEM201* | TRUE | FALSE | TRUE |
| *TMEM209* | TRUE | TRUE | TRUE |
| *HMGCS1* | TRUE | TRUE | TRUE |
| *GALM* | TRUE | TRUE | TRUE |
| *PRICKLE3* | TRUE | FALSE | TRUE |
| *ATG7* | TRUE | FALSE | FALSE |
| *VEZT* | TRUE | FALSE | TRUE |
| *ABCB6* | TRUE | TRUE | FALSE |
| *FEN1* | TRUE | FALSE | TRUE |
| *SCD* | TRUE | FALSE | TRUE |
| *STRIP2* | TRUE | TRUE | TRUE |
| *TMEM64* | TRUE | TRUE | TRUE |
| *TMEM69* | TRUE | FALSE | TRUE |
| *OGFOD1* | TRUE | TRUE | TRUE |
| *ZNF629* | TRUE | TRUE | TRUE |
| *NEURL4* | TRUE | TRUE | TRUE |
| *BRE* | TRUE | FALSE | FALSE |
| *NCS1* | TRUE | FALSE | TRUE |
| *TRAF2* | TRUE | TRUE | TRUE |
| *CPOX* | TRUE | TRUE | TRUE |
| *DKC1* | TRUE | TRUE | TRUE |
| *COMMD1* | TRUE | TRUE | FALSE |
| *RUVBL2* | TRUE | FALSE | TRUE |
| *COMMD4* | TRUE | FALSE | FALSE |
| *COMMD5* | TRUE | TRUE | FALSE |
| *ZMYM4* | TRUE | TRUE | FALSE |
| *CAMK2D* | TRUE | TRUE | TRUE |
| *DEPDC1B* | TRUE | TRUE | TRUE |
| *ASCC2* | TRUE | TRUE | TRUE |
| *POLR1B* | TRUE | TRUE | TRUE |
| *POLR1E* | TRUE | FALSE | TRUE |
| *WDR41* | TRUE | TRUE | TRUE |
| *DUS1L* | TRUE | FALSE | FALSE |
| *WDR43* | TRUE | FALSE | TRUE |
| *JAK2* | TRUE | TRUE | TRUE |
| *GTF2I* | TRUE | TRUE | TRUE |
| *PRKDC* | TRUE | TRUE | TRUE |
| *DYRK3* | TRUE | FALSE | TRUE |
| *NR2F6* | TRUE | TRUE | TRUE |
| *POLR2K* | TRUE | FALSE | TRUE |
| *MCAT* | TRUE | FALSE | TRUE |
| *MCM2* | TRUE | FALSE | TRUE |

| | | | |
|---|---|---|---|
| *APOBEC3C* | TRUE | FALSE | TRUE |
| *CTTN* | TRUE | FALSE | TRUE |
| *EIF2S1* | TRUE | FALSE | TRUE |
| *GPI* | TRUE | TRUE | FALSE |
| *SLC1A5* | TRUE | FALSE | TRUE |
| *DCTPP1* | TRUE | FALSE | TRUE |
| *GNL2* | TRUE | FALSE | TRUE |
| *RARG* | TRUE | TRUE | TRUE |
| *RARS* | TRUE | TRUE | TRUE |
| *BAZ1B* | TRUE | TRUE | TRUE |
| *SLC2A1* | TRUE | TRUE | TRUE |
| *ECHS1* | TRUE | FALSE | FALSE |
| *PLAGL2* | TRUE | TRUE | TRUE |
| *EXO1* | TRUE | TRUE | TRUE |
| *ATRIP* | TRUE | TRUE | TRUE |
| *PARP2* | TRUE | TRUE | TRUE |
| *ENG* | TRUE | TRUE | FALSE |
| *MREG* | TRUE | TRUE | TRUE |
| *AGMAT* | TRUE | FALSE | TRUE |
| *TRNAU1AP* | TRUE | FALSE | TRUE |
| *FLT1* | TRUE | TRUE | TRUE |
| *SET* | TRUE | FALSE | TRUE |
| *RCL1* | TRUE | TRUE | TRUE |
| *CHAMP1* | TRUE | TRUE | TRUE |
| *PNPT1* | TRUE | TRUE | TRUE |
| *SF3B3* | TRUE | FALSE | TRUE |
| *NSMCE4A* | TRUE | FALSE | TRUE |
| *ELAC1* | TRUE | TRUE | TRUE |
| *ELAC2* | TRUE | TRUE | TRUE |
| *ADCK1* | TRUE | TRUE | FALSE |
| *DNAJC16* | TRUE | TRUE | TRUE |
| *DNAJC17* | TRUE | FALSE | FALSE |
| *DNAJC10* | TRUE | TRUE | TRUE |
| *DNAJC11* | TRUE | TRUE | TRUE |
| *ASF1B* | TRUE | TRUE | TRUE |
| *SUPT16H* | TRUE | TRUE | TRUE |
| *LMAN1* | TRUE | TRUE | TRUE |
| *MLX* | TRUE | FALSE | TRUE |
| *ITPA* | TRUE | TRUE | FALSE |
| *ACTG1* | TRUE | FALSE | TRUE |
| *PRPF31* | TRUE | FALSE | FALSE |
| *SURF4* | TRUE | FALSE | TRUE |
| *PRSS23* | TRUE | TRUE | FALSE |
| *SURF6* | TRUE | TRUE | TRUE |

| | | | |
|---|---|---|---|
| *ZNF485* | TRUE | TRUE | FALSE |
| *TP53* | TRUE | FALSE | TRUE |
| *SHQ1* | TRUE | TRUE | TRUE |
| *DUS3L* | TRUE | FALSE | TRUE |
| *C5orf30* | TRUE | TRUE | TRUE |
| *FARSA* | TRUE | FALSE | TRUE |
| *DPP9* | TRUE | TRUE | TRUE |
| *DPP3* | TRUE | FALSE | FALSE |
| *KNOP1* | TRUE | FALSE | FALSE |
| *PHF5A* | TRUE | TRUE | TRUE |
| *NOC3L* | TRUE | TRUE | TRUE |
| *TRMT61A* | TRUE | FALSE | TRUE |
| *GOT2* | TRUE | TRUE | TRUE |
| *RPP40* | TRUE | FALSE | TRUE |
| *QTRT1* | TRUE | FALSE | FALSE |
| *SMIM20* | TRUE | TRUE | TRUE |
| *ADRM1* | TRUE | FALSE | TRUE |
| *ZNF562* | TRUE | TRUE | TRUE |
| *TRAF4* | TRUE | FALSE | TRUE |
| *UBE3C* | TRUE | TRUE | TRUE |
| *TOP2A* | TRUE | TRUE | TRUE |
| *KIAA0368* | TRUE | TRUE | TRUE |
| *C16orf91* | TRUE | TRUE | FALSE |
| *SDC4* | TRUE | FALSE | TRUE |
| *ELOVL6* | TRUE | TRUE | TRUE |
| *HTT* | TRUE | FALSE | FALSE |
| *ELOVL1* | TRUE | FALSE | TRUE |
| *NAA25* | TRUE | TRUE | TRUE |
| *KIAA1586* | TRUE | TRUE | TRUE |
| *DUSP5* | TRUE | FALSE | FALSE |
| *DUSP4* | TRUE | FALSE | TRUE |
| *LRRC8B* | TRUE | TRUE | FALSE |
| *QDPR* | TRUE | FALSE | TRUE |
| *RDX* | TRUE | TRUE | TRUE |
| *XYLB* | TRUE | TRUE | FALSE |
| *NEK6* | TRUE | FALSE | TRUE |
| *TMEM106C* | TRUE | TRUE | TRUE |
| *NPM1* | TRUE | TRUE | FALSE |
| *PEMT* | TRUE | FALSE | FALSE |
| *DDX54* | TRUE | FALSE | TRUE |
| *BYSL* | TRUE | FALSE | TRUE |
| *SQRDL* | TRUE | FALSE | TRUE |
| *SCCPDH* | TRUE | TRUE | FALSE |
| *STAMBPL1* | TRUE | TRUE | TRUE |

| | | | |
|---|---|---|---|
| *CNDP2* | TRUE | TRUE | TRUE |
| *TTI2* | TRUE | FALSE | TRUE |
| *TTI1* | TRUE | TRUE | TRUE |
| *TXNRD1* | TRUE | FALSE | TRUE |
| *QSOX2* | TRUE | FALSE | TRUE |
| *PDS5B* | TRUE | FALSE | TRUE |
| *TADA2A* | TRUE | TRUE | TRUE |
| *NR2C2AP* | TRUE | FALSE | TRUE |
| *PAICS* | TRUE | TRUE | TRUE |
| *VEGFA* | TRUE | TRUE | FALSE |
| *EMC1* | TRUE | FALSE | TRUE |
| *OTUD4* | TRUE | FALSE | TRUE |
| *ARID5B* | TRUE | FALSE | FALSE |
| *ACER3* | TRUE | FALSE | TRUE |
| *EXOC2* | TRUE | TRUE | TRUE |
| *EXOC3* | TRUE | TRUE | TRUE |
| *ANK3* | TRUE | FALSE | TRUE |
| *C9orf91* | TRUE | TRUE | TRUE |
| *RAD18* | TRUE | FALSE | TRUE |
| *CENPO* | TRUE | FALSE | TRUE |
| *CENPN* | TRUE | FALSE | TRUE |
| *CENPI* | TRUE | TRUE | FALSE |
| *CENPH* | TRUE | TRUE | FALSE |
| *CENPV* | TRUE | FALSE | TRUE |
| *CENPU* | TRUE | FALSE | TRUE |
| *SLC9B2* | TRUE | FALSE | TRUE |
| *TRIB1* | TRUE | FALSE | FALSE |
| *DHX30* | TRUE | TRUE | TRUE |
| *DHX33* | TRUE | TRUE | TRUE |
| *DHX34* | TRUE | FALSE | TRUE |
| *DHX35* | TRUE | TRUE | TRUE |
| *DHX37* | TRUE | FALSE | TRUE |
| *PRIM2* | TRUE | FALSE | FALSE |
| *RACGAP1* | TRUE | TRUE | TRUE |
| *PUS7* | TRUE | TRUE | TRUE |
| *TANGO6* | TRUE | FALSE | TRUE |
| *KEAP1* | TRUE | FALSE | TRUE |
| *ZBTB8OS* | TRUE | FALSE | FALSE |
| *POLA2* | TRUE | TRUE | TRUE |
| *PHF13* | TRUE | FALSE | FALSE |
| *EZH2* | TRUE | FALSE | TRUE |
| *RAVER1* | TRUE | FALSE | TRUE |
| *CASC5* | TRUE | FALSE | TRUE |
| *MYEOV2* | TRUE | FALSE | FALSE |

| | | | |
|---|---|---|---|
| *BOLA3* | TRUE | FALSE | TRUE |
| *ZW10* | TRUE | FALSE | TRUE |
| *LINC00944* | TRUE | TRUE | TRUE |
| *MRRF* | TRUE | TRUE | TRUE |
| *PHB* | TRUE | FALSE | FALSE |
| *SNHG1* | TRUE | FALSE | FALSE |
| *MED22* | TRUE | TRUE | TRUE |
| *PARP1* | TRUE | TRUE | TRUE |
| *PTPDC1* | TRUE | TRUE | FALSE |
| *MELK* | TRUE | TRUE | TRUE |
| *DSN1* | TRUE | FALSE | TRUE |
| *SLC10A7* | TRUE | TRUE | TRUE |
| *THOP1* | TRUE | FALSE | TRUE |
| *ABCF2* | TRUE | TRUE | TRUE |
| *HMGXB3* | TRUE | TRUE | TRUE |
| *HMGXB4* | TRUE | TRUE | TRUE |
| *DNA2* | TRUE | TRUE | TRUE |
| *GLRX5* | TRUE | FALSE | TRUE |
| *GLRX3* | TRUE | FALSE | TRUE |
| *C19orf44* | TRUE | TRUE | TRUE |
| *C19orf47* | TRUE | FALSE | TRUE |
| *KLHL8* | TRUE | FALSE | FALSE |
| *CIRH1A* | TRUE | TRUE | TRUE |
| *TCTN3* | TRUE | TRUE | TRUE |
| *GRAMD3* | TRUE | TRUE | TRUE |
| *PCTP* | TRUE | FALSE | TRUE |
| *GRAMD4* | TRUE | TRUE | TRUE |
| *HN1L* | TRUE | TRUE | TRUE |
| *TIAM1* | TRUE | FALSE | TRUE |
| *ZNF749* | TRUE | TRUE | TRUE |
| *DAPK3* | TRUE | TRUE | TRUE |
| *RFXANK* | TRUE | FALSE | FALSE |
| *PIM3* | TRUE | FALSE | TRUE |
| *FKBP3* | TRUE | TRUE | TRUE |
| *SYMPK* | TRUE | TRUE | FALSE |
| *APOBEC3G* | TRUE | TRUE | TRUE |
| *DTL* | TRUE | TRUE | TRUE |
| *SPOPL* | TRUE | FALSE | TRUE |
| *PDRG1* | TRUE | FALSE | TRUE |
| *MYBBP1A* | TRUE | TRUE | TRUE |
| *RCAN1* | TRUE | FALSE | TRUE |
| *LETM1* | TRUE | FALSE | TRUE |
| *BRIX1* | TRUE | FALSE | TRUE |
| *ICA1L* | TRUE | FALSE | FALSE |

| | | | |
|---|---|---|---|
| *RBM19* | TRUE | TRUE | TRUE |
| *MVD* | TRUE | TRUE | TRUE |
| *GRAMD1B* | TRUE | TRUE | TRUE |
| *TUBGCP3* | TRUE | TRUE | TRUE |
| *TUBGCP4* | TRUE | FALSE | TRUE |
| *TUBGCP5* | TRUE | TRUE | TRUE |
| *PSMD1* | TRUE | TRUE | TRUE |
| *PSMD2* | TRUE | TRUE | FALSE |
| *ZNF16* | TRUE | FALSE | FALSE |
| *MRPL21* | TRUE | FALSE | TRUE |
| *RABGGTB* | TRUE | FALSE | TRUE |
| *RABGGTA* | TRUE | FALSE | FALSE |
| *NFE2L1* | TRUE | FALSE | TRUE |
| *PLBD2* | TRUE | FALSE | FALSE |
| *YEATS2* | TRUE | TRUE | FALSE |
| *YIPF1* | TRUE | FALSE | TRUE |
| *FTSJ1* | TRUE | TRUE | TRUE |
| *RAD54L2* | TRUE | TRUE | TRUE |
| *MMS22L* | TRUE | TRUE | TRUE |
| *IBTK* | TRUE | TRUE | TRUE |
| *TMEM161A* | TRUE | TRUE | FALSE |
| *CDT1* | TRUE | FALSE | FALSE |
| *TNFSF8* | TRUE | FALSE | TRUE |
| *GMPPA* | TRUE | FALSE | FALSE |
| *SND1* | TRUE | FALSE | FALSE |
| *RAD50* | TRUE | TRUE | TRUE |
| *USP5* | TRUE | FALSE | TRUE |
| *FAM86C1* | TRUE | TRUE | TRUE |
| *XRCC5* | TRUE | TRUE | TRUE |
| *INTS7* | TRUE | TRUE | TRUE |
| *CTNNA1* | TRUE | TRUE | FALSE |
| *TOMM40* | TRUE | FALSE | TRUE |
| *CHSY1* | TRUE | TRUE | TRUE |
| *FUOM* | TRUE | FALSE | FALSE |
| *MPP6* | TRUE | TRUE | TRUE |
| *PRPSAP2* | TRUE | TRUE | TRUE |
| *MAN1A1* | TRUE | TRUE | TRUE |
| *ATIC* | TRUE | TRUE | TRUE |
| *PPRC1* | TRUE | FALSE | TRUE |
| *TWSG1* | TRUE | FALSE | TRUE |
| *CCDC137* | TRUE | FALSE | TRUE |
| *CCDC138* | TRUE | TRUE | TRUE |
| *TIMM22* | TRUE | FALSE | TRUE |
| *STK39* | TRUE | TRUE | TRUE |

| | | | |
|---|---|---|---|
| *C12orf10* | TRUE | FALSE | TRUE |
| *CD2* | TRUE | FALSE | TRUE |
| *DNAAF2* | TRUE | TRUE | TRUE |
| *STAT1* | TRUE | FALSE | TRUE |
| *HBEGF* | TRUE | FALSE | FALSE |
| *POLE2* | TRUE | TRUE | TRUE |
| *LRBA* | TRUE | FALSE | TRUE |
| *PLXNA1* | TRUE | FALSE | FALSE |
| *ASNS* | TRUE | FALSE | FALSE |
| *ATP5G1* | TRUE | FALSE | FALSE |
| *ABCC4* | TRUE | TRUE | TRUE |
| *ZNF79* | TRUE | FALSE | TRUE |
| *GOLGA3* | TRUE | TRUE | TRUE |
| *UBE2L6* | TRUE | FALSE | TRUE |
| *ICT1* | TRUE | TRUE | TRUE |
| *ZNF282* | TRUE | TRUE | TRUE |
| *PLAT* | TRUE | TRUE | FALSE |
| *RAD23A* | TRUE | FALSE | TRUE |
| *NOM1* | TRUE | FALSE | TRUE |
| *IKZF4* | TRUE | FALSE | TRUE |
| *IL4I1* | TRUE | TRUE | TRUE |
| *SPATS2* | TRUE | FALSE | TRUE |
| *PSPH* | TRUE | FALSE | TRUE |
| *WIZ* | TRUE | TRUE | TRUE |
| *TIMM21* | TRUE | FALSE | TRUE |
| *UTP6* | TRUE | FALSE | TRUE |
| *PRPS2* | TRUE | TRUE | TRUE |
| *TTLL12* | TRUE | TRUE | TRUE |
| *LDHB* | TRUE | TRUE | FALSE |
| *NOP9* | FALSE | TRUE | TRUE |
| *PLRG1* | FALSE | TRUE | TRUE |
| *ZC3H13* | FALSE | TRUE | TRUE |
| *C14orf119* | FALSE | TRUE | TRUE |
| *CCDC117* | FALSE | TRUE | TRUE |
| *ATP1A1* | FALSE | TRUE | TRUE |
| *EIF2B5* | FALSE | TRUE | TRUE |
| *MLF2* | FALSE | TRUE | TRUE |
| *CLIC4* | FALSE | TRUE | TRUE |
| *TMEM147* | FALSE | TRUE | FALSE |
| *API5* | FALSE | TRUE | TRUE |
| *ZNF2* | FALSE | TRUE | FALSE |
| *RAC1* | FALSE | TRUE | TRUE |
| *MRPS18C* | FALSE | TRUE | TRUE |
| *NSUN3* | FALSE | TRUE | TRUE |

| | | | |
|---|---|---|---|
| *TUFM* | FALSE | TRUE | FALSE |
| *CALM2* | FALSE | TRUE | TRUE |
| *DNAJA2* | FALSE | TRUE | TRUE |
| *MRPL20* | FALSE | TRUE | TRUE |
| *LOC100506844* | FALSE | TRUE | FALSE |
| *SRSF1* | FALSE | TRUE | FALSE |
| *SRSF9* | FALSE | TRUE | TRUE |
| *TRAPPC6B* | FALSE | TRUE | TRUE |
| *ZNF584* | FALSE | TRUE | TRUE |
| *ASUN* | FALSE | TRUE | TRUE |
| *MYO1B* | FALSE | TRUE | TRUE |
| *AKIRIN1* | FALSE | TRUE | TRUE |
| *TDG* | FALSE | TRUE | TRUE |
| *KIAA1524* | FALSE | TRUE | TRUE |
| *MID1IP1* | FALSE | TRUE | TRUE |
| *PIGF* | FALSE | TRUE | TRUE |
| *PIGN* | FALSE | TRUE | FALSE |
| *PIGP* | FALSE | TRUE | FALSE |
| *WRAP53* | FALSE | TRUE | TRUE |
| *PIGW* | FALSE | TRUE | TRUE |
| *PIGX* | FALSE | TRUE | TRUE |
| *EBP* | FALSE | TRUE | TRUE |
| *TM2D3* | FALSE | TRUE | TRUE |
| *NTRK2* | FALSE | TRUE | TRUE |
| *NKRF* | FALSE | TRUE | FALSE |
| *TERF2* | FALSE | TRUE | TRUE |
| *DNAJC18* | FALSE | TRUE | TRUE |
| *TTC5* | FALSE | TRUE | TRUE |
| *TTC1* | FALSE | TRUE | TRUE |
| *TTC8* | FALSE | TRUE | TRUE |
| *TPRKB* | FALSE | TRUE | TRUE |
| *PRDM10* | FALSE | TRUE | TRUE |
| *KDM1A* | FALSE | TRUE | TRUE |
| *QRSL1* | FALSE | TRUE | TRUE |
| *BTG3* | FALSE | TRUE | TRUE |
| *C15orf41* | FALSE | TRUE | TRUE |
| *FHOD1* | FALSE | TRUE | TRUE |
| *PSMB1* | FALSE | TRUE | TRUE |
| *HDHD1* | FALSE | TRUE | TRUE |
| *RMI1* | FALSE | TRUE | TRUE |
| *GNA15* | FALSE | TRUE | TRUE |
| *TUBE1* | FALSE | TRUE | TRUE |
| *SMC1A* | FALSE | TRUE | TRUE |
| *KRR1* | FALSE | TRUE | TRUE |

| | | | |
|---|---|---|---|
| *CETN3* | FALSE | TRUE | TRUE |
| *PHRF1* | FALSE | TRUE | TRUE |
| *BRF2* | FALSE | TRUE | FALSE |
| *DNAJC3* | FALSE | TRUE | TRUE |
| *MAP3K4* | FALSE | TRUE | TRUE |
| *PET112* | FALSE | TRUE | TRUE |
| *RAB33A* | FALSE | TRUE | TRUE |
| *CMTR2* | FALSE | TRUE | TRUE |
| *FAM216A* | FALSE | TRUE | TRUE |
| *SEC61G* | FALSE | TRUE | TRUE |
| *MTERFD1* | FALSE | TRUE | TRUE |
| *TFG* | FALSE | TRUE | TRUE |
| *PPP3CB* | FALSE | TRUE | TRUE |
| *LRRC42* | FALSE | TRUE | TRUE |
| *B3GNT2* | FALSE | TRUE | TRUE |
| *TSFM* | FALSE | TRUE | TRUE |
| *GINM1* | FALSE | TRUE | TRUE |
| *TBCCD1* | FALSE | TRUE | TRUE |
| *TIAL1* | FALSE | TRUE | TRUE |
| *TASP1* | FALSE | TRUE | FALSE |
| *SAP30* | FALSE | TRUE | TRUE |
| *CNTROB* | FALSE | TRUE | FALSE |
| *MRPS30* | FALSE | TRUE | TRUE |
| *BRIP1* | FALSE | TRUE | FALSE |
| *CARS* | FALSE | TRUE | TRUE |
| *ACOX1* | FALSE | TRUE | TRUE |
| *ITGB3BP* | FALSE | TRUE | TRUE |
| *FLVCR2* | FALSE | TRUE | TRUE |
| *ATPAF1* | FALSE | TRUE | TRUE |
| *TMEM102* | FALSE | TRUE | TRUE |
| *GHITM* | FALSE | TRUE | TRUE |
| *TIMM8B* | FALSE | TRUE | TRUE |
| *HLTF* | FALSE | TRUE | TRUE |
| *GPALPP1* | FALSE | TRUE | TRUE |
| *VPS41* | FALSE | TRUE | FALSE |
| *EPB41L2* | FALSE | TRUE | FALSE |
| *VPS4A* | FALSE | TRUE | TRUE |
| *DCAF13* | FALSE | TRUE | TRUE |
| *DCAF12* | FALSE | TRUE | TRUE |
| *PALB2* | FALSE | TRUE | TRUE |
| *KLF16* | FALSE | TRUE | TRUE |
| *GBE1* | FALSE | TRUE | FALSE |
| *COQ7* | FALSE | TRUE | TRUE |
| *PPT1* | FALSE | TRUE | FALSE |

| | | | |
|---|---|---|---|
| *H2AFZ* | FALSE | TRUE | TRUE |
| *RNF6* | FALSE | TRUE | TRUE |
| *ADRBK2* | FALSE | TRUE | TRUE |
| *HARS* | FALSE | TRUE | TRUE |
| *FAM118B* | FALSE | TRUE | TRUE |
| *XPNPEP3* | FALSE | TRUE | TRUE |
| *WBSCR22* | FALSE | TRUE | FALSE |
| *ICOS* | FALSE | TRUE | TRUE |
| *SNRPC* | FALSE | TRUE | TRUE |
| *CSNK1A1P1* | FALSE | TRUE | TRUE |
| *ZDHHC12* | FALSE | TRUE | TRUE |
| *GPATCH1* | FALSE | TRUE | TRUE |
| *UBE2D2* | FALSE | TRUE | TRUE |
| *FAM104B* | FALSE | TRUE | TRUE |
| *CCDC51* | FALSE | TRUE | TRUE |
| *PAXIP1* | FALSE | TRUE | TRUE |
| *NUFIP1* | FALSE | TRUE | TRUE |
| *SLC35B2* | FALSE | TRUE | TRUE |
| *FICD* | FALSE | TRUE | TRUE |
| *LONRF1* | FALSE | TRUE | TRUE |
| *WDR3* | FALSE | TRUE | TRUE |
| *VPS25* | FALSE | TRUE | TRUE |
| *FAM60A* | FALSE | TRUE | TRUE |
| *CBWD2* | FALSE | TRUE | TRUE |
| *CBWD1* | FALSE | TRUE | TRUE |
| *MRPL40* | FALSE | TRUE | TRUE |
| *MRPL44* | FALSE | TRUE | TRUE |
| *TRIP6* | FALSE | TRUE | TRUE |
| *MRPL49* | FALSE | TRUE | TRUE |
| *MRPL48* | FALSE | TRUE | TRUE |
| *RUVBL1* | FALSE | TRUE | TRUE |
| *ZZZ3* | FALSE | TRUE | TRUE |
| *PHEX* | FALSE | TRUE | TRUE |
| *TBP* | FALSE | TRUE | TRUE |
| *ADSS* | FALSE | TRUE | TRUE |
| *TIMMDC1* | FALSE | TRUE | TRUE |
| *NUDT15* | FALSE | TRUE | TRUE |
| *RSRC1* | FALSE | TRUE | TRUE |
| *RTCB* | FALSE | TRUE | TRUE |
| *RTCA* | FALSE | TRUE | TRUE |
| *GTF3C3* | FALSE | TRUE | TRUE |
| *BRCA1* | FALSE | TRUE | TRUE |
| *BRCA2* | FALSE | TRUE | TRUE |
| *DDI2* | FALSE | TRUE | TRUE |

| | | | |
|---|---|---|---|
| *PITPNA* | FALSE | TRUE | TRUE |
| *CAAP1* | FALSE | TRUE | TRUE |
| *MRPS23* | FALSE | TRUE | FALSE |
| *PPP2R5E* | FALSE | TRUE | TRUE |
| *JMJD4* | FALSE | TRUE | TRUE |
| *GAS8* | FALSE | TRUE | TRUE |
| *GOT1* | FALSE | TRUE | TRUE |
| *HIBCH* | FALSE | TRUE | FALSE |
| *THUMPD3* | FALSE | TRUE | TRUE |
| *MLLT4* | FALSE | TRUE | TRUE |
| *COPG1* | FALSE | TRUE | TRUE |
| *ZNHIT6* | FALSE | TRUE | TRUE |
| *LYRM2* | FALSE | TRUE | FALSE |
| *LYRM7* | FALSE | TRUE | TRUE |
| *NFS1* | FALSE | TRUE | TRUE |
| *MANEA* | FALSE | TRUE | TRUE |
| *C1orf131* | FALSE | TRUE | TRUE |
| *EIF1B* | FALSE | TRUE | TRUE |
| *NRD1* | FALSE | TRUE | FALSE |
| *MPZL3* | FALSE | TRUE | TRUE |
| *DDOST* | FALSE | TRUE | FALSE |
| *PUSL1* | FALSE | TRUE | TRUE |
| *PTPRE* | FALSE | TRUE | TRUE |
| *PTPRK* | FALSE | TRUE | FALSE |
| *N6AMT1* | FALSE | TRUE | TRUE |
| *HMGN5* | FALSE | TRUE | FALSE |
| *EFTUD1* | FALSE | TRUE | TRUE |
| *ERLIN2* | FALSE | TRUE | TRUE |
| *SPDL1* | FALSE | TRUE | TRUE |
| *SPG20* | FALSE | TRUE | TRUE |
| *WDR35* | FALSE | TRUE | TRUE |
| *FAM206A* | FALSE | TRUE | TRUE |
| *TM9SF1* | FALSE | TRUE | TRUE |
| *HENMT1* | FALSE | TRUE | TRUE |
| *MEPCE* | FALSE | TRUE | TRUE |
| *CPSF6* | FALSE | TRUE | TRUE |
| *CPSF2* | FALSE | TRUE | TRUE |
| *ACAA2* | FALSE | TRUE | TRUE |
| *SIPA1L2* | FALSE | TRUE | TRUE |
| *SLC35F5* | FALSE | TRUE | TRUE |
| *TRMT112* | FALSE | TRUE | FALSE |
| *SLC25A14* | FALSE | TRUE | TRUE |
| *SLC25A11* | FALSE | TRUE | TRUE |
| *ALG2* | FALSE | TRUE | TRUE |

| | | | |
|---|---|---|---|
| *ALG5* | FALSE | TRUE | TRUE |
| *SMARCE1* | FALSE | TRUE | TRUE |
| *RNGTT* | FALSE | TRUE | TRUE |
| *SHPK* | FALSE | TRUE | TRUE |
| *SNHG12* | FALSE | TRUE | FALSE |
| *GIN1* | FALSE | TRUE | TRUE |
| *ZGRF1* | FALSE | TRUE | FALSE |
| *NOTCH1* | FALSE | TRUE | TRUE |
| *KPNA6* | FALSE | TRUE | TRUE |
| *SLC25A19* | FALSE | TRUE | TRUE |
| *C6orf211* | FALSE | TRUE | TRUE |
| *CD68* | FALSE | TRUE | FALSE |
| *MAD2L1BP* | FALSE | TRUE | FALSE |
| *NGDN* | FALSE | TRUE | TRUE |
| *IREB2* | FALSE | TRUE | TRUE |
| *C15orf39* | FALSE | TRUE | TRUE |
| *SLC25A30* | FALSE | TRUE | TRUE |
| *TUBA1B* | FALSE | TRUE | TRUE |
| *DBF4* | FALSE | TRUE | TRUE |
| *TAF2* | FALSE | TRUE | TRUE |
| *TSPAN31* | FALSE | TRUE | TRUE |
| *AGPS* | FALSE | TRUE | TRUE |
| *LRRCC1* | FALSE | TRUE | TRUE |
| *PDCD2* | FALSE | TRUE | TRUE |
| *HDLBP* | FALSE | TRUE | TRUE |
| *SREBF2* | FALSE | TRUE | TRUE |
| *RAB39B* | FALSE | TRUE | TRUE |
| *TATDN3* | FALSE | TRUE | TRUE |
| *GLE1* | FALSE | TRUE | TRUE |
| *MTA2* | FALSE | TRUE | TRUE |
| *BBS5* | FALSE | TRUE | TRUE |
| *C2orf44* | FALSE | TRUE | TRUE |
| *C2orf42* | FALSE | TRUE | TRUE |
| *ZSWIM1* | FALSE | TRUE | TRUE |
| *PIK3C3* | FALSE | TRUE | TRUE |
| *EDC3* | FALSE | TRUE | TRUE |
| *GMDS* | FALSE | TRUE | FALSE |
| *MPV17* | FALSE | TRUE | FALSE |
| *PSMD13* | FALSE | TRUE | TRUE |
| *ETNK1* | FALSE | TRUE | TRUE |
| *EIF4E2* | FALSE | TRUE | TRUE |
| *CALU* | FALSE | TRUE | TRUE |
| *WDR70* | FALSE | TRUE | FALSE |
| *WDR75* | FALSE | TRUE | TRUE |

| | | | |
|---|---|---|---|
| *PPP2R3C* | FALSE | TRUE | TRUE |
| *ZFP64* | FALSE | TRUE | TRUE |
| *ARL14EP* | FALSE | TRUE | TRUE |
| *KIF15* | FALSE | TRUE | FALSE |
| *PAK1* | FALSE | TRUE | TRUE |
| *NSDHL* | FALSE | TRUE | TRUE |
| *TRAF3IP1* | FALSE | TRUE | TRUE |
| *CEP78* | FALSE | TRUE | TRUE |
| *TMEM216* | FALSE | TRUE | TRUE |
| *COX10* | FALSE | TRUE | TRUE |
| *ILKAP* | FALSE | TRUE | TRUE |
| *COX15* | FALSE | TRUE | TRUE |
| *RAB19* | FALSE | TRUE | TRUE |
| *BET1* | FALSE | TRUE | TRUE |
| *NFYB* | FALSE | TRUE | TRUE |
| *RABL3* | FALSE | TRUE | TRUE |
| *CCT6A* | FALSE | TRUE | TRUE |
| *PAF1* | FALSE | TRUE | TRUE |
| *TANK* | FALSE | TRUE | TRUE |
| *TM2D2* | FALSE | TRUE | TRUE |
| *CUL1* | FALSE | TRUE | TRUE |
| *TRIM28* | FALSE | TRUE | TRUE |
| *CREB3* | FALSE | TRUE | TRUE |
| *DYNC2LI1* | FALSE | TRUE | FALSE |
| *NKAP* | FALSE | TRUE | TRUE |
| *ICOSLG* | FALSE | TRUE | TRUE |
| *CEP128* | FALSE | TRUE | FALSE |
| *MRPL2* | FALSE | TRUE | TRUE |
| *ZNF614* | FALSE | TRUE | TRUE |
| *ZNF616* | FALSE | TRUE | TRUE |
| *BAG2* | FALSE | TRUE | TRUE |
| *PDHA1* | FALSE | TRUE | TRUE |
| *COL4A3BP* | FALSE | TRUE | TRUE |
| *RDH11* | FALSE | TRUE | TRUE |
| *NAB2* | FALSE | TRUE | TRUE |
| *MRPS9* | FALSE | TRUE | TRUE |
| *FAM200A* | FALSE | TRUE | TRUE |
| *POLR2E* | FALSE | TRUE | FALSE |
| *POLR2G* | FALSE | TRUE | TRUE |
| *GTF3A* | FALSE | TRUE | FALSE |
| *ASCC1* | FALSE | TRUE | TRUE |
| *MAPK14* | FALSE | TRUE | TRUE |
| *FZD6* | FALSE | TRUE | TRUE |
| *HUS1* | FALSE | TRUE | TRUE |

| | | | |
|---|---|---|---|
| *USO1* | FALSE | TRUE | TRUE |
| *ITFG1* | FALSE | TRUE | FALSE |
| *C14orf80* | FALSE | TRUE | TRUE |
| *GTF2H3* | FALSE | TRUE | TRUE |
| *TVP23B* | FALSE | TRUE | TRUE |
| *ATF5* | FALSE | TRUE | TRUE |
| *ATF4* | FALSE | TRUE | FALSE |
| *ATF1* | FALSE | TRUE | TRUE |
| *ABHD10* | FALSE | TRUE | TRUE |
| *SUMO2* | FALSE | TRUE | FALSE |
| *BLOC1S6* | FALSE | TRUE | TRUE |
| *PPP2R4* | FALSE | TRUE | TRUE |
| *SLC38A9* | FALSE | TRUE | TRUE |
| *STK3* | FALSE | TRUE | TRUE |
| *UBE4B* | FALSE | TRUE | TRUE |
| *SNRNP27* | FALSE | TRUE | TRUE |
| *SENP5* | FALSE | TRUE | TRUE |
| *SENP1* | FALSE | TRUE | TRUE |
| *ING2* | FALSE | TRUE | FALSE |
| *ZNF823* | FALSE | TRUE | FALSE |
| *ECHDC1* | FALSE | TRUE | TRUE |
| *ANKRD37* | FALSE | TRUE | TRUE |
| *ANKRD32* | FALSE | TRUE | TRUE |
| *GLS* | FALSE | TRUE | TRUE |
| *LAPTM4B* | FALSE | TRUE | TRUE |
| *RIF1* | FALSE | TRUE | TRUE |
| *STARD3NL* | FALSE | TRUE | TRUE |
| *SMARCAD1* | FALSE | TRUE | TRUE |
| *ENDOD1* | FALSE | TRUE | TRUE |
| *IPMK* | FALSE | TRUE | TRUE |
| *UBLCP1* | FALSE | TRUE | TRUE |
| *CD2BP2* | FALSE | TRUE | TRUE |
| *TULP3* | FALSE | TRUE | TRUE |
| *FARP2* | FALSE | TRUE | TRUE |
| *C9orf41* | FALSE | TRUE | TRUE |
| *FANCM* | FALSE | TRUE | TRUE |
| *CDK2* | FALSE | TRUE | TRUE |
| *CDK4* | FALSE | TRUE | TRUE |
| *CDK5* | FALSE | TRUE | FALSE |
| *CDK7* | FALSE | TRUE | TRUE |
| *FAM188A* | FALSE | TRUE | TRUE |
| *HSPD1* | FALSE | TRUE | TRUE |
| *SEC11C* | FALSE | TRUE | FALSE |
| *MUL1* | FALSE | TRUE | TRUE |

| | | | |
|---|---|---|---|
| *PPIL1* | FALSE | TRUE | TRUE |
| *PPP1R7* | FALSE | TRUE | TRUE |
| *EMD* | FALSE | TRUE | FALSE |
| *SGOL2* | FALSE | TRUE | TRUE |
| *PHF23* | FALSE | TRUE | TRUE |
| *UBE2M* | FALSE | TRUE | TRUE |
| *FAM162A* | FALSE | TRUE | TRUE |
| *UBE2B* | FALSE | TRUE | TRUE |
| *UBE2Z* | FALSE | TRUE | TRUE |
| *UBE2W* | FALSE | TRUE | TRUE |
| *MORF4L2* | FALSE | TRUE | TRUE |
| *RABEPK* | FALSE | TRUE | TRUE |
| *UFM1* | FALSE | TRUE | TRUE |
| *TXNL4B* | FALSE | TRUE | TRUE |
| *PDCD7* | FALSE | TRUE | TRUE |
| *TMEM33* | FALSE | TRUE | TRUE |
| *MEA1* | FALSE | TRUE | FALSE |
| *GSTCD* | FALSE | TRUE | TRUE |
| *PARG* | FALSE | TRUE | TRUE |
| *PI4K2B* | FALSE | TRUE | TRUE |
| *PI4K2A* | FALSE | TRUE | TRUE |
| *NHLRC2* | FALSE | TRUE | TRUE |
| *CXorf56* | FALSE | TRUE | TRUE |
| *OTUD6B* | FALSE | TRUE | TRUE |
| *DDX1* | FALSE | TRUE | TRUE |
| *NUP205* | FALSE | TRUE | TRUE |
| *CYB5D1* | FALSE | TRUE | TRUE |
| *KIAA0100* | FALSE | TRUE | FALSE |
| *HAUS8* | FALSE | TRUE | FALSE |
| *HAUS6* | FALSE | TRUE | TRUE |
| *HAUS1* | FALSE | TRUE | TRUE |
| *UBXN8* | FALSE | TRUE | TRUE |
| *TNFAIP8* | FALSE | TRUE | TRUE |
| *NIFK* | FALSE | TRUE | TRUE |
| *NXT2* | FALSE | TRUE | TRUE |
| *SSBP1* | FALSE | TRUE | TRUE |
| *BCL10* | FALSE | TRUE | TRUE |
| *TYW1* | FALSE | TRUE | TRUE |
| *RNASEH1-AS1* | FALSE | TRUE | TRUE |
| *RHBDD1* | FALSE | TRUE | TRUE |
| *ZNF770* | FALSE | TRUE | TRUE |
| *ZNF511* | FALSE | TRUE | TRUE |
| *SLC39A9* | FALSE | TRUE | TRUE |
| *SLC39A6* | FALSE | TRUE | TRUE |

| | | | |
|---|---|---|---|
| *SMG8* | FALSE | TRUE | TRUE |
| *SCAF4* | FALSE | TRUE | TRUE |
| *NAA30* | FALSE | TRUE | TRUE |
| *RBM7* | FALSE | TRUE | TRUE |
| *COA6* | FALSE | TRUE | TRUE |
| *COA1* | FALSE | TRUE | TRUE |
| *COA3* | FALSE | TRUE | TRUE |
| *ARMCX3* | FALSE | TRUE | TRUE |
| *RBMX* | FALSE | TRUE | TRUE |
| *FUT8* | FALSE | TRUE | FALSE |
| *R3HCC1L* | FALSE | TRUE | TRUE |
| *RPL23AP7* | FALSE | TRUE | TRUE |
| *PAFAH1B2* | FALSE | TRUE | TRUE |
| *MT1E* | FALSE | TRUE | TRUE |
| *TIFA* | FALSE | TRUE | FALSE |
| *BBS12* | FALSE | TRUE | TRUE |
| *PROSC* | FALSE | TRUE | TRUE |
| *TCERG1* | FALSE | TRUE | TRUE |
| *CNBP* | FALSE | TRUE | TRUE |
| *INTS12* | FALSE | TRUE | TRUE |
| *CSPP1* | FALSE | TRUE | TRUE |
| *OSGEPL1* | FALSE | TRUE | TRUE |
| *YWHAH* | FALSE | TRUE | TRUE |
| *YWHAG* | FALSE | TRUE | TRUE |
| *YWHAE* | FALSE | TRUE | TRUE |
| *SSNA1* | FALSE | TRUE | FALSE |
| *CKAP5* | FALSE | TRUE | TRUE |
| *TRAPPC2L* | FALSE | TRUE | TRUE |
| *TNIP3* | FALSE | TRUE | TRUE |
| *ZAK* | FALSE | TRUE | TRUE |
| *UHRF1BP1* | FALSE | TRUE | TRUE |
| *AAR2* | FALSE | TRUE | TRUE |
| *LIG3* | FALSE | TRUE | TRUE |
| *DBR1* | FALSE | TRUE | TRUE |
| *PRRT3* | FALSE | TRUE | TRUE |
| *FAH* | FALSE | TRUE | TRUE |
| *LLPH* | FALSE | TRUE | TRUE |
| *HNRNPA2B1* | FALSE | TRUE | FALSE |
| *LZIC* | FALSE | TRUE | TRUE |
| *COG7* | FALSE | TRUE | TRUE |
| *COG6* | FALSE | TRUE | TRUE |
| *COG5* | FALSE | TRUE | TRUE |
| *C12orf49* | FALSE | TRUE | TRUE |
| *SCOC* | FALSE | TRUE | TRUE |

| | | | |
|---|---|---|---|
| *POC5* | FALSE | TRUE | TRUE |
| *KBTBD8* | FALSE | TRUE | TRUE |
| *RBM23* | FALSE | TRUE | TRUE |
| *MNAT1* | FALSE | TRUE | TRUE |
| *ARHGAP11B* | FALSE | TRUE | TRUE |
| *DESI2* | FALSE | TRUE | TRUE |
| *TSR1* | FALSE | TRUE | TRUE |
| *TSR3* | FALSE | TRUE | TRUE |
| *TSR2* | FALSE | TRUE | TRUE |
| *DAZAP1* | FALSE | TRUE | FALSE |
| *AHCYL1* | FALSE | TRUE | TRUE |
| *AP4S1* | FALSE | TRUE | TRUE |
| *MFAP1* | FALSE | TRUE | TRUE |
| *C19orf55* | FALSE | TRUE | TRUE |
| *LACTB* | FALSE | TRUE | TRUE |
| *TNFSF11* | FALSE | TRUE | TRUE |
| *GEMIN6* | FALSE | TRUE | TRUE |
| *GEMIN5* | FALSE | TRUE | FALSE |
| *CCT8* | FALSE | TRUE | TRUE |
| *CCT7* | FALSE | TRUE | TRUE |
| *TIMM10* | FALSE | TRUE | TRUE |
| *FBRS* | FALSE | TRUE | TRUE |
| *IFT74* | FALSE | TRUE | TRUE |
| *ZC3H8* | FALSE | TRUE | TRUE |
| *LRR1* | FALSE | TRUE | TRUE |
| *GFM1* | FALSE | TRUE | TRUE |
| *DCTN5* | FALSE | TRUE | TRUE |
| *FAM96B* | FALSE | TRUE | TRUE |
| *RNF25* | FALSE | TRUE | TRUE |
| *UBE2V2* | FALSE | TRUE | TRUE |
| *IGSF3* | FALSE | TRUE | TRUE |
| *NHS* | FALSE | TRUE | TRUE |
| *CNOT3* | FALSE | TRUE | TRUE |
| *CNOT7* | FALSE | TRUE | TRUE |
| *CNOT6* | FALSE | TRUE | TRUE |
| *YARS2* | FALSE | TRUE | TRUE |
| *SLC12A2* | FALSE | TRUE | TRUE |
| *IMP4* | FALSE | TRUE | TRUE |
| *TESK1* | FALSE | TRUE | TRUE |
| *OSBP* | FALSE | TRUE | TRUE |
| *SNX4* | FALSE | TRUE | TRUE |
| *CCDC125* | FALSE | TRUE | TRUE |
| *CEP170* | FALSE | TRUE | TRUE |
| *AHI1* | FALSE | TRUE | TRUE |

| | | | |
|---|---|---|---|
| *FANCD2* | FALSE | TRUE | TRUE |
| *RRP8* | FALSE | TRUE | TRUE |
| *HNRNPL* | FALSE | TRUE | FALSE |
| *ATP6V0B* | FALSE | TRUE | TRUE |
| *SMEK2* | FALSE | TRUE | TRUE |
| *RCE1* | FALSE | TRUE | TRUE |
| *NEU3* | FALSE | TRUE | TRUE |
| *UQCR10* | FALSE | TRUE | TRUE |
| *RPRD1B* | FALSE | TRUE | TRUE |
| *MED17* | FALSE | TRUE | TRUE |
| *MED18* | FALSE | TRUE | FALSE |
| *TOPBP1* | FALSE | TRUE | TRUE |
| *PMAIP1* | FALSE | TRUE | TRUE |
| *SLC30A6* | FALSE | TRUE | TRUE |
| *SLC30A1* | FALSE | TRUE | TRUE |
| *SNRNP200* | FALSE | TRUE | FALSE |
| *NARS2* | FALSE | TRUE | FALSE |
| *NUPL1* | FALSE | TRUE | TRUE |
| *NUPL2* | FALSE | TRUE | TRUE |
| *NCAPG* | FALSE | TRUE | TRUE |
| *EIF5A* | FALSE | TRUE | TRUE |
| *OXCT1* | FALSE | TRUE | TRUE |
| *APTX* | FALSE | TRUE | TRUE |
| *CD83* | FALSE | TRUE | TRUE |
| *HK1* | FALSE | TRUE | TRUE |
| *PIK3R4* | FALSE | TRUE | TRUE |
| *HIATL1* | FALSE | TRUE | TRUE |
| *CHRNA5* | FALSE | TRUE | TRUE |
| *ID2* | FALSE | TRUE | TRUE |
| *FOPNL* | FALSE | TRUE | TRUE |
| *SEC31A* | FALSE | TRUE | TRUE |
| *PEF1* | FALSE | TRUE | TRUE |
| *ACTL6A* | FALSE | TRUE | TRUE |
| *ORC5* | FALSE | TRUE | TRUE |
| *RCC2* | FALSE | TRUE | TRUE |
| *NCBP2* | FALSE | TRUE | TRUE |
| *CBLB* | FALSE | TRUE | FALSE |
| *ZNF41* | FALSE | TRUE | TRUE |
| *TRAPPC4* | FALSE | TRUE | TRUE |
| *PSMG3* | FALSE | TRUE | TRUE |
| *PPID* | FALSE | TRUE | TRUE |
| *FKTN* | FALSE | TRUE | FALSE |
| *ZSWIM3* | FALSE | TRUE | FALSE |
| *NOL8* | FALSE | TRUE | TRUE |

| | | | |
|---|---|---|---|
| *SAR1A* | FALSE | TRUE | TRUE |
| *CLPTM1* | FALSE | TRUE | TRUE |
| *CYB5B* | FALSE | TRUE | TRUE |
| *SYTL3* | FALSE | TRUE | TRUE |
| *VIMP* | FALSE | TRUE | TRUE |
| *MRPL36* | FALSE | TRUE | TRUE |
| *MRPL32* | FALSE | TRUE | TRUE |
| *MRPL33* | FALSE | TRUE | FALSE |
| *THOC1* | FALSE | TRUE | TRUE |
| *NUP85* | FALSE | TRUE | TRUE |
| *PARPBP* | FALSE | TRUE | TRUE |
| *FDFT1* | FALSE | TRUE | TRUE |
| *NUDT9* | FALSE | TRUE | TRUE |
| *NUDT5* | FALSE | TRUE | TRUE |
| *LARS* | FALSE | TRUE | FALSE |
| *FAM168B* | FALSE | TRUE | TRUE |
| *IPPK* | FALSE | TRUE | TRUE |
| *NDFIP1* | FALSE | TRUE | TRUE |
| *KIF11* | FALSE | TRUE | TRUE |
| *GTF3C2* | FALSE | TRUE | TRUE |
| *TCP1* | FALSE | TRUE | TRUE |
| *MAD2L1* | FALSE | TRUE | TRUE |
| *SLC16A3* | FALSE | TRUE | TRUE |
| *DYNLL1* | FALSE | TRUE | TRUE |
| *RBM4B* | FALSE | TRUE | TRUE |
| *RBM45* | FALSE | TRUE | TRUE |
| *ABCF3* | FALSE | TRUE | TRUE |
| *LRCH3* | FALSE | TRUE | TRUE |
| *PSMA7* | FALSE | TRUE | TRUE |
| *FBXO45* | FALSE | TRUE | TRUE |
| *JAGN1* | FALSE | TRUE | TRUE |
| *NIP7* | FALSE | TRUE | TRUE |
| *PEX12* | FALSE | TRUE | TRUE |
| *UBR1* | FALSE | TRUE | TRUE |
| *UBR5* | FALSE | TRUE | TRUE |
| *SRPR* | FALSE | TRUE | TRUE |
| *PPP2CB* | FALSE | TRUE | TRUE |
| *CCNC* | FALSE | TRUE | TRUE |
| *TBRG4* | FALSE | TRUE | TRUE |
| *MRPL16* | FALSE | TRUE | TRUE |
| *AIFM1* | FALSE | TRUE | TRUE |
| *FAM217B* | FALSE | TRUE | TRUE |
| *GPN3* | FALSE | TRUE | TRUE |
| *NFKB2* | FALSE | TRUE | TRUE |

| | | | |
|---|---|---|---|
| *EMILIN2* | FALSE | TRUE | TRUE |
| *PIAS2* | FALSE | TRUE | TRUE |
| *PIAS4* | FALSE | TRUE | TRUE |
| *PHF6* | FALSE | TRUE | TRUE |
| *ALS2* | FALSE | TRUE | TRUE |
| *SDHD* | FALSE | TRUE | TRUE |
| *ZCCHC10* | FALSE | TRUE | TRUE |
| *CCDC142* | FALSE | TRUE | TRUE |
| *FAM114A2* | FALSE | TRUE | TRUE |
| *PTGR2* | FALSE | TRUE | TRUE |
| *TUBB4B* | FALSE | TRUE | TRUE |
| *BMS1* | FALSE | TRUE | TRUE |
| *AHSA1* | FALSE | TRUE | TRUE |
| *OCIAD1* | FALSE | TRUE | FALSE |
| *GLMN* | FALSE | TRUE | FALSE |
| *TTC33* | FALSE | TRUE | TRUE |
| *GEMIN2* | FALSE | TRUE | TRUE |
| *C4orf46* | FALSE | TRUE | TRUE |
| *ERAL1* | FALSE | TRUE | TRUE |
| *DDX18* | FALSE | TRUE | TRUE |
| *TRIP12* | FALSE | TRUE | TRUE |
| *NCLN* | FALSE | TRUE | FALSE |
| *EIF3I* | FALSE | TRUE | FALSE |
| *EIF3D* | FALSE | TRUE | TRUE |
| *HSBP1* | FALSE | TRUE | TRUE |
| *SRRD* | FALSE | TRUE | TRUE |
| *KDM6B* | FALSE | TRUE | FALSE |
| *RNF145* | FALSE | TRUE | TRUE |
| *ITPR1* | FALSE | TRUE | FALSE |
| *MSANTD4* | FALSE | TRUE | TRUE |
| *VPS37C* | FALSE | TRUE | TRUE |
| *SFPQ* | FALSE | TRUE | TRUE |
| *KIAA0753* | FALSE | TRUE | TRUE |
| *PALLD* | FALSE | TRUE | TRUE |
| *MCM7* | FALSE | TRUE | TRUE |
| *MRGBP* | FALSE | TRUE | TRUE |
| *ZNF146* | FALSE | TRUE | TRUE |
| *CPT2* | FALSE | TRUE | TRUE |
| *SLC35A4* | FALSE | TRUE | TRUE |
| *SLC35A5* | FALSE | TRUE | TRUE |
| *SLC35A2* | FALSE | TRUE | TRUE |
| *FAF2* | FALSE | TRUE | TRUE |
| *PGM1* | FALSE | TRUE | TRUE |
| *SLC25A46* | FALSE | TRUE | TRUE |

| | | | |
|---|---|---|---|
| *STRADA* | FALSE | TRUE | FALSE |
| *GCN1L1* | FALSE | TRUE | TRUE |
| *METTL5* | FALSE | TRUE | TRUE |
| *STAT5A* | FALSE | TRUE | TRUE |
| *PXK* | FALSE | TRUE | TRUE |
| *ESF1* | FALSE | TRUE | TRUE |
| *BRAP* | FALSE | TRUE | TRUE |
| *P4HA1* | FALSE | TRUE | TRUE |
| *MRPL51* | FALSE | TRUE | TRUE |
| *ZNF681* | FALSE | TRUE | TRUE |
| *AP5M1* | FALSE | TRUE | TRUE |
| *POP1* | FALSE | TRUE | TRUE |
| *POP5* | FALSE | TRUE | FALSE |
| *POP4* | FALSE | TRUE | TRUE |
| *FKBP2* | FALSE | TRUE | FALSE |
| *NF2* | FALSE | TRUE | TRUE |
| *NF1* | FALSE | TRUE | FALSE |
| *SMC2* | FALSE | TRUE | TRUE |
| *SMC6* | FALSE | TRUE | TRUE |
| *SFR1* | FALSE | TRUE | TRUE |
| *ARL8B* | FALSE | TRUE | TRUE |
| *ANKRD13C* | FALSE | TRUE | TRUE |
| *KPTN* | FALSE | TRUE | FALSE |
| *SLC41A2* | FALSE | TRUE | TRUE |
| *DDX50* | FALSE | TRUE | TRUE |
| *RPL7L1* | FALSE | TRUE | TRUE |
| *C11orf82* | FALSE | TRUE | TRUE |
| *GTF2A1* | FALSE | TRUE | TRUE |
| *COQ5* | FALSE | TRUE | TRUE |
| *CCNB2* | FALSE | TRUE | TRUE |
| *CCNB1* | FALSE | TRUE | TRUE |
| *COQ3* | FALSE | TRUE | TRUE |
| *COQ2* | FALSE | TRUE | TRUE |
| *CCT4* | FALSE | TRUE | TRUE |
| *CLN5* | FALSE | TRUE | TRUE |
| *RNF181* | FALSE | TRUE | TRUE |
| *ZBTB21* | FALSE | TRUE | TRUE |
| *RIC8A* | FALSE | TRUE | TRUE |
| *BCS1L* | FALSE | TRUE | TRUE |
| *UCHL3* | FALSE | TRUE | TRUE |
| *GFM2* | FALSE | TRUE | TRUE |
| *AIMP1* | FALSE | TRUE | TRUE |
| *MDH1* | FALSE | TRUE | TRUE |
| *VPS33A* | FALSE | TRUE | TRUE |

| | | | |
|---|---|---|---|
| *NIF3L1* | FALSE | TRUE | TRUE |
| *TTC9C* | FALSE | TRUE | TRUE |
| *TRMT11* | FALSE | TRUE | TRUE |
| *SNRNP40* | FALSE | TRUE | TRUE |
| *SNAPC3* | FALSE | TRUE | TRUE |
| *METAP1* | FALSE | TRUE | TRUE |
| *LANCL2* | FALSE | TRUE | TRUE |
| *GNPNAT1* | FALSE | TRUE | TRUE |
| *HNRNPH3* | FALSE | TRUE | TRUE |
| *MRPS34* | FALSE | TRUE | TRUE |
| *MRPS36* | FALSE | TRUE | TRUE |
| *TBC1D7* | FALSE | TRUE | TRUE |
| *SEL1L* | FALSE | TRUE | TRUE |
| *PDSS2* | FALSE | TRUE | FALSE |
| *OSGIN2* | FALSE | TRUE | TRUE |
| *TRMT10A* | FALSE | TRUE | TRUE |
| *KCTD20* | FALSE | TRUE | TRUE |
| *KCTD21* | FALSE | TRUE | TRUE |
| *FAM96A* | FALSE | TRUE | TRUE |
| *PRPF38A* | FALSE | TRUE | TRUE |
| *RPP38* | FALSE | TRUE | FALSE |
| *G3BP1* | FALSE | TRUE | TRUE |
| *CNEP1R1* | FALSE | TRUE | TRUE |
| *WRN* | FALSE | TRUE | TRUE |
| *POLR3F* | FALSE | TRUE | TRUE |
| *UROD* | FALSE | TRUE | FALSE |
| *HMMR* | FALSE | TRUE | TRUE |
| *ZDHHC9* | FALSE | TRUE | TRUE |
| *POLQ* | FALSE | TRUE | TRUE |
| *TAF13* | FALSE | TRUE | TRUE |
| *FH* | FALSE | TRUE | TRUE |
| *CDC123* | FALSE | TRUE | TRUE |
| *CYP51A1* | FALSE | TRUE | TRUE |
| *TAF1A* | FALSE | TRUE | TRUE |
| *RYBP* | FALSE | TRUE | TRUE |
| *HSD17B4* | FALSE | TRUE | TRUE |
| *PRMT1* | FALSE | TRUE | TRUE |
| *SPATA5L1* | FALSE | TRUE | TRUE |
| *FAM173B* | FALSE | TRUE | TRUE |
| *PROSER1* | FALSE | TRUE | TRUE |
| *CNOT11* | FALSE | TRUE | TRUE |
| *ZWILCH* | FALSE | TRUE | TRUE |
| *PAAF1* | FALSE | TRUE | TRUE |
| *CBX1* | FALSE | TRUE | FALSE |

| | | | |
|---|---|---|---|
| *FASTKD2* | FALSE | TRUE | TRUE |
| *FASTKD3* | FALSE | TRUE | TRUE |
| *BTLA* | FALSE | TRUE | TRUE |
| *WDR24* | FALSE | TRUE | TRUE |
| *WDR25* | FALSE | TRUE | TRUE |
| *SRP72* | FALSE | TRUE | TRUE |
| *LYST* | FALSE | TRUE | TRUE |
| *RQCD1* | FALSE | TRUE | TRUE |
| *PFKM* | FALSE | TRUE | FALSE |
| *ECSCR* | FALSE | TRUE | FALSE |
| *TAMM41* | FALSE | TRUE | TRUE |
| *ADPGK* | FALSE | TRUE | TRUE |
| *FLVCR1* | FALSE | TRUE | FALSE |
| *MKS1* | FALSE | TRUE | FALSE |
| *RPAP2* | FALSE | TRUE | TRUE |
| *RPAP3* | FALSE | TRUE | TRUE |
| *RPUSD1* | FALSE | TRUE | TRUE |
| *TP53RK* | FALSE | TRUE | TRUE |
| *DPM1* | FALSE | TRUE | TRUE |
| *SUV39H1* | FALSE | TRUE | TRUE |
| *SCO2* | FALSE | TRUE | TRUE |
| *MMADHC* | FALSE | TRUE | TRUE |
| *TDP2* | FALSE | TRUE | TRUE |
| *FAM175B* | FALSE | TRUE | TRUE |
| *C14orf166* | FALSE | TRUE | TRUE |
| *TMED8* | FALSE | TRUE | TRUE |
| *HNRNPK* | FALSE | TRUE | TRUE |
| *HNRNPC* | FALSE | TRUE | FALSE |
| *SLC25A3* | FALSE | TRUE | TRUE |
| *UPF3B* | FALSE | TRUE | TRUE |
| *DOK1* | FALSE | TRUE | TRUE |
| *PLS1* | FALSE | TRUE | TRUE |
| *CLASP2* | FALSE | TRUE | TRUE |
| *CLASP1* | FALSE | TRUE | TRUE |
| *PDIA3* | FALSE | TRUE | TRUE |
| *ARMC5* | FALSE | TRUE | TRUE |
| *ARMC1* | FALSE | TRUE | TRUE |
| *AARS* | FALSE | TRUE | TRUE |
| *SPATA5* | FALSE | TRUE | TRUE |
| *HSPA9* | FALSE | TRUE | TRUE |
| *CLEC2D* | FALSE | TRUE | FALSE |
| *TSG101* | FALSE | TRUE | TRUE |
| *TTK* | FALSE | TRUE | TRUE |
| *GARS* | FALSE | TRUE | TRUE |

| | | | |
|---|---|---|---|
| *ATAD1* | FALSE | TRUE | TRUE |
| *TMEM199* | FALSE | TRUE | TRUE |
| *MIOS* | FALSE | TRUE | TRUE |
| *HOMER1* | FALSE | TRUE | TRUE |
| *THAP6* | FALSE | TRUE | FALSE |
| *AHCTF1* | FALSE | TRUE | TRUE |
| *CHM* | FALSE | TRUE | TRUE |
| *TAF5L* | FALSE | TRUE | TRUE |
| *MED6* | FALSE | TRUE | TRUE |
| *MED8* | FALSE | TRUE | TRUE |
| *KXD1* | FALSE | TRUE | TRUE |
| *SNRPA1* | FALSE | TRUE | FALSE |
| *RPS6KB2* | FALSE | TRUE | FALSE |
| *UTP18* | FALSE | TRUE | TRUE |
| *LACC1* | FALSE | TRUE | TRUE |
| *DCAF7* | FALSE | TRUE | TRUE |
| *POLR3G* | FALSE | TRUE | FALSE |
| *POLR3B* | FALSE | TRUE | TRUE |
| *R3HDM1* | FALSE | TRUE | FALSE |
| *PRMT5* | FALSE | TRUE | TRUE |
| *ORMDL2* | FALSE | TRUE | TRUE |
| *CD200* | FALSE | TRUE | TRUE |
| *IARS2* | FALSE | TRUE | TRUE |
| *CANX* | FALSE | TRUE | FALSE |
| *SCRN3* | FALSE | TRUE | TRUE |
| *TMEM206* | FALSE | TRUE | TRUE |
| *TMEM208* | FALSE | TRUE | TRUE |
| *VPRBP* | FALSE | TRUE | TRUE |
| *SLC35B1* | FALSE | TRUE | FALSE |
| *SMARCB1* | FALSE | TRUE | TRUE |
| *HAVCR2* | FALSE | TRUE | TRUE |
| *MTBP* | FALSE | TRUE | TRUE |
| *MAFK* | FALSE | TRUE | TRUE |
| *WDR55* | FALSE | TRUE | TRUE |
| *ARPC1A* | FALSE | TRUE | TRUE |
| *ACSL4* | FALSE | TRUE | TRUE |
| *INTS6* | FALSE | TRUE | TRUE |
| *ZNF585A* | FALSE | TRUE | TRUE |
| *RCN2* | FALSE | TRUE | TRUE |
| *ACO1* | FALSE | TRUE | TRUE |
| *PBK* | FALSE | TRUE | FALSE |
| *ZNF620* | FALSE | TRUE | TRUE |
| *ZNF622* | FALSE | TRUE | TRUE |
| *PRPF19* | FALSE | TRUE | TRUE |

| | | | |
|---|---|---|---|
| *POLR1A* | FALSE | TRUE | TRUE |
| *POLR1C* | FALSE | TRUE | TRUE |
| *C5orf15* | FALSE | TRUE | TRUE |
| *ASH2L* | FALSE | TRUE | TRUE |
| *KIF21A* | FALSE | TRUE | TRUE |
| *IL21R* | FALSE | TRUE | TRUE |
| *NARG2* | FALSE | TRUE | TRUE |
| *TMEM222* | FALSE | TRUE | TRUE |
| *HEATR6* | FALSE | TRUE | FALSE |
| *HEATR1* | FALSE | TRUE | TRUE |
| *NUP35* | FALSE | TRUE | TRUE |
| *NUP37* | FALSE | TRUE | FALSE |
| *MIPEP* | FALSE | TRUE | TRUE |
| *CACUL1* | FALSE | TRUE | TRUE |
| *NABP2* | FALSE | TRUE | TRUE |
| *NOMO2* | FALSE | TRUE | FALSE |
| *BAZ1A* | FALSE | TRUE | TRUE |
| *MAP2K4P1* | FALSE | TRUE | TRUE |
| *CCAR1* | FALSE | TRUE | TRUE |
| *CCAR2* | FALSE | TRUE | TRUE |
| *VKORC1* | FALSE | TRUE | TRUE |
| *OXSM* | FALSE | TRUE | TRUE |
| *DNAJC15* | FALSE | TRUE | TRUE |
| *LMAN2* | FALSE | TRUE | FALSE |
| *PFKFB2* | FALSE | TRUE | TRUE |
| *HNRNPD* | FALSE | TRUE | TRUE |
| *PPP4R2* | FALSE | TRUE | TRUE |
| *NUP214* | FALSE | TRUE | TRUE |
| *IDH1* | FALSE | TRUE | TRUE |
| *SETD3* | FALSE | TRUE | TRUE |
| *SETD7* | FALSE | TRUE | TRUE |
| *STX1A* | FALSE | TRUE | TRUE |
| *FARS2* | FALSE | TRUE | FALSE |
| *BTBD10* | FALSE | TRUE | TRUE |
| *MASTL* | FALSE | TRUE | TRUE |
| *TSEN2* | FALSE | TRUE | TRUE |
| *C19orf82* | FALSE | TRUE | TRUE |
| *MAST2* | FALSE | TRUE | FALSE |
| *C5orf34* | FALSE | TRUE | TRUE |
| *STX12* | FALSE | TRUE | TRUE |
| *APOPT1* | FALSE | TRUE | TRUE |
| *TMEM167A* | FALSE | TRUE | TRUE |
| *TRMT61B* | FALSE | TRUE | TRUE |
| *MUM1* | FALSE | TRUE | TRUE |

| | | | |
|---|---|---|---|
| *BRAT1* | FALSE | TRUE | TRUE |
| *STMN1* | FALSE | TRUE | FALSE |
| *FAM161A* | FALSE | TRUE | TRUE |
| *GTF2H1* | FALSE | TRUE | TRUE |
| *MTFR1* | FALSE | TRUE | TRUE |
| *MTFR2* | FALSE | TRUE | TRUE |
| *NAA20* | FALSE | TRUE | TRUE |
| *SGCB* | FALSE | TRUE | TRUE |
| *DUSP2* | FALSE | TRUE | FALSE |
| *IKBKAP* | FALSE | TRUE | TRUE |
| *NPM3* | FALSE | TRUE | FALSE |
| *SCFD1* | FALSE | TRUE | TRUE |
| *PPP3R1* | FALSE | TRUE | TRUE |
| *FAM222B* | FALSE | TRUE | TRUE |
| *SERP1* | FALSE | TRUE | TRUE |
| *PUS7L* | FALSE | TRUE | TRUE |
| *BNIP1* | FALSE | TRUE | TRUE |
| *SDF2* | FALSE | TRUE | TRUE |
| *EMC2* | FALSE | TRUE | TRUE |
| *SNX12* | FALSE | TRUE | TRUE |
| *C17orf80* | FALSE | TRUE | TRUE |
| *TNKS1BP1* | FALSE | TRUE | TRUE |
| *SPCS1* | FALSE | TRUE | FALSE |
| *DHFRL1* | FALSE | TRUE | FALSE |
| *SLC38A1* | FALSE | TRUE | FALSE |
| *WDR89* | FALSE | TRUE | TRUE |
| *CENPJ* | FALSE | TRUE | TRUE |
| *SIMC1* | FALSE | TRUE | TRUE |
| *WDHD1* | FALSE | TRUE | TRUE |
| *ZNF764* | FALSE | TRUE | TRUE |
| *FAM185A* | FALSE | TRUE | TRUE |
| *PUS3* | FALSE | TRUE | FALSE |
| *PHF14* | FALSE | TRUE | TRUE |
| *POLA1* | FALSE | TRUE | FALSE |
| *RB1* | FALSE | TRUE | TRUE |
| *CUL4A* | FALSE | TRUE | TRUE |
| *C8orf33* | FALSE | TRUE | TRUE |
| *NDUFB6* | FALSE | TRUE | TRUE |
| *NDUFB5* | FALSE | TRUE | TRUE |
| *TMOD3* | FALSE | TRUE | TRUE |
| *ANKRD40* | FALSE | TRUE | FALSE |
| *ANKRD42* | FALSE | TRUE | TRUE |
| *MED20* | FALSE | TRUE | TRUE |
| *RBM34* | FALSE | TRUE | TRUE |

| | | | |
|---|---|---|---|
| *YARS* | FALSE | TRUE | TRUE |
| *BCCIP* | FALSE | TRUE | TRUE |
| *NVL* | FALSE | TRUE | TRUE |
| *ZNF35* | FALSE | TRUE | TRUE |
| *ZNF30* | FALSE | TRUE | TRUE |
| *C21orf59* | FALSE | TRUE | TRUE |
| *PUS10* | FALSE | TRUE | TRUE |
| *UGGT1* | FALSE | TRUE | TRUE |
| *RAD51C* | FALSE | TRUE | TRUE |
| *DIP2B* | FALSE | TRUE | FALSE |
| *DCLRE1B* | FALSE | TRUE | FALSE |
| *ECT2* | FALSE | TRUE | TRUE |
| *ZCCHC4* | FALSE | TRUE | TRUE |
| *MRPS31* | FALSE | TRUE | TRUE |
| *ADPRHL2* | FALSE | TRUE | TRUE |
| *DARS* | FALSE | TRUE | TRUE |
| *LYPLA2* | FALSE | TRUE | TRUE |
| *RPE* | FALSE | TRUE | TRUE |
| *SPAG5* | FALSE | TRUE | TRUE |
| *SPAG1* | FALSE | TRUE | TRUE |
| *KLHL7* | FALSE | TRUE | FALSE |
| *KLHL2* | FALSE | TRUE | TRUE |
| *KDELR1* | FALSE | TRUE | FALSE |
| *ST13* | FALSE | TRUE | TRUE |
| *LOC728554* | FALSE | TRUE | TRUE |
| *MPV17L2* | FALSE | TRUE | TRUE |
| *YAE1D1* | FALSE | TRUE | TRUE |
| *AVL9* | FALSE | TRUE | TRUE |
| *DPY19L3* | FALSE | TRUE | FALSE |
| *TMEM39B* | FALSE | TRUE | TRUE |
| *RNLS* | FALSE | TRUE | FALSE |
| *FKBP4* | FALSE | TRUE | TRUE |
| *SEC23IP* | FALSE | TRUE | TRUE |
| *MANF* | FALSE | TRUE | TRUE |
| *SAMD1* | FALSE | TRUE | TRUE |
| *GM2A* | FALSE | TRUE | TRUE |
| *HSD17B10* | FALSE | TRUE | FALSE |
| *PANK3* | FALSE | TRUE | TRUE |
| *HEATR5A* | FALSE | TRUE | TRUE |
| *SARS* | FALSE | TRUE | TRUE |
| *BEND3* | FALSE | TRUE | TRUE |
| *CXorf21* | FALSE | TRUE | FALSE |
| *SUCLA2* | FALSE | TRUE | TRUE |
| *RFT1* | FALSE | TRUE | TRUE |

| | | | |
|---|---|---|---|
| *HARBI1* | FALSE | TRUE | TRUE |
| *MT2A* | FALSE | TRUE | TRUE |
| *PSMD5* | FALSE | TRUE | FALSE |
| *PSMD7* | FALSE | TRUE | TRUE |
| *PSMD6* | FALSE | TRUE | TRUE |
| *RPGRIP1L* | FALSE | TRUE | TRUE |
| *AASDHPPT* | FALSE | TRUE | TRUE |
| *TGDS* | FALSE | TRUE | TRUE |
| *UPRT* | FALSE | TRUE | TRUE |
| *FAM213B* | FALSE | TRUE | TRUE |
| *RBBP8* | FALSE | TRUE | TRUE |
| *HPSE* | FALSE | TRUE | TRUE |
| *IAH1* | FALSE | TRUE | TRUE |
| *YIPF4* | FALSE | TRUE | TRUE |
| *FTSJ2* | FALSE | TRUE | TRUE |
| *FTSJ3* | FALSE | TRUE | FALSE |
| *SHFM1* | FALSE | TRUE | FALSE |
| *FXR2* | FALSE | TRUE | TRUE |
| *CST7* | FALSE | TRUE | FALSE |
| *AMIGO2* | FALSE | TRUE | TRUE |
| *RNF34* | FALSE | TRUE | TRUE |
| *INTS2* | FALSE | TRUE | FALSE |
| *INTS5* | FALSE | TRUE | TRUE |
| *INTS4* | FALSE | TRUE | TRUE |
| *MPP5* | FALSE | TRUE | TRUE |
| *ETFDH* | FALSE | TRUE | TRUE |
| *GBP2* | FALSE | TRUE | TRUE |
| *CCDC134* | FALSE | TRUE | TRUE |
| *RBFOX2* | FALSE | TRUE | TRUE |
| *CHRNB1* | FALSE | TRUE | TRUE |
| *MAP2K3* | FALSE | TRUE | TRUE |
| *MAP2K4* | FALSE | TRUE | TRUE |
| *PHF8* | FALSE | TRUE | TRUE |
| *SMARCAL1* | FALSE | TRUE | TRUE |
| *PRKAR1A* | FALSE | TRUE | TRUE |
| *GOLGA2* | FALSE | TRUE | TRUE |
| *PHF20L1* | FALSE | TRUE | TRUE |
| *CAND1* | FALSE | TRUE | TRUE |
| *PRPS1* | FALSE | TRUE | TRUE |
| *C1orf198* | FALSE | TRUE | TRUE |
| *SELT* | FALSE | TRUE | TRUE |
| *AARS2* | FALSE | FALSE | TRUE |
| *ABCB7* | FALSE | FALSE | TRUE |
| *ABCD3* | FALSE | FALSE | TRUE |

| | | | |
|---|---|---|---|
| *ABCE1* | FALSE | FALSE | TRUE |
| *ABHD11* | FALSE | FALSE | TRUE |
| *ABHD5* | FALSE | FALSE | TRUE |
| *ABI3* | FALSE | FALSE | TRUE |
| *ACADM* | FALSE | FALSE | TRUE |
| *ACAT2* | FALSE | FALSE | TRUE |
| *ACN9* | FALSE | FALSE | TRUE |
| *ACO2* | FALSE | FALSE | TRUE |
| *ACOT13* | FALSE | FALSE | TRUE |
| *ACOT7* | FALSE | FALSE | TRUE |
| *ACOT9* | FALSE | FALSE | TRUE |
| *ACP1* | FALSE | FALSE | TRUE |
| *ACSL1* | FALSE | FALSE | TRUE |
| *ACSL3* | FALSE | FALSE | TRUE |
| *ACTR10* | FALSE | FALSE | TRUE |
| *ACTR1A* | FALSE | FALSE | TRUE |
| *ACTR3* | FALSE | FALSE | TRUE |
| *ACTR3B* | FALSE | FALSE | TRUE |
| *ACTR8* | FALSE | FALSE | TRUE |
| *ADAM9* | FALSE | FALSE | TRUE |
| *ADAP1* | FALSE | FALSE | TRUE |
| *ADAT2* | FALSE | FALSE | TRUE |
| *ADH5* | FALSE | FALSE | TRUE |
| *ADIPOR2* | FALSE | FALSE | TRUE |
| *ADSL* | FALSE | FALSE | TRUE |
| *AFG3L2* | FALSE | FALSE | TRUE |
| *AGGF1* | FALSE | FALSE | TRUE |
| *AGK* | FALSE | FALSE | TRUE |
| *AGO2* | FALSE | FALSE | TRUE |
| *AGPAT3* | FALSE | FALSE | TRUE |
| *AGPAT5* | FALSE | FALSE | TRUE |
| *AHR* | FALSE | FALSE | TRUE |
| *AK6* | FALSE | FALSE | TRUE |
| *AKAP1* | FALSE | FALSE | TRUE |
| *AKIP1* | FALSE | FALSE | TRUE |
| *ALCAM* | FALSE | FALSE | TRUE |
| *ALDH4A1* | FALSE | FALSE | TRUE |
| *ALG13* | FALSE | FALSE | TRUE |
| *ALG14* | FALSE | FALSE | TRUE |
| *ALKBH1* | FALSE | FALSE | TRUE |
| *ALYREF* | FALSE | FALSE | TRUE |
| *AMD1* | FALSE | FALSE | TRUE |
| *AMMECR1L* | FALSE | FALSE | TRUE |
| *AMPD2* | FALSE | FALSE | TRUE |

| | | | |
|---|---|---|---|
| *ANAPC10* | FALSE | FALSE | TRUE |
| *ANKRD17* | FALSE | FALSE | TRUE |
| *ANKRD33B* | FALSE | FALSE | TRUE |
| *ANKRD46* | FALSE | FALSE | TRUE |
| *ANKRD54* | FALSE | FALSE | TRUE |
| *ANKS1A* | FALSE | FALSE | TRUE |
| *ANP32A* | FALSE | FALSE | TRUE |
| *AP1AR* | FALSE | FALSE | TRUE |
| *AP3B1* | FALSE | FALSE | TRUE |
| *AP3M1* | FALSE | FALSE | TRUE |
| *AP4B1* | FALSE | FALSE | TRUE |
| *APEX1* | FALSE | FALSE | TRUE |
| *APEX2* | FALSE | FALSE | TRUE |
| *APIP* | FALSE | FALSE | TRUE |
| *APOL2* | FALSE | FALSE | TRUE |
| *APOO* | FALSE | FALSE | TRUE |
| *APOOL* | FALSE | FALSE | TRUE |
| *AQR* | FALSE | FALSE | TRUE |
| *ARCN1* | FALSE | FALSE | TRUE |
| *ARF1* | FALSE | FALSE | TRUE |
| *ARF4* | FALSE | FALSE | TRUE |
| *ARFIP1* | FALSE | FALSE | TRUE |
| *ARG2* | FALSE | FALSE | TRUE |
| *ARHGAP31* | FALSE | FALSE | TRUE |
| *ARHGEF12* | FALSE | FALSE | TRUE |
| *ARID5A* | FALSE | FALSE | TRUE |
| *ARL13B* | FALSE | FALSE | TRUE |
| *ARL3* | FALSE | FALSE | TRUE |
| *ARL5B* | FALSE | FALSE | TRUE |
| *ARMC10* | FALSE | FALSE | TRUE |
| *ARMC8* | FALSE | FALSE | TRUE |
| *ARMCX6* | FALSE | FALSE | TRUE |
| *ARNTL2* | FALSE | FALSE | TRUE |
| *ARV1* | FALSE | FALSE | TRUE |
| *ASAH2B* | FALSE | FALSE | TRUE |
| *ASNSD1* | FALSE | FALSE | TRUE |
| *ASPHD2* | FALSE | FALSE | TRUE |
| *ATE1* | FALSE | FALSE | TRUE |
| *ATF2* | FALSE | FALSE | TRUE |
| *ATG101* | FALSE | FALSE | TRUE |
| *ATG3* | FALSE | FALSE | TRUE |
| *ATG4A* | FALSE | FALSE | TRUE |
| *ATG4C* | FALSE | FALSE | TRUE |
| *ATG5* | FALSE | FALSE | TRUE |

| | | | |
|---|---|---|---|
| *ATL2* | FALSE | FALSE | TRUE |
| *ATP2A2* | FALSE | FALSE | TRUE |
| *ATP2C1* | FALSE | FALSE | TRUE |
| *ATP5A1* | FALSE | FALSE | TRUE |
| *ATP5B* | FALSE | FALSE | TRUE |
| *ATP5C1* | FALSE | FALSE | TRUE |
| *ATP5F1* | FALSE | FALSE | TRUE |
| *ATP5G3* | FALSE | FALSE | TRUE |
| *ATP6V0A1* | FALSE | FALSE | TRUE |
| *ATP6V0E1* | FALSE | FALSE | TRUE |
| *ATP6V1A* | FALSE | FALSE | TRUE |
| *ATP6V1B2* | FALSE | FALSE | TRUE |
| *ATP6V1D* | FALSE | FALSE | TRUE |
| *ATP6V1E1* | FALSE | FALSE | TRUE |
| *ATP6V1F* | FALSE | FALSE | TRUE |
| *ATP6V1H* | FALSE | FALSE | TRUE |
| *AURKA* | FALSE | FALSE | TRUE |
| *AVEN* | FALSE | FALSE | TRUE |
| *AZIN1* | FALSE | FALSE | TRUE |
| *B4GALT2* | FALSE | FALSE | TRUE |
| *B4GALT4* | FALSE | FALSE | TRUE |
| *B4GALT5* | FALSE | FALSE | TRUE |
| *B4GALT7* | FALSE | FALSE | TRUE |
| *BACH2* | FALSE | FALSE | TRUE |
| *BAK1* | FALSE | FALSE | TRUE |
| *BANF1* | FALSE | FALSE | TRUE |
| *BATF* | FALSE | FALSE | TRUE |
| *BBIP1* | FALSE | FALSE | TRUE |
| *BBS10* | FALSE | FALSE | TRUE |
| *BBS7* | FALSE | FALSE | TRUE |
| *BCAS2* | FALSE | FALSE | TRUE |
| *BCL2L11* | FALSE | FALSE | TRUE |
| *BCOR* | FALSE | FALSE | TRUE |
| *BCORL1* | FALSE | FALSE | TRUE |
| *BIK* | FALSE | FALSE | TRUE |
| *BLMH* | FALSE | FALSE | TRUE |
| *BLOC1S2* | FALSE | FALSE | TRUE |
| *BLVRB* | FALSE | FALSE | TRUE |
| *BMP2K* | FALSE | FALSE | TRUE |
| *BNIP3* | FALSE | FALSE | TRUE |
| *BORA* | FALSE | FALSE | TRUE |
| *BRAF* | FALSE | FALSE | TRUE |
| *BRCC3* | FALSE | FALSE | TRUE |
| *BRD7* | FALSE | FALSE | TRUE |

| | | | |
|---|---|---|---|
| *BRI3BP* | FALSE | FALSE | TRUE |
| *BRK1* | FALSE | FALSE | TRUE |
| *BRMS1* | FALSE | FALSE | TRUE |
| *BTF3L4* | FALSE | FALSE | TRUE |
| *BTRC* | FALSE | FALSE | TRUE |
| *BUB1B* | FALSE | FALSE | TRUE |
| *BUD31* | FALSE | FALSE | TRUE |
| *BZW1* | FALSE | FALSE | TRUE |
| *BZW2* | FALSE | FALSE | TRUE |
| *C10orf2* | FALSE | FALSE | TRUE |
| *C11orf24* | FALSE | FALSE | TRUE |
| *C11orf54* | FALSE | FALSE | TRUE |
| *C11orf57* | FALSE | FALSE | TRUE |
| *C11orf58* | FALSE | FALSE | TRUE |
| *C11orf73* | FALSE | FALSE | TRUE |
| *C11orf84* | FALSE | FALSE | TRUE |
| *C11orf95* | FALSE | FALSE | TRUE |
| *C12orf29* | FALSE | FALSE | TRUE |
| *C12orf5* | FALSE | FALSE | TRUE |
| *C14orf142* | FALSE | FALSE | TRUE |
| *C15orf57* | FALSE | FALSE | TRUE |
| *C16orf80* | FALSE | FALSE | TRUE |
| *C17orf85* | FALSE | FALSE | TRUE |
| *C18orf21* | FALSE | FALSE | TRUE |
| *C1D* | FALSE | FALSE | TRUE |
| *C1GALT1C1* | FALSE | FALSE | TRUE |
| *C2orf43* | FALSE | FALSE | TRUE |
| *C2orf47* | FALSE | FALSE | TRUE |
| *C2orf76* | FALSE | FALSE | TRUE |
| *C4orf32* | FALSE | FALSE | TRUE |
| *C5orf22* | FALSE | FALSE | TRUE |
| *C5orf28* | FALSE | FALSE | TRUE |
| *C5orf51* | FALSE | FALSE | TRUE |
| *C6orf203* | FALSE | FALSE | TRUE |
| *C7orf50* | FALSE | FALSE | TRUE |
| *C9orf40* | FALSE | FALSE | TRUE |
| *C9orf64* | FALSE | FALSE | TRUE |
| *C9orf69* | FALSE | FALSE | TRUE |
| *C9orf85* | FALSE | FALSE | TRUE |
| *CAB39* | FALSE | FALSE | TRUE |
| *CABLES1* | FALSE | FALSE | TRUE |
| *CABLES2* | FALSE | FALSE | TRUE |
| *CALR* | FALSE | FALSE | TRUE |
| *CAMK1* | FALSE | FALSE | TRUE |

| | | | |
|---|---|---|---|
| *CAMSAP1* | FALSE | FALSE | TRUE |
| *CAMTA1* | FALSE | FALSE | TRUE |
| *CANT1* | FALSE | FALSE | TRUE |
| *CAPZA1* | FALSE | FALSE | TRUE |
| *CAPZA2* | FALSE | FALSE | TRUE |
| *CASC3* | FALSE | FALSE | TRUE |
| *CBLN3* | FALSE | FALSE | TRUE |
| *CBR4* | FALSE | FALSE | TRUE |
| *CBX6* | FALSE | FALSE | TRUE |
| *CCDC12* | FALSE | FALSE | TRUE |
| *CCDC126* | FALSE | FALSE | TRUE |
| *CCDC127* | FALSE | FALSE | TRUE |
| *CCDC132* | FALSE | FALSE | TRUE |
| *CCDC43* | FALSE | FALSE | TRUE |
| *CCDC59* | FALSE | FALSE | TRUE |
| *CCDC90B* | FALSE | FALSE | TRUE |
| *CCDC94* | FALSE | FALSE | TRUE |
| *CCND2* | FALSE | FALSE | TRUE |
| *CCNH* | FALSE | FALSE | TRUE |
| *CCNI2* | FALSE | FALSE | TRUE |
| *CCNK* | FALSE | FALSE | TRUE |
| *CCNYL1* | FALSE | FALSE | TRUE |
| *CCP110* | FALSE | FALSE | TRUE |
| *CCR4* | FALSE | FALSE | TRUE |
| *CCRN4L* | FALSE | FALSE | TRUE |
| *CCT2* | FALSE | FALSE | TRUE |
| *CCZ1* | FALSE | FALSE | TRUE |
| *CD109* | FALSE | FALSE | TRUE |
| *CD164* | FALSE | FALSE | TRUE |
| *CD2AP* | FALSE | FALSE | TRUE |
| *CD3EAP* | FALSE | FALSE | TRUE |
| *CD40LG* | FALSE | FALSE | TRUE |
| *CD6* | FALSE | FALSE | TRUE |
| *CD82* | FALSE | FALSE | TRUE |
| *CDC23* | FALSE | FALSE | TRUE |
| *CDC34* | FALSE | FALSE | TRUE |
| *CDC5L* | FALSE | FALSE | TRUE |
| *CDCA7L* | FALSE | FALSE | TRUE |
| *CDK1* | FALSE | FALSE | TRUE |
| *CDK2AP2* | FALSE | FALSE | TRUE |
| *CDK8* | FALSE | FALSE | TRUE |
| *CDKAL1* | FALSE | FALSE | TRUE |
| *CDKN1A* | FALSE | FALSE | TRUE |
| *CDKN2AIPNL* | FALSE | FALSE | TRUE |

| | | | |
|---|---|---|---|
| *CDV3* | FALSE | FALSE | TRUE |
| *CEBPG* | FALSE | FALSE | TRUE |
| *CEBPZ* | FALSE | FALSE | TRUE |
| *CENPQ* | FALSE | FALSE | TRUE |
| *CENPW* | FALSE | FALSE | TRUE |
| *CEP104* | FALSE | FALSE | TRUE |
| *CEP19* | FALSE | FALSE | TRUE |
| *CEP41* | FALSE | FALSE | TRUE |
| *CEP57L1* | FALSE | FALSE | TRUE |
| *CEP97* | FALSE | FALSE | TRUE |
| *CEPT1* | FALSE | FALSE | TRUE |
| *CHCHD1* | FALSE | FALSE | TRUE |
| *CHD4* | FALSE | FALSE | TRUE |
| *CHKA* | FALSE | FALSE | TRUE |
| *CHMP1A* | FALSE | FALSE | TRUE |
| *CHMP2A* | FALSE | FALSE | TRUE |
| *CHMP2B* | FALSE | FALSE | TRUE |
| *CHMP5* | FALSE | FALSE | TRUE |
| *CHN1* | FALSE | FALSE | TRUE |
| *CHORDC1* | FALSE | FALSE | TRUE |
| *CHPF* | FALSE | FALSE | TRUE |
| *CHUK* | FALSE | FALSE | TRUE |
| *CIAPIN1* | FALSE | FALSE | TRUE |
| *CINP* | FALSE | FALSE | TRUE |
| *CISD1* | FALSE | FALSE | TRUE |
| *CISD2* | FALSE | FALSE | TRUE |
| *CKS2* | FALSE | FALSE | TRUE |
| *CLDND1* | FALSE | FALSE | TRUE |
| *CLINT1* | FALSE | FALSE | TRUE |
| *CLN8* | FALSE | FALSE | TRUE |
| *CLNS1A* | FALSE | FALSE | TRUE |
| *CLP1* | FALSE | FALSE | TRUE |
| *CLPX* | FALSE | FALSE | TRUE |
| *CLTA* | FALSE | FALSE | TRUE |
| *CLTC* | FALSE | FALSE | TRUE |
| *CMAS* | FALSE | FALSE | TRUE |
| *CMC2* | FALSE | FALSE | TRUE |
| *CMTM6* | FALSE | FALSE | TRUE |
| *CNIH1* | FALSE | FALSE | TRUE |
| *CNIH4* | FALSE | FALSE | TRUE |
| *CNKSR2* | FALSE | FALSE | TRUE |
| *CNOT1* | FALSE | FALSE | TRUE |
| *CNOT10* | FALSE | FALSE | TRUE |
| *CNP* | FALSE | FALSE | TRUE |

| | | | |
|---|---|---|---|
| *COA4* | FALSE | FALSE | TRUE |
| *COMMD8* | FALSE | FALSE | TRUE |
| *COMMD9* | FALSE | FALSE | TRUE |
| *COPB1* | FALSE | FALSE | TRUE |
| *COPB2* | FALSE | FALSE | TRUE |
| *COPS2* | FALSE | FALSE | TRUE |
| *COPS3* | FALSE | FALSE | TRUE |
| *COPS4* | FALSE | FALSE | TRUE |
| *COPS5* | FALSE | FALSE | TRUE |
| *COPS7A* | FALSE | FALSE | TRUE |
| *COPS8* | FALSE | FALSE | TRUE |
| *COQ10B* | FALSE | FALSE | TRUE |
| *COQ9* | FALSE | FALSE | TRUE |
| *CORO1C* | FALSE | FALSE | TRUE |
| *COX17* | FALSE | FALSE | TRUE |
| *CPD* | FALSE | FALSE | TRUE |
| *CPSF3* | FALSE | FALSE | TRUE |
| *CRK* | FALSE | FALSE | TRUE |
| *CRKL* | FALSE | FALSE | TRUE |
| *CRNKL1* | FALSE | FALSE | TRUE |
| *CSK* | FALSE | FALSE | TRUE |
| *CSNK1A1* | FALSE | FALSE | TRUE |
| *CSNK1G3* | FALSE | FALSE | TRUE |
| *CSNK2A1* | FALSE | FALSE | TRUE |
| *CSNK2A2* | FALSE | FALSE | TRUE |
| *CSRNP2* | FALSE | FALSE | TRUE |
| *CSTF1* | FALSE | FALSE | TRUE |
| *CSTF3* | FALSE | FALSE | TRUE |
| *CTH* | FALSE | FALSE | TRUE |
| *CTNS* | FALSE | FALSE | TRUE |
| *CTR9* | FALSE | FALSE | TRUE |
| *CTSL* | FALSE | FALSE | TRUE |
| *CUL2* | FALSE | FALSE | TRUE |
| *CUL3* | FALSE | FALSE | TRUE |
| *CUL5* | FALSE | FALSE | TRUE |
| *CWC15* | FALSE | FALSE | TRUE |
| *CWC22* | FALSE | FALSE | TRUE |
| *CWC25* | FALSE | FALSE | TRUE |
| *CWF19L1* | FALSE | FALSE | TRUE |
| *CXorf40B* | FALSE | FALSE | TRUE |
| *CYB5A* | FALSE | FALSE | TRUE |
| *CYB5RL* | FALSE | FALSE | TRUE |
| *CYCS* | FALSE | FALSE | TRUE |
| *CYP1B1* | FALSE | FALSE | TRUE |

| | | | |
|---|---|---|---|
| *DAD1* | FALSE | FALSE | TRUE |
| *DBT* | FALSE | FALSE | TRUE |
| *DCLRE1A* | FALSE | FALSE | TRUE |
| *DCP1A* | FALSE | FALSE | TRUE |
| *DCPS* | FALSE | FALSE | TRUE |
| *DCTN4* | FALSE | FALSE | TRUE |
| *DCUN1D5* | FALSE | FALSE | TRUE |
| *DDB1* | FALSE | FALSE | TRUE |
| *DDHD1* | FALSE | FALSE | TRUE |
| *DDX19A* | FALSE | FALSE | TRUE |
| *DDX20* | FALSE | FALSE | TRUE |
| *DDX23* | FALSE | FALSE | TRUE |
| *DDX24* | FALSE | FALSE | TRUE |
| *DDX27* | FALSE | FALSE | TRUE |
| *DDX3X* | FALSE | FALSE | TRUE |
| *DDX47* | FALSE | FALSE | TRUE |
| *DDX52* | FALSE | FALSE | TRUE |
| *DDX56* | FALSE | FALSE | TRUE |
| *DENR* | FALSE | FALSE | TRUE |
| *DERL1* | FALSE | FALSE | TRUE |
| *DERL2* | FALSE | FALSE | TRUE |
| *DESI1* | FALSE | FALSE | TRUE |
| *DGAT2* | FALSE | FALSE | TRUE |
| *DGCR8* | FALSE | FALSE | TRUE |
| *DHCR7* | FALSE | FALSE | TRUE |
| *DHODH* | FALSE | FALSE | TRUE |
| *DHX15* | FALSE | FALSE | TRUE |
| *DHX29* | FALSE | FALSE | TRUE |
| *DHX36* | FALSE | FALSE | TRUE |
| *DHX38* | FALSE | FALSE | TRUE |
| *DHX57* | FALSE | FALSE | TRUE |
| *DIABLO* | FALSE | FALSE | TRUE |
| *DIS3* | FALSE | FALSE | TRUE |
| *DLD* | FALSE | FALSE | TRUE |
| *DNAJA1* | FALSE | FALSE | TRUE |
| *DNAJA4* | FALSE | FALSE | TRUE |
| *DNAJB11* | FALSE | FALSE | TRUE |
| *DNAJB12* | FALSE | FALSE | TRUE |
| *DNAJB5* | FALSE | FALSE | TRUE |
| *DNAJB6* | FALSE | FALSE | TRUE |
| *DNAJC14* | FALSE | FALSE | TRUE |
| *DNAJC2* | FALSE | FALSE | TRUE |
| *DNAJC24* | FALSE | FALSE | TRUE |
| *DNAJC7* | FALSE | FALSE | TRUE |

| | | | |
|---|---|---|---|
| *DNAJC8* | FALSE | FALSE | TRUE |
| *DNAJC9* | FALSE | FALSE | TRUE |
| *DNAL1* | FALSE | FALSE | TRUE |
| *DNAL4* | FALSE | FALSE | TRUE |
| *DNM1L* | FALSE | FALSE | TRUE |
| *DNTTIP1* | FALSE | FALSE | TRUE |
| *DNTTIP2* | FALSE | FALSE | TRUE |
| *DONSON* | FALSE | FALSE | TRUE |
| *DPH3* | FALSE | FALSE | TRUE |
| *DPM2* | FALSE | FALSE | TRUE |
| *DPY30* | FALSE | FALSE | TRUE |
| *DR1* | FALSE | FALSE | TRUE |
| *DRG2* | FALSE | FALSE | TRUE |
| *DSCC1* | FALSE | FALSE | TRUE |
| *DTWD1* | FALSE | FALSE | TRUE |
| *DUSP11* | FALSE | FALSE | TRUE |
| *DUSP14* | FALSE | FALSE | TRUE |
| *DUSP3* | FALSE | FALSE | TRUE |
| *DYNC1I2* | FALSE | FALSE | TRUE |
| *DYNLL2* | FALSE | FALSE | TRUE |
| *E2F1* | FALSE | FALSE | TRUE |
| *E2F3* | FALSE | FALSE | TRUE |
| *E2F6* | FALSE | FALSE | TRUE |
| *EAF1* | FALSE | FALSE | TRUE |
| *EAF2* | FALSE | FALSE | TRUE |
| *EARS2* | FALSE | FALSE | TRUE |
| *EBAG9* | FALSE | FALSE | TRUE |
| *ECD* | FALSE | FALSE | TRUE |
| *EFNB1* | FALSE | FALSE | TRUE |
| *EFTUD2* | FALSE | FALSE | TRUE |
| *EHD4* | FALSE | FALSE | TRUE |
| *EI24* | FALSE | FALSE | TRUE |
| *EIF1AX* | FALSE | FALSE | TRUE |
| *EIF1AY* | FALSE | FALSE | TRUE |
| *EIF2S2* | FALSE | FALSE | TRUE |
| *EIF3A* | FALSE | FALSE | TRUE |
| *EIF3J* | FALSE | FALSE | TRUE |
| *EIF3M* | FALSE | FALSE | TRUE |
| *EIF4A3* | FALSE | FALSE | TRUE |
| *EIF4E* | FALSE | FALSE | TRUE |
| *EIF4G2* | FALSE | FALSE | TRUE |
| *EIF4H* | FALSE | FALSE | TRUE |
| *EIF5* | FALSE | FALSE | TRUE |
| *EIF5B* | FALSE | FALSE | TRUE |

| | | | |
|---|---|---|---|
| *EIF6* | FALSE | FALSE | TRUE |
| *ELL* | FALSE | FALSE | TRUE |
| *ELL2* | FALSE | FALSE | TRUE |
| *ELOF1* | FALSE | FALSE | TRUE |
| *ELOVL5* | FALSE | FALSE | TRUE |
| *ELP3* | FALSE | FALSE | TRUE |
| *EMC3* | FALSE | FALSE | TRUE |
| *EMC4* | FALSE | FALSE | TRUE |
| *EMC7* | FALSE | FALSE | TRUE |
| *EMC8* | FALSE | FALSE | TRUE |
| *EMG1* | FALSE | FALSE | TRUE |
| *EMP1* | FALSE | FALSE | TRUE |
| *ENDOG* | FALSE | FALSE | TRUE |
| *ENKD1* | FALSE | FALSE | TRUE |
| *ENPP4* | FALSE | FALSE | TRUE |
| *EPDR1* | FALSE | FALSE | TRUE |
| *ERCC8* | FALSE | FALSE | TRUE |
| *ERF* | FALSE | FALSE | TRUE |
| *ERGIC2* | FALSE | FALSE | TRUE |
| *ERH* | FALSE | FALSE | TRUE |
| *ERI1* | FALSE | FALSE | TRUE |
| *ERI2* | FALSE | FALSE | TRUE |
| *ESCO2* | FALSE | FALSE | TRUE |
| *ESD* | FALSE | FALSE | TRUE |
| *ETF1* | FALSE | FALSE | TRUE |
| *EWSR1* | FALSE | FALSE | TRUE |
| *EXOC5* | FALSE | FALSE | TRUE |
| *EXOSC10* | FALSE | FALSE | TRUE |
| *EXOSC3* | FALSE | FALSE | TRUE |
| *EXOSC4* | FALSE | FALSE | TRUE |
| *EXOSC5* | FALSE | FALSE | TRUE |
| *EXOSC9* | FALSE | FALSE | TRUE |
| *EXTL2* | FALSE | FALSE | TRUE |
| *EYA3* | FALSE | FALSE | TRUE |
| *FADD* | FALSE | FALSE | TRUE |
| *FAF1* | FALSE | FALSE | TRUE |
| *FAIM* | FALSE | FALSE | TRUE |
| *FAM126A* | FALSE | FALSE | TRUE |
| *FAM184A* | FALSE | FALSE | TRUE |
| *FAM207A* | FALSE | FALSE | TRUE |
| *FAM210A* | FALSE | FALSE | TRUE |
| *FAM219A* | FALSE | FALSE | TRUE |
| *FAM35A* | FALSE | FALSE | TRUE |
| *FAM3C* | FALSE | FALSE | TRUE |

| | | | |
|---|---|---|---|
| *FAM49B* | FALSE | FALSE | TRUE |
| *FAM53C* | FALSE | FALSE | TRUE |
| *FAM57A* | FALSE | FALSE | TRUE |
| *FAM58A* | FALSE | FALSE | TRUE |
| *FAM86C2P* | FALSE | FALSE | TRUE |
| *FAM86EP* | FALSE | FALSE | TRUE |
| *FAM89A* | FALSE | FALSE | TRUE |
| *FAM91A1* | FALSE | FALSE | TRUE |
| *FAM98B* | FALSE | FALSE | TRUE |
| *FANCB* | FALSE | FALSE | TRUE |
| *FANCL* | FALSE | FALSE | TRUE |
| *FASN* | FALSE | FALSE | TRUE |
| *FBXL18* | FALSE | FALSE | TRUE |
| *FBXO22* | FALSE | FALSE | TRUE |
| *FBXO28* | FALSE | FALSE | TRUE |
| *FBXO4* | FALSE | FALSE | TRUE |
| *FBXO8* | FALSE | FALSE | TRUE |
| *FBXW11* | FALSE | FALSE | TRUE |
| *FCF1* | FALSE | FALSE | TRUE |
| *FDX1* | FALSE | FALSE | TRUE |
| *FDXACB1* | FALSE | FALSE | TRUE |
| *FER* | FALSE | FALSE | TRUE |
| *FGFR1OP* | FALSE | FALSE | TRUE |
| *FHL3* | FALSE | FALSE | TRUE |
| *FIGNL1* | FALSE | FALSE | TRUE |
| *FIP1L1* | FALSE | FALSE | TRUE |
| *FIZ1* | FALSE | FALSE | TRUE |
| *FOXP4* | FALSE | FALSE | TRUE |
| *FRG1* | FALSE | FALSE | TRUE |
| *FSCN1* | FALSE | FALSE | TRUE |
| *FSTL3* | FALSE | FALSE | TRUE |
| *FUBP1* | FALSE | FALSE | TRUE |
| *FUNDC2* | FALSE | FALSE | TRUE |
| *FXN* | FALSE | FALSE | TRUE |
| *G3BP2* | FALSE | FALSE | TRUE |
| *G6PC3* | FALSE | FALSE | TRUE |
| *GADD45GIP1* | FALSE | FALSE | TRUE |
| *GALK2* | FALSE | FALSE | TRUE |
| *GALNT1* | FALSE | FALSE | TRUE |
| *GALNT7* | FALSE | FALSE | TRUE |
| *GAN* | FALSE | FALSE | TRUE |
| *GAPVD1* | FALSE | FALSE | TRUE |
| *GATC* | FALSE | FALSE | TRUE |
| *GBAS* | FALSE | FALSE | TRUE |

| | | | |
|---|---|---|---|
| *GBF1* | FALSE | FALSE | TRUE |
| *GCA* | FALSE | FALSE | TRUE |
| *GCAT* | FALSE | FALSE | TRUE |
| *GCH1* | FALSE | FALSE | TRUE |
| *GCLC* | FALSE | FALSE | TRUE |
| *GCLM* | FALSE | FALSE | TRUE |
| *GDAP2* | FALSE | FALSE | TRUE |
| *GEMIN7* | FALSE | FALSE | TRUE |
| *GEMIN8* | FALSE | FALSE | TRUE |
| *GEN1* | FALSE | FALSE | TRUE |
| *GFER* | FALSE | FALSE | TRUE |
| *GFOD2* | FALSE | FALSE | TRUE |
| *GFPT1* | FALSE | FALSE | TRUE |
| *GID4* | FALSE | FALSE | TRUE |
| *GINS1* | FALSE | FALSE | TRUE |
| *GK* | FALSE | FALSE | TRUE |
| *GLUD1* | FALSE | FALSE | TRUE |
| *GMEB1* | FALSE | FALSE | TRUE |
| *GMPPB* | FALSE | FALSE | TRUE |
| *GNAI3* | FALSE | FALSE | TRUE |
| *GNL3* | FALSE | FALSE | TRUE |
| *GNL3L* | FALSE | FALSE | TRUE |
| *GNPDA2* | FALSE | FALSE | TRUE |
| *GOLM1* | FALSE | FALSE | TRUE |
| *GOLPH3* | FALSE | FALSE | TRUE |
| *GOLT1B* | FALSE | FALSE | TRUE |
| *GORASP2* | FALSE | FALSE | TRUE |
| *GOSR1* | FALSE | FALSE | TRUE |
| *GOSR2* | FALSE | FALSE | TRUE |
| *GPATCH11* | FALSE | FALSE | TRUE |
| *GPATCH3* | FALSE | FALSE | TRUE |
| *GPC1* | FALSE | FALSE | TRUE |
| *GPD2* | FALSE | FALSE | TRUE |
| *GPN1* | FALSE | FALSE | TRUE |
| *GPN2* | FALSE | FALSE | TRUE |
| *GPR180* | FALSE | FALSE | TRUE |
| *GPR68* | FALSE | FALSE | TRUE |
| *GPT2* | FALSE | FALSE | TRUE |
| *GRPEL1* | FALSE | FALSE | TRUE |
| *GSK3A* | FALSE | FALSE | TRUE |
| *GSKIP* | FALSE | FALSE | TRUE |
| *GSTA4* | FALSE | FALSE | TRUE |
| *GSTO1* | FALSE | FALSE | TRUE |
| *GTF2A2* | FALSE | FALSE | TRUE |

| | | | |
|---|---|---|---|
| *GTF2B* | FALSE | FALSE | TRUE |
| *GTF2E1* | FALSE | FALSE | TRUE |
| *GTF3C1* | FALSE | FALSE | TRUE |
| *GTF3C4* | FALSE | FALSE | TRUE |
| *GTF3C5* | FALSE | FALSE | TRUE |
| *GTPBP10* | FALSE | FALSE | TRUE |
| *GTPBP8* | FALSE | FALSE | TRUE |
| *GUF1* | FALSE | FALSE | TRUE |
| *GZF1* | FALSE | FALSE | TRUE |
| *HACL1* | FALSE | FALSE | TRUE |
| *HADH* | FALSE | FALSE | TRUE |
| *HAT1* | FALSE | FALSE | TRUE |
| *HAUS2* | FALSE | FALSE | TRUE |
| *HBS1L* | FALSE | FALSE | TRUE |
| *HDAC2* | FALSE | FALSE | TRUE |
| *HDDC2* | FALSE | FALSE | TRUE |
| *HEATR2* | FALSE | FALSE | TRUE |
| *HEATR3* | FALSE | FALSE | TRUE |
| *HERPUD1* | FALSE | FALSE | TRUE |
| *HEXIM2* | FALSE | FALSE | TRUE |
| *HIAT1* | FALSE | FALSE | TRUE |
| *HIC1* | FALSE | FALSE | TRUE |
| *HIF1A* | FALSE | FALSE | TRUE |
| *HIGD1A* | FALSE | FALSE | TRUE |
| *HM13* | FALSE | FALSE | TRUE |
| *HMBS* | FALSE | FALSE | TRUE |
| *HMCES* | FALSE | FALSE | TRUE |
| *HMGB3* | FALSE | FALSE | TRUE |
| *HN1* | FALSE | FALSE | TRUE |
| *HNRNPLL* | FALSE | FALSE | TRUE |
| *HNRNPM* | FALSE | FALSE | TRUE |
| *HNRNPU* | FALSE | FALSE | TRUE |
| *HOMEZ* | FALSE | FALSE | TRUE |
| *HPS5* | FALSE | FALSE | TRUE |
| *HS2ST1* | FALSE | FALSE | TRUE |
| *HS6ST1* | FALSE | FALSE | TRUE |
| *HSD17B12* | FALSE | FALSE | TRUE |
| *HSF2* | FALSE | FALSE | TRUE |
| *HSP90AA1* | FALSE | FALSE | TRUE |
| *HSP90AB1* | FALSE | FALSE | TRUE |
| *HSP90B1* | FALSE | FALSE | TRUE |
| *HSPA13* | FALSE | FALSE | TRUE |
| *HSPA14* | FALSE | FALSE | TRUE |
| *HSPA4* | FALSE | FALSE | TRUE |

| | | | |
|---|---|---|---|
| *HSPA5* | FALSE | FALSE | TRUE |
| *HSPA8* | FALSE | FALSE | TRUE |
| *HSPH1* | FALSE | FALSE | TRUE |
| *HTRA2* | FALSE | FALSE | TRUE |
| *HYAL2* | FALSE | FALSE | TRUE |
| *HYAL3* | FALSE | FALSE | TRUE |
| *HYOU1* | FALSE | FALSE | TRUE |
| *IDH3A* | FALSE | FALSE | TRUE |
| *IDH3B* | FALSE | FALSE | TRUE |
| *IDI1* | FALSE | FALSE | TRUE |
| *IER3IP1* | FALSE | FALSE | TRUE |
| *IFNAR1* | FALSE | FALSE | TRUE |
| *IFNLR1* | FALSE | FALSE | TRUE |
| *IFRD1* | FALSE | FALSE | TRUE |
| *IFT43* | FALSE | FALSE | TRUE |
| *IFT46* | FALSE | FALSE | TRUE |
| *IFT57* | FALSE | FALSE | TRUE |
| *IKBIP* | FALSE | FALSE | TRUE |
| *IL2* | FALSE | FALSE | TRUE |
| *IL23A* | FALSE | FALSE | TRUE |
| *IL23R* | FALSE | FALSE | TRUE |
| *IL27RA* | FALSE | FALSE | TRUE |
| *ILF3* | FALSE | FALSE | TRUE |
| *IMMP2L* | FALSE | FALSE | TRUE |
| *IMMT* | FALSE | FALSE | TRUE |
| *IMPA1* | FALSE | FALSE | TRUE |
| *INCENP* | FALSE | FALSE | TRUE |
| *ING5* | FALSE | FALSE | TRUE |
| *INIP* | FALSE | FALSE | TRUE |
| *INO80C* | FALSE | FALSE | TRUE |
| *INTS9* | FALSE | FALSE | TRUE |
| *IP6K1* | FALSE | FALSE | TRUE |
| *IP6K2* | FALSE | FALSE | TRUE |
| *IPO7* | FALSE | FALSE | TRUE |
| *IPP* | FALSE | FALSE | TRUE |
| *IQCB1* | FALSE | FALSE | TRUE |
| *IRGQ* | FALSE | FALSE | TRUE |
| *ISOC1* | FALSE | FALSE | TRUE |
| *ITSN1* | FALSE | FALSE | TRUE |
| *IWS1* | FALSE | FALSE | TRUE |
| *JAM3* | FALSE | FALSE | TRUE |
| *JKAMP* | FALSE | FALSE | TRUE |
| *JMJD6* | FALSE | FALSE | TRUE |
| *KARS* | FALSE | FALSE | TRUE |

| | | | |
|---|---|---|---|
| *KAT5* | FALSE | FALSE | TRUE |
| *KATNA1* | FALSE | FALSE | TRUE |
| *KATNBL1* | FALSE | FALSE | TRUE |
| *KCNK1* | FALSE | FALSE | TRUE |
| *KCNQ5* | FALSE | FALSE | TRUE |
| *KCTD10* | FALSE | FALSE | TRUE |
| *KDELC1* | FALSE | FALSE | TRUE |
| *KDELR2* | FALSE | FALSE | TRUE |
| *KHDRBS1* | FALSE | FALSE | TRUE |
| *KHK* | FALSE | FALSE | TRUE |
| *KIAA0513* | FALSE | FALSE | TRUE |
| *KIAA0930* | FALSE | FALSE | TRUE |
| *KIAA0947* | FALSE | FALSE | TRUE |
| *KIAA1143* | FALSE | FALSE | TRUE |
| *KIAA1429* | FALSE | FALSE | TRUE |
| *KIAA1467* | FALSE | FALSE | TRUE |
| *KIAA1671* | FALSE | FALSE | TRUE |
| *KIF18A* | FALSE | FALSE | TRUE |
| *KIF20B* | FALSE | FALSE | TRUE |
| *KIF5B* | FALSE | FALSE | TRUE |
| *KIN* | FALSE | FALSE | TRUE |
| *KLC2* | FALSE | FALSE | TRUE |
| *KLF5* | FALSE | FALSE | TRUE |
| *KLHL18* | FALSE | FALSE | TRUE |
| *KLHL25* | FALSE | FALSE | TRUE |
| *KPNA2* | FALSE | FALSE | TRUE |
| *KPNA3* | FALSE | FALSE | TRUE |
| *KPNA4* | FALSE | FALSE | TRUE |
| *KRT18* | FALSE | FALSE | TRUE |
| *KSR1* | FALSE | FALSE | TRUE |
| *KTN1* | FALSE | FALSE | TRUE |
| *L2HGDH* | FALSE | FALSE | TRUE |
| *LACTB2* | FALSE | FALSE | TRUE |
| *LAMTOR3* | FALSE | FALSE | TRUE |
| *LAMTOR5* | FALSE | FALSE | TRUE |
| *LAP3* | FALSE | FALSE | TRUE |
| *LARP4B* | FALSE | FALSE | TRUE |
| *LARP7* | FALSE | FALSE | TRUE |
| *LBR* | FALSE | FALSE | TRUE |
| *LCLAT1* | FALSE | FALSE | TRUE |
| *LCP1* | FALSE | FALSE | TRUE |
| *LDHA* | FALSE | FALSE | TRUE |
| *LEO1* | FALSE | FALSE | TRUE |
| *LIF* | FALSE | FALSE | TRUE |

| | | | |
|---|---|---|---|
| *LIN52* | FALSE | FALSE | TRUE |
| *LIN7C* | FALSE | FALSE | TRUE |
| *LINC00152* | FALSE | FALSE | TRUE |
| *LINC00998* | FALSE | FALSE | TRUE |
| *LMAN2L* | FALSE | FALSE | TRUE |
| *LMBR1* | FALSE | FALSE | TRUE |
| *LOC100288842* | FALSE | FALSE | TRUE |
| *LOC100505812* | FALSE | FALSE | TRUE |
| *LOC339803* | FALSE | FALSE | TRUE |
| *LOH12CR1* | FALSE | FALSE | TRUE |
| *LPCAT3* | FALSE | FALSE | TRUE |
| *LPXN* | FALSE | FALSE | TRUE |
| *LRCH1* | FALSE | FALSE | TRUE |
| *LRP11* | FALSE | FALSE | TRUE |
| *LRPAP1* | FALSE | FALSE | TRUE |
| *LRRC20* | FALSE | FALSE | TRUE |
| *LRRC40* | FALSE | FALSE | TRUE |
| *LRRC41* | FALSE | FALSE | TRUE |
| *LRRC58* | FALSE | FALSE | TRUE |
| *LRRC61* | FALSE | FALSE | TRUE |
| *LRRC8C* | FALSE | FALSE | TRUE |
| *LRRFIP1* | FALSE | FALSE | TRUE |
| *LRTOMT* | FALSE | FALSE | TRUE |
| *LRWD1* | FALSE | FALSE | TRUE |
| *LSM1* | FALSE | FALSE | TRUE |
| *LSM3* | FALSE | FALSE | TRUE |
| *LSM4* | FALSE | FALSE | TRUE |
| *LSM5* | FALSE | FALSE | TRUE |
| *LSM6* | FALSE | FALSE | TRUE |
| *LYAR* | FALSE | FALSE | TRUE |
| *LYPLA1* | FALSE | FALSE | TRUE |
| *MAFG* | FALSE | FALSE | TRUE |
| *MAGOH* | FALSE | FALSE | TRUE |
| *MAGOHB* | FALSE | FALSE | TRUE |
| *MALSU1* | FALSE | FALSE | TRUE |
| *MAMLD1* | FALSE | FALSE | TRUE |
| *MANBAL* | FALSE | FALSE | TRUE |
| *MAPK1* | FALSE | FALSE | TRUE |
| *MAPK1IP1L* | FALSE | FALSE | TRUE |
| *MAPK9* | FALSE | FALSE | TRUE |
| *MAPKAPK5* | FALSE | FALSE | TRUE |
| *MAPRE1* | FALSE | FALSE | TRUE |
| *5-Mar* | FALSE | FALSE | TRUE |
| *MARS* | FALSE | FALSE | TRUE |

| | | | |
|---|---|---|---|
| *MB21D1* | FALSE | FALSE | TRUE |
| *MBD3* | FALSE | FALSE | TRUE |
| *MBD4* | FALSE | FALSE | TRUE |
| *MCCC2* | FALSE | FALSE | TRUE |
| *MCFD2* | FALSE | FALSE | TRUE |
| *MCM8* | FALSE | FALSE | TRUE |
| *MCRS1* | FALSE | FALSE | TRUE |
| *MCUR1* | FALSE | FALSE | TRUE |
| *MDFIC* | FALSE | FALSE | TRUE |
| *MECR* | FALSE | FALSE | TRUE |
| *MED1* | FALSE | FALSE | TRUE |
| *MED14* | FALSE | FALSE | TRUE |
| *MED19* | FALSE | FALSE | TRUE |
| *MED27* | FALSE | FALSE | TRUE |
| *MED30* | FALSE | FALSE | TRUE |
| *MED7* | FALSE | FALSE | TRUE |
| *MESDC2* | FALSE | FALSE | TRUE |
| *METAP2* | FALSE | FALSE | TRUE |
| *METRNL* | FALSE | FALSE | TRUE |
| *METTL1* | FALSE | FALSE | TRUE |
| *METTL14* | FALSE | FALSE | TRUE |
| *METTL16* | FALSE | FALSE | TRUE |
| *METTL2A* | FALSE | FALSE | TRUE |
| *METTL6* | FALSE | FALSE | TRUE |
| *MEX3D* | FALSE | FALSE | TRUE |
| *MFAP3* | FALSE | FALSE | TRUE |
| *MFN1* | FALSE | FALSE | TRUE |
| *MFSD12* | FALSE | FALSE | TRUE |
| *MFSD5* | FALSE | FALSE | TRUE |
| *MFSD6* | FALSE | FALSE | TRUE |
| *MGME1* | FALSE | FALSE | TRUE |
| *MIEF1* | FALSE | FALSE | TRUE |
| *MIEN1* | FALSE | FALSE | TRUE |
| *MIER3* | FALSE | FALSE | TRUE |
| *MINPP1* | FALSE | FALSE | TRUE |
| *MITD1* | FALSE | FALSE | TRUE |
| *MKKS* | FALSE | FALSE | TRUE |
| *MKRN2* | FALSE | FALSE | TRUE |
| *MLEC* | FALSE | FALSE | TRUE |
| *MLLT1* | FALSE | FALSE | TRUE |
| *MMGT1* | FALSE | FALSE | TRUE |
| *MND1* | FALSE | FALSE | TRUE |
| *MOB1A* | FALSE | FALSE | TRUE |
| *MOB3A* | FALSE | FALSE | TRUE |

| | | | |
|---|---|---|---|
| *MOGS* | FALSE | FALSE | TRUE |
| *MON1A* | FALSE | FALSE | TRUE |
| *MORN2* | FALSE | FALSE | TRUE |
| *MORN4* | FALSE | FALSE | TRUE |
| *MOSPD1* | FALSE | FALSE | TRUE |
| *MPHOSPH10* | FALSE | FALSE | TRUE |
| *MPHOSPH6* | FALSE | FALSE | TRUE |
| *MPLKIP* | FALSE | FALSE | TRUE |
| *MRPL1* | FALSE | FALSE | TRUE |
| *MRPL13* | FALSE | FALSE | TRUE |
| *MRPL14* | FALSE | FALSE | TRUE |
| *MRPL17* | FALSE | FALSE | TRUE |
| *MRPL18* | FALSE | FALSE | TRUE |
| *MRPL19* | FALSE | FALSE | TRUE |
| *MRPL42* | FALSE | FALSE | TRUE |
| *MRPL46* | FALSE | FALSE | TRUE |
| *MRPL47* | FALSE | FALSE | TRUE |
| *MRPL50* | FALSE | FALSE | TRUE |
| *MRPL57* | FALSE | FALSE | TRUE |
| *MRPS10* | FALSE | FALSE | TRUE |
| *MRPS12* | FALSE | FALSE | TRUE |
| *MRPS18A* | FALSE | FALSE | TRUE |
| *MRPS22* | FALSE | FALSE | TRUE |
| *MRPS26* | FALSE | FALSE | TRUE |
| *MRPS27* | FALSE | FALSE | TRUE |
| *MRPS28* | FALSE | FALSE | TRUE |
| *MRPS33* | FALSE | FALSE | TRUE |
| *MRPS35* | FALSE | FALSE | TRUE |
| *MRPS6* | FALSE | FALSE | TRUE |
| *MRPS7* | FALSE | FALSE | TRUE |
| *MSANTD2* | FALSE | FALSE | TRUE |
| *MSC* | FALSE | FALSE | TRUE |
| *MSRB1* | FALSE | FALSE | TRUE |
| *MT1X* | FALSE | FALSE | TRUE |
| *MTAP* | FALSE | FALSE | TRUE |
| *MTDH* | FALSE | FALSE | TRUE |
| *MTERF* | FALSE | FALSE | TRUE |
| *MTERFD2* | FALSE | FALSE | TRUE |
| *MTF1* | FALSE | FALSE | TRUE |
| *MTFMT* | FALSE | FALSE | TRUE |
| *MTFP1* | FALSE | FALSE | TRUE |
| *MTG2* | FALSE | FALSE | TRUE |
| *MTRF1L* | FALSE | FALSE | TRUE |
| *MTX2* | FALSE | FALSE | TRUE |

| | | | |
|---|---|---|---|
| *MUT* | FALSE | FALSE | TRUE |
| *MVK* | FALSE | FALSE | TRUE |
| *MYL6B* | FALSE | FALSE | TRUE |
| *MYNN* | FALSE | FALSE | TRUE |
| *MYOF* | FALSE | FALSE | TRUE |
| *MZT1* | FALSE | FALSE | TRUE |
| *N4BP3* | FALSE | FALSE | TRUE |
| *NAA35* | FALSE | FALSE | TRUE |
| *NAA50* | FALSE | FALSE | TRUE |
| *NABP1* | FALSE | FALSE | TRUE |
| *NACC1* | FALSE | FALSE | TRUE |
| *NADK* | FALSE | FALSE | TRUE |
| *NAE1* | FALSE | FALSE | TRUE |
| *NAF1* | FALSE | FALSE | TRUE |
| *NANS* | FALSE | FALSE | TRUE |
| *NAP1L4* | FALSE | FALSE | TRUE |
| *NAPEPLD* | FALSE | FALSE | TRUE |
| *NAPG* | FALSE | FALSE | TRUE |
| *NARS* | FALSE | FALSE | TRUE |
| *NASP* | FALSE | FALSE | TRUE |
| *NCAPH* | FALSE | FALSE | TRUE |
| *NCBP1* | FALSE | FALSE | TRUE |
| *NCEH1* | FALSE | FALSE | TRUE |
| *NCOA2* | FALSE | FALSE | TRUE |
| *NCOA3* | FALSE | FALSE | TRUE |
| *NCOA5* | FALSE | FALSE | TRUE |
| *NDC1* | FALSE | FALSE | TRUE |
| *NDFIP2* | FALSE | FALSE | TRUE |
| *NDUFA6* | FALSE | FALSE | TRUE |
| *NDUFA8* | FALSE | FALSE | TRUE |
| *NDUFA9* | FALSE | FALSE | TRUE |
| *NDUFAB1* | FALSE | FALSE | TRUE |
| *NDUFAF1* | FALSE | FALSE | TRUE |
| *NDUFAF3* | FALSE | FALSE | TRUE |
| *NDUFAF4* | FALSE | FALSE | TRUE |
| *NDUFAF5* | FALSE | FALSE | TRUE |
| *NDUFC1* | FALSE | FALSE | TRUE |
| *NDUFS3* | FALSE | FALSE | TRUE |
| *NEDD4* | FALSE | FALSE | TRUE |
| *NEK4* | FALSE | FALSE | TRUE |
| *NELFA* | FALSE | FALSE | TRUE |
| *NETO2* | FALSE | FALSE | TRUE |
| *NFE2L2* | FALSE | FALSE | TRUE |
| *NFE2L3* | FALSE | FALSE | TRUE |

| | | | |
|---|---|---|---|
| *NFIX* | FALSE | FALSE | TRUE |
| *NFKB1* | FALSE | FALSE | TRUE |
| *NFKBIE* | FALSE | FALSE | TRUE |
| *NGLY1* | FALSE | FALSE | TRUE |
| *NGRN* | FALSE | FALSE | TRUE |
| *NHP2L1* | FALSE | FALSE | TRUE |
| *NINJ1* | FALSE | FALSE | TRUE |
| *NIPA2* | FALSE | FALSE | TRUE |
| *NIPAL4* | FALSE | FALSE | TRUE |
| *NKIRAS1* | FALSE | FALSE | TRUE |
| *NKIRAS2* | FALSE | FALSE | TRUE |
| *NLRP2* | FALSE | FALSE | TRUE |
| *NMNAT1* | FALSE | FALSE | TRUE |
| *NMT1* | FALSE | FALSE | TRUE |
| *NNT-AS1* | FALSE | FALSE | TRUE |
| *NOL11* | FALSE | FALSE | TRUE |
| *NOL7* | FALSE | FALSE | TRUE |
| *NOL9* | FALSE | FALSE | TRUE |
| *NOP10* | FALSE | FALSE | TRUE |
| *NOP16* | FALSE | FALSE | TRUE |
| *NOP2* | FALSE | FALSE | TRUE |
| *NOP56* | FALSE | FALSE | TRUE |
| *NOP58* | FALSE | FALSE | TRUE |
| *NPC1* | FALSE | FALSE | TRUE |
| *NQO2* | FALSE | FALSE | TRUE |
| *NR4A3* | FALSE | FALSE | TRUE |
| *NRAS* | FALSE | FALSE | TRUE |
| *NRBP1* | FALSE | FALSE | TRUE |
| *NSFL1C* | FALSE | FALSE | TRUE |
| *NSMCE1* | FALSE | FALSE | TRUE |
| *NSRP1* | FALSE | FALSE | TRUE |
| *NSUN4* | FALSE | FALSE | TRUE |
| *NTMT1* | FALSE | FALSE | TRUE |
| *NUBP2* | FALSE | FALSE | TRUE |
| *NUDC* | FALSE | FALSE | TRUE |
| *NUDCD1* | FALSE | FALSE | TRUE |
| *NUDCD2* | FALSE | FALSE | TRUE |
| *NUDT2* | FALSE | FALSE | TRUE |
| *NUDT21* | FALSE | FALSE | TRUE |
| *NUP133* | FALSE | FALSE | TRUE |
| *NUP153* | FALSE | FALSE | TRUE |
| *NUP155* | FALSE | FALSE | TRUE |
| *NUP50* | FALSE | FALSE | TRUE |
| *NUP54* | FALSE | FALSE | TRUE |

| | | | |
|---|---|---|---|
| *NUP88* | FALSE | FALSE | TRUE |
| *NUP98* | FALSE | FALSE | TRUE |
| *NUS1* | FALSE | FALSE | TRUE |
| *NUSAP1* | FALSE | FALSE | TRUE |
| *NUTM2A-AS1* | FALSE | FALSE | TRUE |
| *NXT1* | FALSE | FALSE | TRUE |
| *OAT* | FALSE | FALSE | TRUE |
| *OPA1* | FALSE | FALSE | TRUE |
| *OPA3* | FALSE | FALSE | TRUE |
| *OSGEP* | FALSE | FALSE | TRUE |
| *OSM* | FALSE | FALSE | TRUE |
| *OSTC* | FALSE | FALSE | TRUE |
| *OXSR1* | FALSE | FALSE | TRUE |
| *PAK4* | FALSE | FALSE | TRUE |
| *PAM* | FALSE | FALSE | TRUE |
| *PANK2* | FALSE | FALSE | TRUE |
| *PAPOLA* | FALSE | FALSE | TRUE |
| *PAPSS1* | FALSE | FALSE | TRUE |
| *PAQR3* | FALSE | FALSE | TRUE |
| *PARL* | FALSE | FALSE | TRUE |
| *PARVB* | FALSE | FALSE | TRUE |
| *PATL1* | FALSE | FALSE | TRUE |
| *PAXBP1* | FALSE | FALSE | TRUE |
| *PBDC1* | FALSE | FALSE | TRUE |
| *PCGF1* | FALSE | FALSE | TRUE |
| *PCK2* | FALSE | FALSE | TRUE |
| *PCMT1* | FALSE | FALSE | TRUE |
| *PCNA* | FALSE | FALSE | TRUE |
| *PDCD1* | FALSE | FALSE | TRUE |
| *PDCD6* | FALSE | FALSE | TRUE |
| *PDCL* | FALSE | FALSE | TRUE |
| *PDCL3* | FALSE | FALSE | TRUE |
| *PDE12* | FALSE | FALSE | TRUE |
| *PDHB* | FALSE | FALSE | TRUE |
| *PDHX* | FALSE | FALSE | TRUE |
| *PDIA4* | FALSE | FALSE | TRUE |
| *PDS5A* | FALSE | FALSE | TRUE |
| *PDXK* | FALSE | FALSE | TRUE |
| *PDXP* | FALSE | FALSE | TRUE |
| *PEAK1* | FALSE | FALSE | TRUE |
| *PER2* | FALSE | FALSE | TRUE |
| *PEX2* | FALSE | FALSE | TRUE |
| *PEX26* | FALSE | FALSE | TRUE |
| *PEX3* | FALSE | FALSE | TRUE |

| | | | |
|---|---|---|---|
| *PFDN1* | FALSE | FALSE | TRUE |
| *PFDN4* | FALSE | FALSE | TRUE |
| *PFKFB3* | FALSE | FALSE | TRUE |
| *PGAM1* | FALSE | FALSE | TRUE |
| *PGAP1* | FALSE | FALSE | TRUE |
| *PGRMC1* | FALSE | FALSE | TRUE |
| *PHLDA1* | FALSE | FALSE | TRUE |
| *PHTF1* | FALSE | FALSE | TRUE |
| *PHYH* | FALSE | FALSE | TRUE |
| *PIM2* | FALSE | FALSE | TRUE |
| *PITHD1* | FALSE | FALSE | TRUE |
| *PITPNB* | FALSE | FALSE | TRUE |
| *PLAA* | FALSE | FALSE | TRUE |
| *PLEKHB2* | FALSE | FALSE | TRUE |
| *PLGRKT* | FALSE | FALSE | TRUE |
| *PMPCA* | FALSE | FALSE | TRUE |
| *PMS1* | FALSE | FALSE | TRUE |
| *PMS2* | FALSE | FALSE | TRUE |
| *PNP* | FALSE | FALSE | TRUE |
| *POC1A* | FALSE | FALSE | TRUE |
| *POLB* | FALSE | FALSE | TRUE |
| *POLD3* | FALSE | FALSE | TRUE |
| *POLE3* | FALSE | FALSE | TRUE |
| *POLR2A* | FALSE | FALSE | TRUE |
| *POLR2B* | FALSE | FALSE | TRUE |
| *POLR2C* | FALSE | FALSE | TRUE |
| *POLR3E* | FALSE | FALSE | TRUE |
| *POLR3H* | FALSE | FALSE | TRUE |
| *POLR3K* | FALSE | FALSE | TRUE |
| *POMGNT2* | FALSE | FALSE | TRUE |
| *POMP* | FALSE | FALSE | TRUE |
| *POMT2* | FALSE | FALSE | TRUE |
| *POP7* | FALSE | FALSE | TRUE |
| *POU2F2* | FALSE | FALSE | TRUE |
| *PPAPDC1B* | FALSE | FALSE | TRUE |
| *PPAT* | FALSE | FALSE | TRUE |
| *PPFIA1* | FALSE | FALSE | TRUE |
| *PPIL3* | FALSE | FALSE | TRUE |
| *PPIL4* | FALSE | FALSE | TRUE |
| *PPM1G* | FALSE | FALSE | TRUE |
| *PPME1* | FALSE | FALSE | TRUE |
| *PPP1CB* | FALSE | FALSE | TRUE |
| *PPP1R15A* | FALSE | FALSE | TRUE |
| *PPP1R16B* | FALSE | FALSE | TRUE |

| | | | |
|---|---|---|---|
| *PPP1R8* | FALSE | FALSE | TRUE |
| *PPP2CA* | FALSE | FALSE | TRUE |
| *PPP2R1A* | FALSE | FALSE | TRUE |
| *PPP2R1B* | FALSE | FALSE | TRUE |
| *PPP2R2A* | FALSE | FALSE | TRUE |
| *PPTC7* | FALSE | FALSE | TRUE |
| *PQLC1* | FALSE | FALSE | TRUE |
| *PRADC1* | FALSE | FALSE | TRUE |
| *PRDM4* | FALSE | FALSE | TRUE |
| *PREB* | FALSE | FALSE | TRUE |
| *PRELID1* | FALSE | FALSE | TRUE |
| *PREPL* | FALSE | FALSE | TRUE |
| *PRKAR2A* | FALSE | FALSE | TRUE |
| *PRKCD* | FALSE | FALSE | TRUE |
| *PRPF4* | FALSE | FALSE | TRUE |
| *PRPF40A* | FALSE | FALSE | TRUE |
| *PRPF8* | FALSE | FALSE | TRUE |
| *PSAT1* | FALSE | FALSE | TRUE |
| *PSMA1* | FALSE | FALSE | TRUE |
| *PSMA2* | FALSE | FALSE | TRUE |
| *PSMA3* | FALSE | FALSE | TRUE |
| *PSMA4* | FALSE | FALSE | TRUE |
| *PSMA6* | FALSE | FALSE | TRUE |
| *PSMB7* | FALSE | FALSE | TRUE |
| *PSMC3IP* | FALSE | FALSE | TRUE |
| *PSMC4* | FALSE | FALSE | TRUE |
| *PSMC5* | FALSE | FALSE | TRUE |
| *PSMC6* | FALSE | FALSE | TRUE |
| *PSMD10* | FALSE | FALSE | TRUE |
| *PSMD3* | FALSE | FALSE | TRUE |
| *PSMD8* | FALSE | FALSE | TRUE |
| *PSMD9* | FALSE | FALSE | TRUE |
| *PSME4* | FALSE | FALSE | TRUE |
| *PSMF1* | FALSE | FALSE | TRUE |
| *PSMG1* | FALSE | FALSE | TRUE |
| *PSMG2* | FALSE | FALSE | TRUE |
| *PSPC1* | FALSE | FALSE | TRUE |
| *PTCD2* | FALSE | FALSE | TRUE |
| *PTER* | FALSE | FALSE | TRUE |
| *PTGES2* | FALSE | FALSE | TRUE |
| *PTGES3* | FALSE | FALSE | TRUE |
| *PTPN1* | FALSE | FALSE | TRUE |
| *PTRH1* | FALSE | FALSE | TRUE |
| *PTS* | FALSE | FALSE | TRUE |

| | | | |
|---|---|---|---|
| *PTTG1* | FALSE | FALSE | TRUE |
| *PUF60* | FALSE | FALSE | TRUE |
| *PUS1* | FALSE | FALSE | TRUE |
| *PXDC1* | FALSE | FALSE | TRUE |
| *PYCR1* | FALSE | FALSE | TRUE |
| *PYCRL* | FALSE | FALSE | TRUE |
| *QPCTL* | FALSE | FALSE | TRUE |
| *QTRTD1* | FALSE | FALSE | TRUE |
| *RAB10* | FALSE | FALSE | TRUE |
| *RAB11A* | FALSE | FALSE | TRUE |
| *RAB14* | FALSE | FALSE | TRUE |
| *RAB1A* | FALSE | FALSE | TRUE |
| *RAB21* | FALSE | FALSE | TRUE |
| *RAB27A* | FALSE | FALSE | TRUE |
| *RAB28* | FALSE | FALSE | TRUE |
| *RAB35* | FALSE | FALSE | TRUE |
| *RAB5A* | FALSE | FALSE | TRUE |
| *RAB7A* | FALSE | FALSE | TRUE |
| *RAB8B* | FALSE | FALSE | TRUE |
| *RABEP1* | FALSE | FALSE | TRUE |
| *RAD1* | FALSE | FALSE | TRUE |
| *RAD23B* | FALSE | FALSE | TRUE |
| *RAD51* | FALSE | FALSE | TRUE |
| *RAD51AP1* | FALSE | FALSE | TRUE |
| *RAI14* | FALSE | FALSE | TRUE |
| *RALA* | FALSE | FALSE | TRUE |
| *RANBP2* | FALSE | FALSE | TRUE |
| *RANBP3* | FALSE | FALSE | TRUE |
| *RANGAP1* | FALSE | FALSE | TRUE |
| *RAP1A* | FALSE | FALSE | TRUE |
| *RAPGEF2* | FALSE | FALSE | TRUE |
| *RBBP7* | FALSE | FALSE | TRUE |
| *RBL1* | FALSE | FALSE | TRUE |
| *RBM10* | FALSE | FALSE | TRUE |
| *RBM12B* | FALSE | FALSE | TRUE |
| *RBM17* | FALSE | FALSE | TRUE |
| *RBM18* | FALSE | FALSE | TRUE |
| *RBM22* | FALSE | FALSE | TRUE |
| *RBM27* | FALSE | FALSE | TRUE |
| *RBM42* | FALSE | FALSE | TRUE |
| *RBPJ* | FALSE | FALSE | TRUE |
| *RCN1* | FALSE | FALSE | TRUE |
| *RDH10* | FALSE | FALSE | TRUE |
| *RDH13* | FALSE | FALSE | TRUE |

| | | | |
|---|---|---|---|
| *REL* | FALSE | FALSE | TRUE |
| *RELB* | FALSE | FALSE | TRUE |
| *RELT* | FALSE | FALSE | TRUE |
| *REXO2* | FALSE | FALSE | TRUE |
| *RFESD* | FALSE | FALSE | TRUE |
| *RFK* | FALSE | FALSE | TRUE |
| *RFTN1* | FALSE | FALSE | TRUE |
| *RGCC* | FALSE | FALSE | TRUE |
| *RHOG* | FALSE | FALSE | TRUE |
| *RILPL2* | FALSE | FALSE | TRUE |
| *RINT1* | FALSE | FALSE | TRUE |
| *RIOK2* | FALSE | FALSE | TRUE |
| *RNF121* | FALSE | FALSE | TRUE |
| *RNF185* | FALSE | FALSE | TRUE |
| *RNF19A* | FALSE | FALSE | TRUE |
| *RNF4* | FALSE | FALSE | TRUE |
| *RNF41* | FALSE | FALSE | TRUE |
| *RNF8* | FALSE | FALSE | TRUE |
| *RNMT* | FALSE | FALSE | TRUE |
| *RNMTL1* | FALSE | FALSE | TRUE |
| *RP9* | FALSE | FALSE | TRUE |
| *RPA3* | FALSE | FALSE | TRUE |
| *RPF1* | FALSE | FALSE | TRUE |
| *RPL23AP53* | FALSE | FALSE | TRUE |
| *RPL23AP82* | FALSE | FALSE | TRUE |
| *RPL26L1* | FALSE | FALSE | TRUE |
| *RPN1* | FALSE | FALSE | TRUE |
| *RPP25L* | FALSE | FALSE | TRUE |
| *RPS19BP1* | FALSE | FALSE | TRUE |
| *RPS6KA2* | FALSE | FALSE | TRUE |
| *RPS6KA5* | FALSE | FALSE | TRUE |
| *RPUSD3* | FALSE | FALSE | TRUE |
| *RRAGC* | FALSE | FALSE | TRUE |
| *RRM1* | FALSE | FALSE | TRUE |
| *RRM2B* | FALSE | FALSE | TRUE |
| *RRP12* | FALSE | FALSE | TRUE |
| *RRP15* | FALSE | FALSE | TRUE |
| *RSL24D1* | FALSE | FALSE | TRUE |
| *RSPRY1* | FALSE | FALSE | TRUE |
| *RSRC2* | FALSE | FALSE | TRUE |
| *RTFDC1* | FALSE | FALSE | TRUE |
| *RWDD1* | FALSE | FALSE | TRUE |
| *S1PR2* | FALSE | FALSE | TRUE |
| *SAC3D1* | FALSE | FALSE | TRUE |

| | | | |
|---|---|---|---|
| *SAFB* | FALSE | FALSE | TRUE |
| *SAMD4A* | FALSE | FALSE | TRUE |
| *SAMD4B* | FALSE | FALSE | TRUE |
| *SAMM50* | FALSE | FALSE | TRUE |
| *SAMSN1* | FALSE | FALSE | TRUE |
| *SAP18* | FALSE | FALSE | TRUE |
| *SAR1B* | FALSE | FALSE | TRUE |
| *SARNP* | FALSE | FALSE | TRUE |
| *SART3* | FALSE | FALSE | TRUE |
| *SASS6* | FALSE | FALSE | TRUE |
| *SBF2-AS1* | FALSE | FALSE | TRUE |
| *SBNO1* | FALSE | FALSE | TRUE |
| *SBNO2* | FALSE | FALSE | TRUE |
| *SCAF1* | FALSE | FALSE | TRUE |
| *SCML2* | FALSE | FALSE | TRUE |
| *SCYL1* | FALSE | FALSE | TRUE |
| *SCYL2* | FALSE | FALSE | TRUE |
| *SDE2* | FALSE | FALSE | TRUE |
| *SDHAF2* | FALSE | FALSE | TRUE |
| *SDHB* | FALSE | FALSE | TRUE |
| *SEC14L1* | FALSE | FALSE | TRUE |
| *SEC23B* | FALSE | FALSE | TRUE |
| *SEC24C* | FALSE | FALSE | TRUE |
| *SEC61A1* | FALSE | FALSE | TRUE |
| *SEC61B* | FALSE | FALSE | TRUE |
| *SEC63* | FALSE | FALSE | TRUE |
| *SELK* | FALSE | FALSE | TRUE |
| *SENP2* | FALSE | FALSE | TRUE |
| *8-Sep* | FALSE | FALSE | TRUE |
| *SERAC1* | FALSE | FALSE | TRUE |
| *SERPINH1* | FALSE | FALSE | TRUE |
| *SESN2* | FALSE | FALSE | TRUE |
| *SETMAR* | FALSE | FALSE | TRUE |
| *SF3B2* | FALSE | FALSE | TRUE |
| *SF3B6* | FALSE | FALSE | TRUE |
| *SFT2D1* | FALSE | FALSE | TRUE |
| *SFXN5* | FALSE | FALSE | TRUE |
| *SGK223* | FALSE | FALSE | TRUE |
| *SGPP2* | FALSE | FALSE | TRUE |
| *SH2D4A* | FALSE | FALSE | TRUE |
| *SH3BGR* | FALSE | FALSE | TRUE |
| *SHMT1* | FALSE | FALSE | TRUE |
| *SHOC2* | FALSE | FALSE | TRUE |
| *SIKE1* | FALSE | FALSE | TRUE |

| | | | |
|---|---|---|---|
| *SKIL* | FALSE | FALSE | TRUE |
| *SKIV2L2* | FALSE | FALSE | TRUE |
| *SLBP* | FALSE | FALSE | TRUE |
| *SLC16A1* | FALSE | FALSE | TRUE |
| *SLC16A13* | FALSE | FALSE | TRUE |
| *SLC19A1* | FALSE | FALSE | TRUE |
| *SLC1A4* | FALSE | FALSE | TRUE |
| *SLC20A1* | FALSE | FALSE | TRUE |
| *SLC25A17* | FALSE | FALSE | TRUE |
| *SLC25A33* | FALSE | FALSE | TRUE |
| *SLC25A5* | FALSE | FALSE | TRUE |
| *SLC25A51* | FALSE | FALSE | TRUE |
| *SLC26A2* | FALSE | FALSE | TRUE |
| *SLC30A5* | FALSE | FALSE | TRUE |
| *SLC30A7* | FALSE | FALSE | TRUE |
| *SLC30A9* | FALSE | FALSE | TRUE |
| *SLC31A1* | FALSE | FALSE | TRUE |
| *SLC35C1* | FALSE | FALSE | TRUE |
| *SLC35D1* | FALSE | FALSE | TRUE |
| *SLC35E1* | FALSE | FALSE | TRUE |
| *SLC35E4* | FALSE | FALSE | TRUE |
| *SLC36A4* | FALSE | FALSE | TRUE |
| *SLC39A11* | FALSE | FALSE | TRUE |
| *SLC39A8* | FALSE | FALSE | TRUE |
| *SLC4A1AP* | FALSE | FALSE | TRUE |
| *SLC52A2* | FALSE | FALSE | TRUE |
| *SLC5A6* | FALSE | FALSE | TRUE |
| *SLC7A1* | FALSE | FALSE | TRUE |
| *SLC7A5* | FALSE | FALSE | TRUE |
| *SLC9A7* | FALSE | FALSE | TRUE |
| *SLCO4A1* | FALSE | FALSE | TRUE |
| *SLIRP* | FALSE | FALSE | TRUE |
| *SMAD2* | FALSE | FALSE | TRUE |
| *SMARCA5* | FALSE | FALSE | TRUE |
| *SMC3* | FALSE | FALSE | TRUE |
| *SMC4* | FALSE | FALSE | TRUE |
| *SMC5* | FALSE | FALSE | TRUE |
| *SMCO4* | FALSE | FALSE | TRUE |
| *SMIM12* | FALSE | FALSE | TRUE |
| *SMIM13* | FALSE | FALSE | TRUE |
| *SMIM3* | FALSE | FALSE | TRUE |
| *SMIM7* | FALSE | FALSE | TRUE |
| *SMIM8* | FALSE | FALSE | TRUE |
| *SMKR1* | FALSE | FALSE | TRUE |

| | | | |
|---|---|---|---|
| *SMNDC1* | FALSE | FALSE | TRUE |
| *SMPD2* | FALSE | FALSE | TRUE |
| *SMU1* | FALSE | FALSE | TRUE |
| *SMUG1* | FALSE | FALSE | TRUE |
| *SMYD5* | FALSE | FALSE | TRUE |
| *SNAPC1* | FALSE | FALSE | TRUE |
| *SNAPC2* | FALSE | FALSE | TRUE |
| *SNIP1* | FALSE | FALSE | TRUE |
| *SNRPB2* | FALSE | FALSE | TRUE |
| *SNW1* | FALSE | FALSE | TRUE |
| *SNX11* | FALSE | FALSE | TRUE |
| *SNX16* | FALSE | FALSE | TRUE |
| *SNX25* | FALSE | FALSE | TRUE |
| *SNX3* | FALSE | FALSE | TRUE |
| *SNX8* | FALSE | FALSE | TRUE |
| *SNX9* | FALSE | FALSE | TRUE |
| *SOCS4* | FALSE | FALSE | TRUE |
| *SOCS7* | FALSE | FALSE | TRUE |
| *SPATA2* | FALSE | FALSE | TRUE |
| *SPATA2L* | FALSE | FALSE | TRUE |
| *SPATS2L* | FALSE | FALSE | TRUE |
| *SPHK1* | FALSE | FALSE | TRUE |
| *SPHK2* | FALSE | FALSE | TRUE |
| *SPIRE1* | FALSE | FALSE | TRUE |
| *SPRTN* | FALSE | FALSE | TRUE |
| *SPRYD4* | FALSE | FALSE | TRUE |
| *SPRYD7* | FALSE | FALSE | TRUE |
| *SPTLC1* | FALSE | FALSE | TRUE |
| *SPTSSA* | FALSE | FALSE | TRUE |
| *SRA1* | FALSE | FALSE | TRUE |
| *SRD5A3* | FALSE | FALSE | TRUE |
| *SREK1* | FALSE | FALSE | TRUE |
| *SREK1IP1* | FALSE | FALSE | TRUE |
| *SRFBP1* | FALSE | FALSE | TRUE |
| *SRGN* | FALSE | FALSE | TRUE |
| *SRP19* | FALSE | FALSE | TRUE |
| *SRP54* | FALSE | FALSE | TRUE |
| *SRP68* | FALSE | FALSE | TRUE |
| *SRP9* | FALSE | FALSE | TRUE |
| *SRPRB* | FALSE | FALSE | TRUE |
| *SRRT* | FALSE | FALSE | TRUE |
| *SRSF2* | FALSE | FALSE | TRUE |
| *SRSF3* | FALSE | FALSE | TRUE |
| *SRSF7* | FALSE | FALSE | TRUE |

| | | | |
|---|---|---|---|
| *SRXN1* | FALSE | FALSE | TRUE |
| *SSB* | FALSE | FALSE | TRUE |
| *SSR1* | FALSE | FALSE | TRUE |
| *SSR3* | FALSE | FALSE | TRUE |
| *SSSCA1* | FALSE | FALSE | TRUE |
| *ST3GAL4* | FALSE | FALSE | TRUE |
| *ST8SIA4* | FALSE | FALSE | TRUE |
| *STAG1* | FALSE | FALSE | TRUE |
| *STAM2* | FALSE | FALSE | TRUE |
| *STARD4* | FALSE | FALSE | TRUE |
| *STAT3* | FALSE | FALSE | TRUE |
| *STAU1* | FALSE | FALSE | TRUE |
| *STIP1* | FALSE | FALSE | TRUE |
| *STK40* | FALSE | FALSE | TRUE |
| *STOML1* | FALSE | FALSE | TRUE |
| *STRAP* | FALSE | FALSE | TRUE |
| *STRBP* | FALSE | FALSE | TRUE |
| *STRN3* | FALSE | FALSE | TRUE |
| *STRN4* | FALSE | FALSE | TRUE |
| *STT3A* | FALSE | FALSE | TRUE |
| *STX11* | FALSE | FALSE | TRUE |
| *STX18* | FALSE | FALSE | TRUE |
| *SUB1* | FALSE | FALSE | TRUE |
| *SUCLG1* | FALSE | FALSE | TRUE |
| *SUGT1* | FALSE | FALSE | TRUE |
| *SUMO1* | FALSE | FALSE | TRUE |
| *SUPT5H* | FALSE | FALSE | TRUE |
| *SUPT6H* | FALSE | FALSE | TRUE |
| *SUPV3L1* | FALSE | FALSE | TRUE |
| *SURF2* | FALSE | FALSE | TRUE |
| *SWI5* | FALSE | FALSE | TRUE |
| *SYAP1* | FALSE | FALSE | TRUE |
| *SYP* | FALSE | FALSE | TRUE |
| *TACC1* | FALSE | FALSE | TRUE |
| *TAF12* | FALSE | FALSE | TRUE |
| *TAF1D* | FALSE | FALSE | TRUE |
| *TAF5* | FALSE | FALSE | TRUE |
| *TAF9B* | FALSE | FALSE | TRUE |
| *TARDBP* | FALSE | FALSE | TRUE |
| *TATDN1* | FALSE | FALSE | TRUE |
| *TBK1* | FALSE | FALSE | TRUE |
| *TBKBP1* | FALSE | FALSE | TRUE |
| *TBL1X* | FALSE | FALSE | TRUE |
| *TBL2* | FALSE | FALSE | TRUE |

| | | | |
|---|---|---|---|
| *TBPL1* | FALSE | FALSE | TRUE |
| *TCEA1* | FALSE | FALSE | TRUE |
| *TCEB1* | FALSE | FALSE | TRUE |
| *TCEB3* | FALSE | FALSE | TRUE |
| *TCOF1* | FALSE | FALSE | TRUE |
| *TEX10* | FALSE | FALSE | TRUE |
| *TEX30* | FALSE | FALSE | TRUE |
| *TFAM* | FALSE | FALSE | TRUE |
| *TFB1M* | FALSE | FALSE | TRUE |
| *TFB2M* | FALSE | FALSE | TRUE |
| *TFCP2* | FALSE | FALSE | TRUE |
| *TFIP11* | FALSE | FALSE | TRUE |
| *TGS1* | FALSE | FALSE | TRUE |
| *THADA* | FALSE | FALSE | TRUE |
| *THAP1* | FALSE | FALSE | TRUE |
| *THAP2* | FALSE | FALSE | TRUE |
| *THAP5* | FALSE | FALSE | TRUE |
| *THOC3* | FALSE | FALSE | TRUE |
| *THRAP3* | FALSE | FALSE | TRUE |
| *TIAM2* | FALSE | FALSE | TRUE |
| *TIGD6* | FALSE | FALSE | TRUE |
| *TIMM17B* | FALSE | FALSE | TRUE |
| *TIMM23B* | FALSE | FALSE | TRUE |
| *TIMM8A* | FALSE | FALSE | TRUE |
| *TLE3* | FALSE | FALSE | TRUE |
| *TM9SF2* | FALSE | FALSE | TRUE |
| *TM9SF3* | FALSE | FALSE | TRUE |
| *TMED1* | FALSE | FALSE | TRUE |
| *TMED3* | FALSE | FALSE | TRUE |
| *TMED9* | FALSE | FALSE | TRUE |
| *TMEM109* | FALSE | FALSE | TRUE |
| *TMEM126A* | FALSE | FALSE | TRUE |
| *TMEM126B* | FALSE | FALSE | TRUE |
| *TMEM150A* | FALSE | FALSE | TRUE |
| *TMEM173* | FALSE | FALSE | TRUE |
| *TMEM180* | FALSE | FALSE | TRUE |
| *TMEM184C* | FALSE | FALSE | TRUE |
| *TMEM185A* | FALSE | FALSE | TRUE |
| *TMEM2* | FALSE | FALSE | TRUE |
| *TMEM237* | FALSE | FALSE | TRUE |
| *TMEM241* | FALSE | FALSE | TRUE |
| *TMEM38B* | FALSE | FALSE | TRUE |
| *TMEM39A* | FALSE | FALSE | TRUE |
| *TMEM41A* | FALSE | FALSE | TRUE |

| | | | |
|---|---|---|---|
| *TMEM5* | FALSE | FALSE | TRUE |
| *TMEM60* | FALSE | FALSE | TRUE |
| *TMEM63B* | FALSE | FALSE | TRUE |
| *TMEM87A* | FALSE | FALSE | TRUE |
| *TMEM99* | FALSE | FALSE | TRUE |
| *TMPO* | FALSE | FALSE | TRUE |
| *TMTC3* | FALSE | FALSE | TRUE |
| *TMX1* | FALSE | FALSE | TRUE |
| *TNFRSF1B* | FALSE | FALSE | TRUE |
| *TNFRSF8* | FALSE | FALSE | TRUE |
| *TNPO1* | FALSE | FALSE | TRUE |
| *TOB2* | FALSE | FALSE | TRUE |
| *TOE1* | FALSE | FALSE | TRUE |
| *TOLLIP* | FALSE | FALSE | TRUE |
| *TOMM20* | FALSE | FALSE | TRUE |
| *TOMM22* | FALSE | FALSE | TRUE |
| *TOP1* | FALSE | FALSE | TRUE |
| *TOR1A* | FALSE | FALSE | TRUE |
| *TOR1B* | FALSE | FALSE | TRUE |
| *TOR4A* | FALSE | FALSE | TRUE |
| *TP53BP1* | FALSE | FALSE | TRUE |
| *TP53BP2* | FALSE | FALSE | TRUE |
| *TPD52L2* | FALSE | FALSE | TRUE |
| *TPM4* | FALSE | FALSE | TRUE |
| *TPRA1* | FALSE | FALSE | TRUE |
| *TRA2B* | FALSE | FALSE | TRUE |
| *TRAF1* | FALSE | FALSE | TRUE |
| *TRAF3* | FALSE | FALSE | TRUE |
| *TRAPPC13* | FALSE | FALSE | TRUE |
| *TRAPPC3* | FALSE | FALSE | TRUE |
| *TRIAP1* | FALSE | FALSE | TRUE |
| *TRIB3* | FALSE | FALSE | TRUE |
| *TRMT10C* | FALSE | FALSE | TRUE |
| *TRMT13* | FALSE | FALSE | TRUE |
| *TRMT44* | FALSE | FALSE | TRUE |
| *TRMT5* | FALSE | FALSE | TRUE |
| *TRMT6* | FALSE | FALSE | TRUE |
| *TRNT1* | FALSE | FALSE | TRUE |
| *TSEN34* | FALSE | FALSE | TRUE |
| *TSN* | FALSE | FALSE | TRUE |
| *TSPAN5* | FALSE | FALSE | TRUE |
| *TSTD2* | FALSE | FALSE | TRUE |
| *TTPAL* | FALSE | FALSE | TRUE |
| *TUBA4A* | FALSE | FALSE | TRUE |

| | | | |
|---|---|---|---|
| *TUBB4A* | FALSE | FALSE | TRUE |
| *TUSC2* | FALSE | FALSE | TRUE |
| *TUT1* | FALSE | FALSE | TRUE |
| *TWISTNB* | FALSE | FALSE | TRUE |
| *TXLNG* | FALSE | FALSE | TRUE |
| *TXNDC17* | FALSE | FALSE | TRUE |
| *TXNDC9* | FALSE | FALSE | TRUE |
| *TXNL1* | FALSE | FALSE | TRUE |
| *TXNL4A* | FALSE | FALSE | TRUE |
| *U2AF1* | FALSE | FALSE | TRUE |
| *U2AF2* | FALSE | FALSE | TRUE |
| *U2SURP* | FALSE | FALSE | TRUE |
| *UBA6* | FALSE | FALSE | TRUE |
| *UBE2A* | FALSE | FALSE | TRUE |
| *UBE2D1* | FALSE | FALSE | TRUE |
| *UBE2D3* | FALSE | FALSE | TRUE |
| *UBE2D4* | FALSE | FALSE | TRUE |
| *UBE2E1* | FALSE | FALSE | TRUE |
| *UBE2I* | FALSE | FALSE | TRUE |
| *UBE2J2* | FALSE | FALSE | TRUE |
| *UBE2K* | FALSE | FALSE | TRUE |
| *UBE2L3* | FALSE | FALSE | TRUE |
| *UBE2N* | FALSE | FALSE | TRUE |
| *UBE2O* | FALSE | FALSE | TRUE |
| *UBE2S* | FALSE | FALSE | TRUE |
| *UBE3A* | FALSE | FALSE | TRUE |
| *UBE3D* | FALSE | FALSE | TRUE |
| *UBFD1* | FALSE | FALSE | TRUE |
| *UBIAD1* | FALSE | FALSE | TRUE |
| *UBQLN1* | FALSE | FALSE | TRUE |
| *UBR7* | FALSE | FALSE | TRUE |
| *UBTD2* | FALSE | FALSE | TRUE |
| *UEVLD* | FALSE | FALSE | TRUE |
| *UHRF1* | FALSE | FALSE | TRUE |
| *UMPS* | FALSE | FALSE | TRUE |
| *UNC50* | FALSE | FALSE | TRUE |
| *UPF1* | FALSE | FALSE | TRUE |
| *UQCC2* | FALSE | FALSE | TRUE |
| *UQCRC1* | FALSE | FALSE | TRUE |
| *UQCRC2* | FALSE | FALSE | TRUE |
| *UQCRFS1* | FALSE | FALSE | TRUE |
| *URM1* | FALSE | FALSE | TRUE |
| *USB1* | FALSE | FALSE | TRUE |
| *USP1* | FALSE | FALSE | TRUE |

| | | | |
|---|---|---|---|
| *USP10* | FALSE | FALSE | TRUE |
| *USP12* | FALSE | FALSE | TRUE |
| *USP14* | FALSE | FALSE | TRUE |
| *USP16* | FALSE | FALSE | TRUE |
| *USP38* | FALSE | FALSE | TRUE |
| *USP39* | FALSE | FALSE | TRUE |
| *USP45* | FALSE | FALSE | TRUE |
| *USP54* | FALSE | FALSE | TRUE |
| *UTP23* | FALSE | FALSE | TRUE |
| *VAPA* | FALSE | FALSE | TRUE |
| *VASP* | FALSE | FALSE | TRUE |
| *VBP1* | FALSE | FALSE | TRUE |
| *VCP* | FALSE | FALSE | TRUE |
| *VDAC1* | FALSE | FALSE | TRUE |
| *VKORC1L1* | FALSE | FALSE | TRUE |
| *VMA21* | FALSE | FALSE | TRUE |
| *VPS26A* | FALSE | FALSE | TRUE |
| *VPS35* | FALSE | FALSE | TRUE |
| *VPS37A* | FALSE | FALSE | TRUE |
| *VTA1* | FALSE | FALSE | TRUE |
| *VTI1B* | FALSE | FALSE | TRUE |
| *WARS2* | FALSE | FALSE | TRUE |
| *WBP11* | FALSE | FALSE | TRUE |
| *WBSCR16* | FALSE | FALSE | TRUE |
| *WDR20* | FALSE | FALSE | TRUE |
| *WDR33* | FALSE | FALSE | TRUE |
| *WDR47* | FALSE | FALSE | TRUE |
| *WDR61* | FALSE | FALSE | TRUE |
| *WDR74* | FALSE | FALSE | TRUE |
| *WDR76* | FALSE | FALSE | TRUE |
| *WDR83* | FALSE | FALSE | TRUE |
| *WDR92* | FALSE | FALSE | TRUE |
| *WDYHV1* | FALSE | FALSE | TRUE |
| *WFS1* | FALSE | FALSE | TRUE |
| *WRB* | FALSE | FALSE | TRUE |
| *WRNIP1* | FALSE | FALSE | TRUE |
| *XPO4* | FALSE | FALSE | TRUE |
| *XRCC6* | FALSE | FALSE | TRUE |
| *XRN2* | FALSE | FALSE | TRUE |
| *XRRA1* | FALSE | FALSE | TRUE |
| *YDJC* | FALSE | FALSE | TRUE |
| *YEATS4* | FALSE | FALSE | TRUE |
| *YIF1A* | FALSE | FALSE | TRUE |
| *YIPF2* | FALSE | FALSE | TRUE |

| | | | |
|---|---|---|---|
| *YIPF6* | FALSE | FALSE | TRUE |
| *YME1L1* | FALSE | FALSE | TRUE |
| *YRDC* | FALSE | FALSE | TRUE |
| *YTHDF2* | FALSE | FALSE | TRUE |
| *ZBTB6* | FALSE | FALSE | TRUE |
| *ZC3H15* | FALSE | FALSE | TRUE |
| *ZC3H18* | FALSE | FALSE | TRUE |
| *ZC3HC1* | FALSE | FALSE | TRUE |
| *ZCCHC17* | FALSE | FALSE | TRUE |
| *ZCCHC6* | FALSE | FALSE | TRUE |
| *ZCRB1* | FALSE | FALSE | TRUE |
| *ZDHHC13* | FALSE | FALSE | TRUE |
| *ZDHHC5* | FALSE | FALSE | TRUE |
| *ZFR* | FALSE | FALSE | TRUE |
| *ZKSCAN2* | FALSE | FALSE | TRUE |
| *ZMAT2* | FALSE | FALSE | TRUE |
| *ZMAT5* | FALSE | FALSE | TRUE |
| *ZNF165* | FALSE | FALSE | TRUE |
| *ZNF17* | FALSE | FALSE | TRUE |
| *ZNF189* | FALSE | FALSE | TRUE |
| *ZNF195* | FALSE | FALSE | TRUE |
| *ZNF207* | FALSE | FALSE | TRUE |
| *ZNF267* | FALSE | FALSE | TRUE |
| *ZNF271* | FALSE | FALSE | TRUE |
| *ZNF273* | FALSE | FALSE | TRUE |
| *ZNF280C* | FALSE | FALSE | TRUE |
| *ZNF286A* | FALSE | FALSE | TRUE |
| *ZNF326* | FALSE | FALSE | TRUE |
| *ZNF330* | FALSE | FALSE | TRUE |
| *ZNF410* | FALSE | FALSE | TRUE |
| *ZNF473* | FALSE | FALSE | TRUE |
| *ZNF526* | FALSE | FALSE | TRUE |
| *ZNF542P* | FALSE | FALSE | TRUE |
| *ZNF544* | FALSE | FALSE | TRUE |
| *ZNF561* | FALSE | FALSE | TRUE |
| *ZNF576* | FALSE | FALSE | TRUE |
| *ZNF579* | FALSE | FALSE | TRUE |
| *ZNF593* | FALSE | FALSE | TRUE |
| *ZNF644* | FALSE | FALSE | TRUE |
| *ZNF653* | FALSE | FALSE | TRUE |
| *ZNF668* | FALSE | FALSE | TRUE |
| *ZNF669* | FALSE | FALSE | TRUE |
| *ZNF672* | FALSE | FALSE | TRUE |
| *ZNF684* | FALSE | FALSE | TRUE |

| | | | |
|---|---|---|---|
| *ZNF7* | FALSE | FALSE | TRUE |
| *ZNF710* | FALSE | FALSE | TRUE |
| *ZNF74* | FALSE | FALSE | TRUE |
| *ZNF746* | FALSE | FALSE | TRUE |
| *ZNF765* | FALSE | FALSE | TRUE |
| *ZNF778* | FALSE | FALSE | TRUE |
| *ZNF787* | FALSE | FALSE | TRUE |
| *ZNRF1* | FALSE | FALSE | TRUE |
| *ZPR1* | FALSE | FALSE | TRUE |
| *ZSCAN12* | FALSE | FALSE | TRUE |
| *ZSCAN5A* | FALSE | FALSE | TRUE |
| *ZSCAN9* | FALSE | FALSE | TRUE |
| *ZUFSP* | FALSE | FALSE | TRUE |

**Note: 'TRUE' denotes that one upregulated gene is regulated by the indicated factor.**
